# Alkyl Radical Generation via C–C Bond Cleavage in 2-Substituted Oxazolidines

**DOI:** 10.1021/acscatal.2c03768

**Published:** 2022-09-29

**Authors:** Adrián Luguera Ruiz, Marta La Mantia, Daniele Merli, Stefano Protti, Maurizio Fagnoni

**Affiliations:** † PhotoGreen Lab, Department of Chemistry, University of Pavia, Viale Taramelli 12, 27100 Pavia, Italy; ‡ Department of Chemistry, University of Pavia, Viale Taramelli 12, 27100 Pavia, Italy

**Keywords:** C−C bond cleavage, conjugate addition, metal-free reaction, oxazolidines, photoorganocatalysis

## Abstract

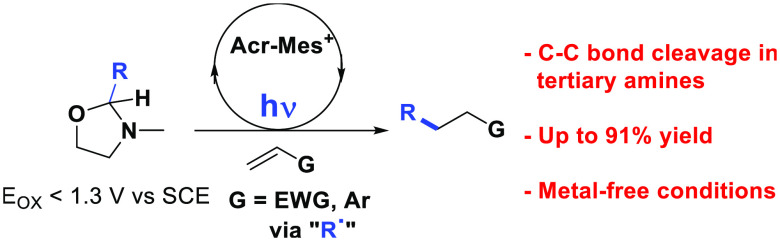

There is an urgent need to develop uncharged radical precursors to be activated under mild photocatalyzed conditions. 2-Substituted-1,3-oxazolidines (*E*_ox_ < 1.3 V vs SCE, smoothly prepared from the corresponding aldehydes) have been herein employed for the successful release of tertiary, α-oxy, and α-amido radicals under photo-organo redox catalysis. The reaction relies on the unprecedented C–C cleavage occurring from the radical cation of these heterocyclic derivatives. Such a protocol is applied to the visible-light-driven conjugate radical addition onto Michael acceptors and vinyl (hetero)arenes under mild metal-free conditions.

## Introduction

**Figure 1:**
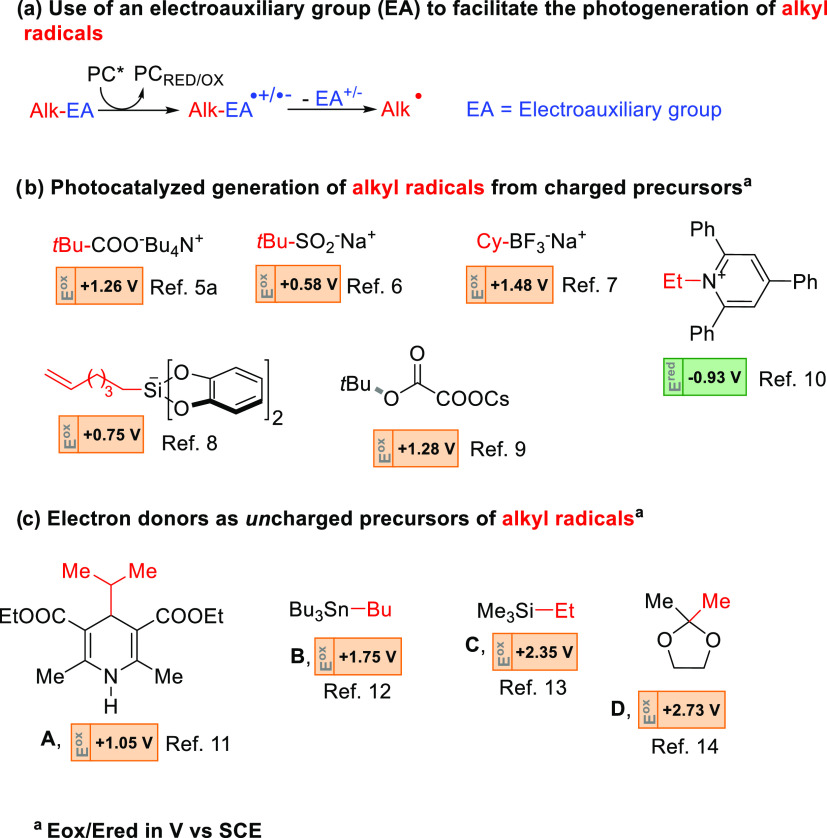
(a) Adoption of an electroauxiliary group (EA) to facilitate the generation of alkyl radicals. (b) Main classes of charged precursors used for photocatalyzed alkyl radical formation. (c) Uncharged precursors as electron donors tested for the release of alkyl radicals.

The photochemical/photocatalyzed approach is nowadays the elective method for the generation of ground-state reactive intermediates^1^ including carbon radicals that can be generated in a mild way using photons as traceless reagents.^2^ In particular, great attention has been given, in the last decade, to the formation of C(sp^3^)–C(sp^3^) bonds via the generation of alkyl radicals,^3^ and several precursors have been devised^3a−3i^ under tin-free conditions.^3e^ In most cases, the alkyl radical is tethered to an electroauxiliary group (EA)^4^ that acts as an electron donor/acceptor moiety. Upon photocatalytic oxidation/reduction, an electrofugal/nucleofugal group (EA^+/–^) is released with the concomitant formation of the alkyl radical (Figure 1a).^3e^ A charged precursor is usually required to facilitate such electron transfer reactions. As shown in Figure 1b, both anionic (e.g., alkyl carboxylates,^5^ alkyl sulfinates,^6^ alkyl trifluoroborates,^7^ bis-catecholato silicates,^8^ and alkyl oxalates^9^) or cationic (e.g., Katritzsky’s salt)^3g,10^ derivatives have been tested.

Due to solubility concerns, however, charged radical precursors can be used only in a limited range of solvents. Curiously, the development of uncharged, easily available radical precursors prone to be oxidized under photocatalyzed conditions is less common. In fact, apart from the case of 1,4-dihydropyridine derivatives (e.g., **A**) that exhibits a low *E*_ox_ value (1.05 V vs SCE),^11^ other neutral donors such as tetraalkyl stannanes (**B**),^12^ tetraalkyl silanes (**C**),^13^ or 2,2-dialkyl 1,3-dioxolanes (**D**)^14^ can be activated only under quite prohibitive conditions (*E*_ox_ up to 2.7 V vs SCE, Figure 1c).

**Scheme 1:**
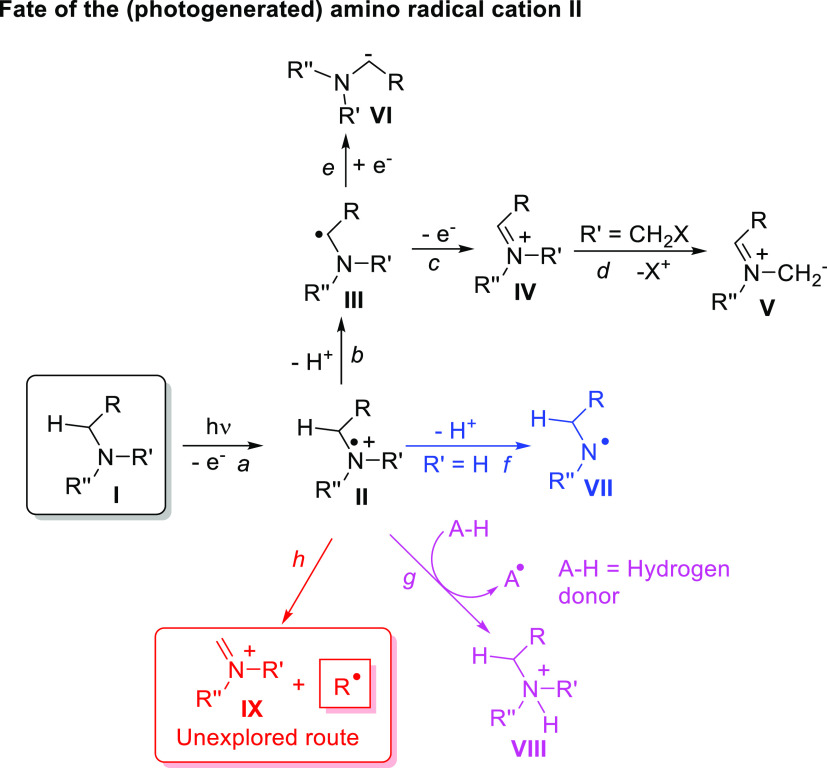
Intermediates Arising from Photogenerated Amine Radical Cations

The available literature points out that one of the elective classes for the design of new uncharged electron donors is certainly that of tertiary amines (*E*_ox_ = 0.83 V vs SCE for triethylamine).^5^ Formerly, such a class of compounds has been largely employed as sacrificial electron donors in photoredox catalysis to reduce a species (or an intermediate) present in solution.^15^ Nevertheless, the formation of acidic^16^ amine radical cations has been extensively employed in synthesis^17^ for the generation of other valuable reactive intermediates, as sketched in Scheme 1. Indeed, radical cation **II** often deprotonates to form a nucleophilic α-amino radical **III** (path b) that may, in turn, undergo oxidation to afford an iminium ion **IV** (path c)^18^ that upon the loss of a positively charged group leads to a 1,3-dipole **V** (path d).^18^ In rare instances, the α-amino radical is photocatalytically reduced to the corresponding anion **VI** (path e).^19^ If the carbons tethered to the nitrogen atom have no hydrogens, deprotonation from the N–H group may take place to give nitrogen-centered radical **VII** (path f).^20^ On the other hand, when intermediate **II** is generated in a tertiary amine that reluctantly loses a proton (e.g., quinuclidine), this species acts instead as an efficient hydrogen atom abstractor (path g).^21^

We were intrigued, however, by the possible C–C cleavage to form stable iminium ion **IX** along with a carbon radical (path h).^22^ Examples of this cleavage are only rarely reported in the literature and point to the requirement of nitrogen-containing heterocycles as ideal substrates.

**Scheme 2:**
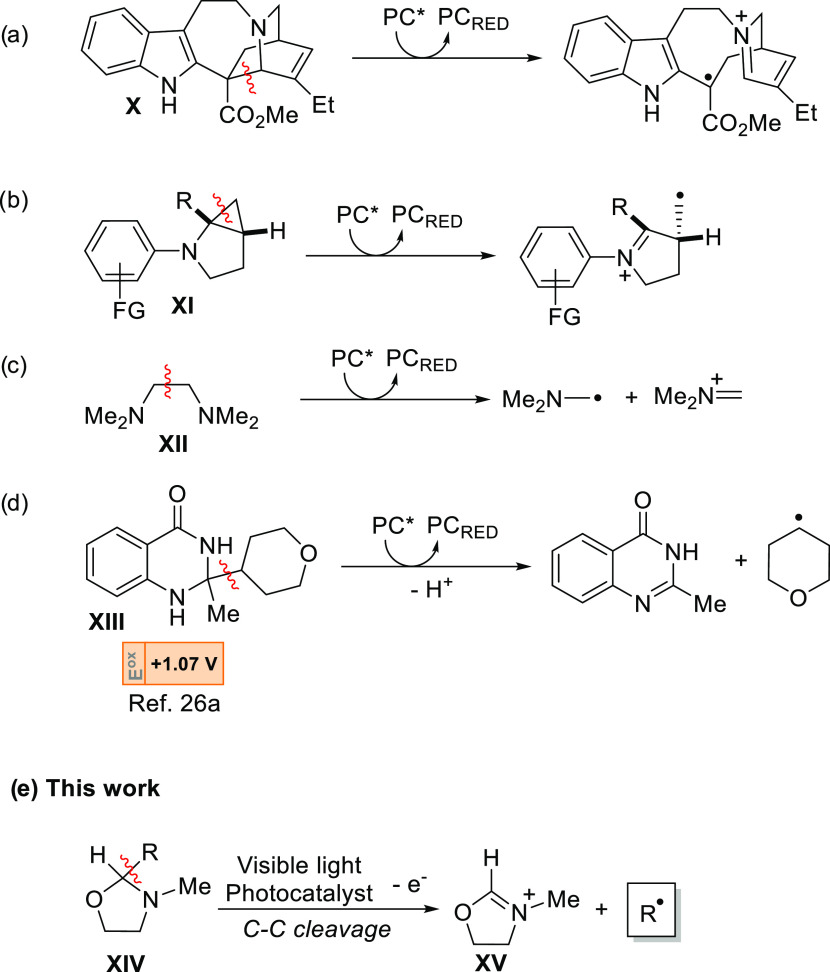
Cleavage of a C–C Bond from a Radical Cation of an Amine

The photocatalyzed single-electron oxidation of (+)-catharanthine **X** indeed induces a C–C bond cleavage in the azabicyclo[2.2.2]oct-5-ene core (Scheme 2a), and the so-modified skeleton of the alkaloid is employed in the preparation of further natural compounds.^17a,23^ In another instance, the cyclopropyl group in bicyclic cyclopropylamines **XI** was easily opened upon photocatalyzed oxidative conditions (Scheme 2b).^24^ The oxidation of tetramethylethanediamine **XII** led to the generation of an iminium ion and an α-amino radical from the fragmentation of the resulting radical cation (Scheme 2c), but the thus obtained radical was applied exclusively to the polymerization of 2-hydroxyethylacrylate.^25^ To our knowledge, however, only dihydroquinazolinones (e.g., **XIII**, Scheme 2d) are used as nitrogen-based heterocycles for the generation of alkyl radicals by a reductive quenching catalytic cycle.^26^

As for the above, a general method to generate (un)substituted alkyl radicals by C–C cleavage from a tertiary amine is so far lacking. We have identified *N*-methyl oxazolidines (**XIV**, the nitrogen analogues of dioxolanes) as possible candidates to achieve this goal (Scheme 2e). Indeed, such compounds are oxidized easily (*E*_ox_ = 1.22 V vs SCE for 2,2,3-trimethyloxazolidine) and act as good electron donors.^27^ We surmised that the driving force of the cleavage should be the stability of the resulting iminium ion **XV**. The present approach represents a mild alternative route for the generation of radicals starting from nitrogen-based heterocycles easily prepared from widely available aldehydes. On these premises, we investigated 2-substituted *N*-methyl oxazolidines for the smooth generation of alkyl radicals to be used in C(sp^3^)–C(sp^3^) bond formation, as detailed in the following.

## Results and Discussion

Oxazolidines **1a–f** have been easily prepared by treating the corresponding aldehydes with 2-(methylamino)ethanol. Related oxazole **1g** has been likewise prepared by the reaction of pivalaldehyde and 2-(methylamino)phenol (see the Supporting Information and Scheme S1 for further details). As shown in Table 1 and Figures S2–S8, compounds **1a–g** exhibited an oxidation potential in the 0.86–1.35 V (vs SCE) range. The *E*_ox_ of oxazolidines is quite independent of the presence of the (substituted) alkyl group, whereas the presence of the aromatic ring in oxazole **1g** made the oxidation of the heterocycle markedly easier (<1 V vs SCE). These low *E*_ox_ values allow us to test several (colored) photocatalysts (PCs) for the occurrence of the desired reaction.

**Table 1:**
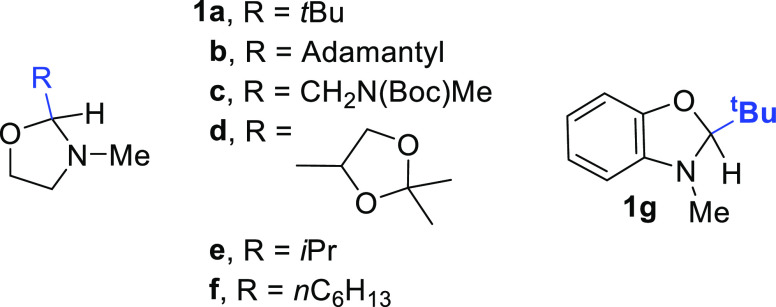
Measured Oxidation Potentials of **1a–g**

compound	*E*_ox_ (V vs SCE)
**1a**	1.33
**1b**	1.26
**1c**	1.23
**1d**	1.22
**1e**	1.35
**1f**	1.19
**1g**	0.86

To test our proposal, we then focused on the *tert-*butylation of dimethylmaleate **2a** using *N*-methyl-2-*tert*butyl-oxazolidine **1a**. We then embarked on an extensive survey of reaction parameters by varying the PC employed (Ir(III)- and Ru(II)-based complexes as well as photo-organo catalysts), the reaction media, the stoichiometric ratio of the reactants, as well as the influence of oxygen in the reaction (see Table S1 for a detailed description of the experiments). A representative list of control experiments is collected in Table 2.

**Table 2:**
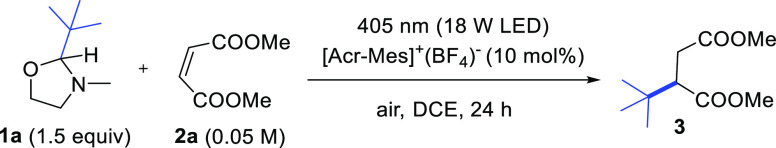
Deviations from the Standard Conditions^a^

entry	deviations from the standard conditions	3 (% yield)
1	none	88
2	4CzIPN (10 mol %), N_2_ atmosphere	34
3	DCM as the solvent	52
4	MeOH as the solvent	5
5	N_2_ atmosphere	71
6	no light	0

a See Table S1 in the Supporting Information for a detailed optimization of the standard conditions.

Gratifyingly, by adopting the conditions described in Table 1 (entry 1), succinate **3** was isolated in an 88% yield. In detail, we found that the best reaction conditions were as follows: an air-equilibrated DCE solution of **2a** (0.05 M) in the presence of 1.5 equiv of **1a**, Acr-Mes^+^ BF_4_^–^ (10 mol %), irradiated at 405 nm for 24 h (Figure S1). Less satisfactory results were obtained when replacing Acr-Mes^+^ BF_4_ (*E*_RED_* > 1.88 V vs SCE)^28^ with 4CzIPN (*E*_RED_* > 1.38 V vs SCE^28^ in MeCN, entry 2) or other metal-free or metal-based PCs (Table S1). The reaction carried out in neat protic solvents (Table 1, entry 4) or in the absence of oxygen (entry 5) led to a decrease in the overall yield. Control experiments confirm the photochemical nature of the process (entry 6). The alkylation yield dropped to 13% when the reaction was carried out in the presence of TEMPO (1 equiv, Table S1, entry 15). The reaction carried out in CD_2_Cl_2_ did not show any deuterium incorporation in compound **3** in analogy with the same reaction occurring in DCM (Figures S9 and S10).

**Scheme 3:**
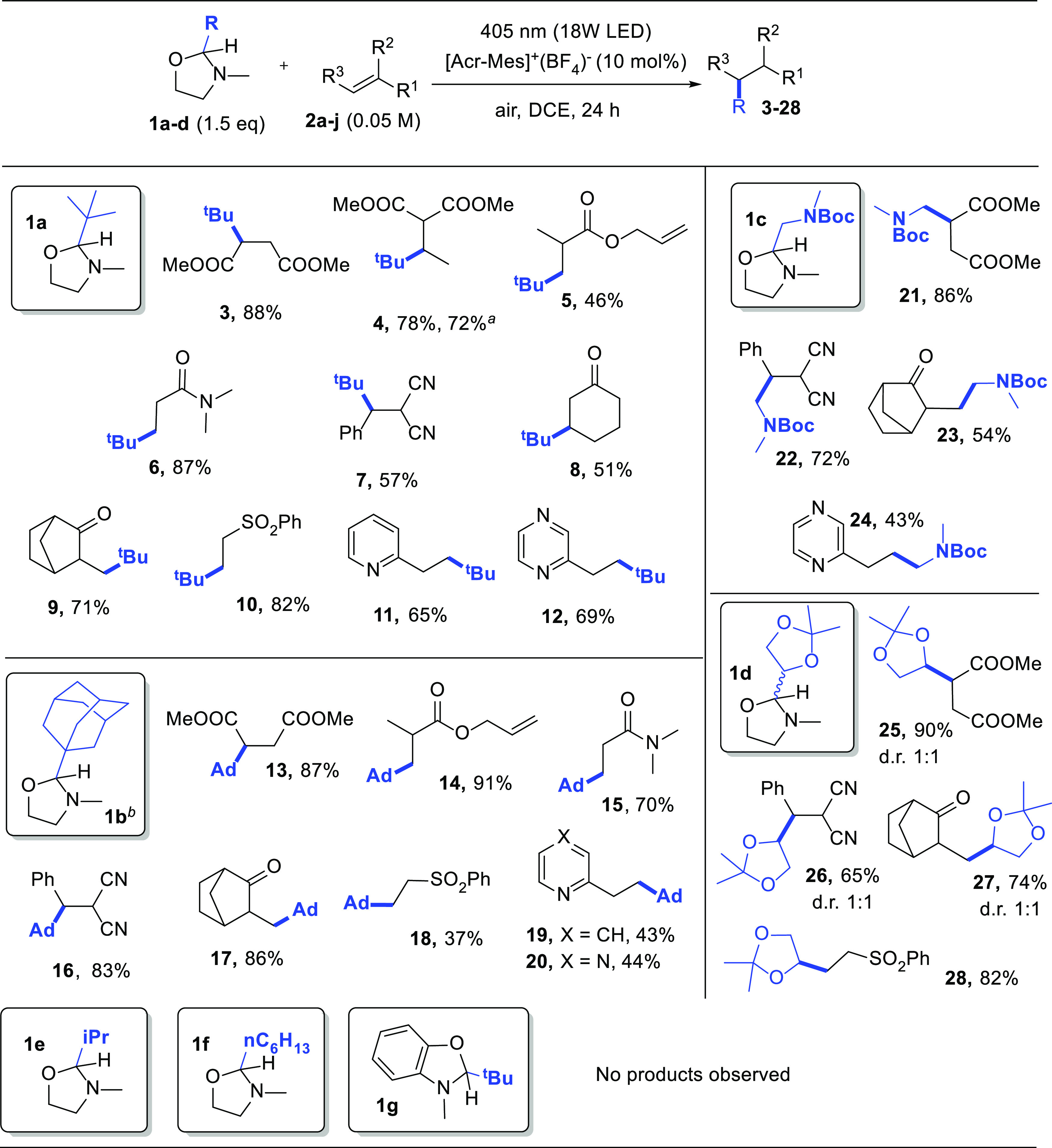
Photoredox Catalyzed Alkylation of Olefins **2a–j** Reaction carried out on a 1 mmol scale. Reactions with oxazolidine **1b** were carried out in a DCE/MeOH 5:1 mixture for solubility concerns.

The scope of the reaction has been then extended to electron-poor alkenes **2b–h** and vinyl heteroarenes **2i and 2j**. The results obtained have been depicted in Scheme 3. *tert*-Butylated derivatives **3–12** have been obtained in good to satisfactory yields. In one case (**4**), the reaction was repeated on a mmol scale. Allyl-methacrylate **2c** was regioselectivity *tert*-butylated on the electrophilic C=C bond, but ester **5** was isolated in only a 46% yield due to its volatility. The method shows a good tolerance in the presence of different functional groups including esters, nitriles, amides, carbonyls, and even heteroarenes. Similar satisfactory results have been obtained when using oxazolidines **1b–d**. In particular, **1b** was adopted to incorporate the adamantyl moiety into olefins, and the resulting adducts have been isolated in up to a 91% yield (e.g., for **14**). In this case, methanol (20% v/v) was added to completely dissolve **1b**. To our delight, we found that the release of substituted alkyl radicals such as α-amido (from **1c**) and α-oxy (from glyceraldehyde derivative **1d**) led to alkylated products **21–28** in the 43–90% range (Scheme 3). Unfortunately, no alkylation products were detected when **1e and f** and aromatic derivative **1g** were used as the radical precursors.

This is an appealing approach for the generation of tertiary (e.g., *t*Bu and adamantyl) and α-oxy and α-amido carbon-centered radicals. The reaction took place upon visible light using a commercially available and widely employed organic dye (Acr-Mes^+^BF_4_^–^) as the photoredox catalyst and gives access to a large variety of alkylated compounds, including, among others, β-alkyl-amides, nitriles, and ketones, as well as functionalized nitrogen-based heterocycles via formation of a C(sp^3^)–C(sp^3^) bond.

The preparation of **3–20** allows for the introduction of a quaternary carbon in an organic molecule by the forging of a C(sp^3^)–C(sp^3^) bond, a topic for which there is great interest in view of all-carbon quaternary scaffolds present in many biologically active compounds.^29^ Moreover, the adamantylation of olefin is an important strategy to incorporate a moiety able to impart steric bulkiness, chemical inertness, rigidity, and lipophilicity to an organic compound; indeed, several adamantane-based drugs are known to take advantage of these peculiarities.^30^ The design of catalysts having the adamantane scaffold is also another hot topic.^31^

As for the above, finding new methods for the formation of tertiary radicals and their application is of utmost importance.^3^ The photogeneration of these radicals has been only sparsely reported using Barton esters,^32^*N*-(acyloxy)phthalimides,^33^ alkyl *N*-phthalimidoyl oxalates,^34^ and alkyl carboxylates.^5b^ Thermal generation of these intermediates involved electrophiles such as alkyl halides^35^ or alkylsulfones,^36^ despite that in some cases, the desired C(sp^3^)–C(sp^3^) bond formation failed to occur.^37^

**Scheme 4:**
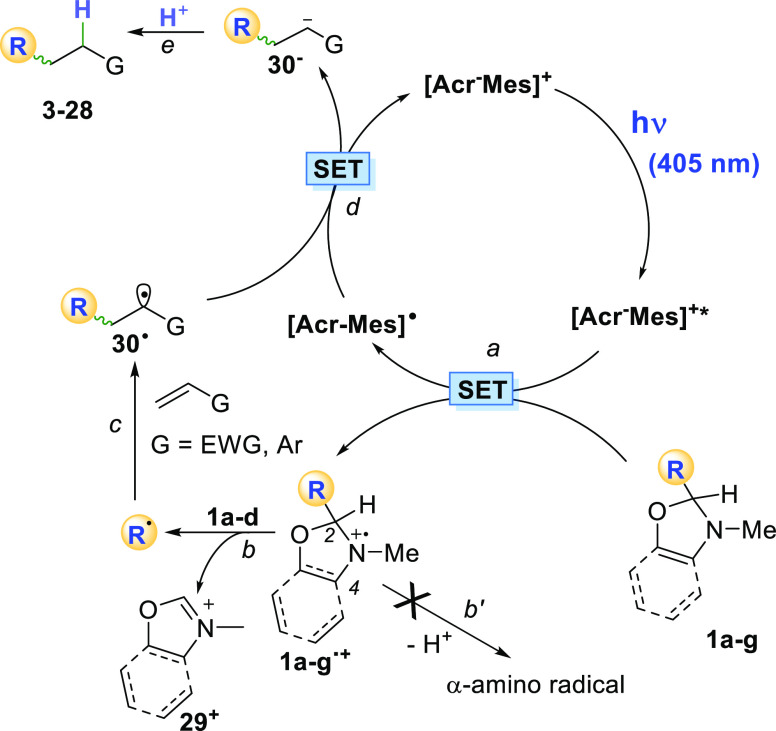
Suggested Mechanism

A tentative mechanism for the process illustrated in the present manuscript is proposed in Scheme 4.

Compounds **1a–g** are radical precursors having an *E*_ox_ < 1.3 V vs SCE (Table 1), comparable to that of other uncharged 1,4-dihydropyridine derivatives (Figure 1c).^11^ The monoelectronic oxidation of **1a–g** by the photoexcited acridinium catalyst Acr-Mes^+*^ to give the corresponding radical cations **1a–g**^**•+**^ is thus feasible (path a). At this stage, an unprecedented C–C cleavage in **1a–d**^**•+**^ took place, releasing a carbon-centered radical and a stable iminium ion (**29**^**+**^, path b). The peculiar structure of the oxazolidines avoid the possible α-deprotonation at the radical cation stage from position 2 and 4 as well as from the *N*-Me group to give an α-amino radical (path b’). The driving force of such C–C cleavage is the stability of the tertiary, α-oxy, and α -amido radicals released.

In the case of oxazolidines **1e and f** and oxazole **1g**, the formation of the corresponding radical cation led to an unproductive alkylation. In the former case, the release of a primary or a secondary radical is expected to be not so favored and competitive paths may operate.^15^ The structure of compound **1g**, however, resembles that of an aniline derivative and may suffer, in analogy with *N,N*-dialkyl anilines, of competitive deprotonation^38^ or the reactivity of **1g**^**•+**^ may not have a role due to the efficient back electron transfer with the reduced form of the PC.^39^

The alkyl radicals derived from **1a–d**^**•+**^ are, in turn, trapped by electron-poor olefins or vinyl (hetero)aromatics (path c). Back electron transfer from Acr-Mes^•^ to the adduct radical **30**^**•**^ (path d) followed by protonation (path e) led to the alkylated products while restoring the photoredox catalyst. This agrees with related conjugate radical additions promoted by the acridinium salt.^40^ A hydrogen atom transfer from the solvent by **30**^**•**^ is safely excluded by the deuteration experiments (see Figures S9–S10). The radical nature of the process is confirmed by the detrimental effect induced by the presence of a radical scavenger (TEMPO, see Table S1).

## Conclusions

Summing up, we designed a class of smoothly prepared uncharged precursors for the easy release of alkyl radicals (tertiary, α-oxy, and α-amido) under photoredox catalyzed conditions. This process relies on the unprecedented C–C cleavage in amine radical cations obtained by visible-light irradiation in the presence of commercially available Acr-Mes^+^ BF_4_^–^ as a photo-organocatalyst. This approach was exploited for the introduction, among the others, of a quaternary carbon center via C(sp^3^)–C(sp^3^) bond formation and for valuable adamantylations.

## Supporting Information Available

Materials and methods, experimental procedures, optimization studies, electrochemical measurements, characterization data, NMR spectra, and mass spectrometry data of new compounds (PDF)
