# Supplementary material for: Alkyl Radical Generation via C–C Bond Cleavage in 2-Substituted Oxazolidines
Source: ACS Catal. 2022 Sep 29;12(19):12469–76. doi: 10.1021/acscatal.2c03768 (PMC9552967; doi:10.1021/acscatal.2c03768)

Supporting information for

Alkyl Radical Generation via C-C Bond Cleavage in 2-Substituted Oxazolidines

Adrián Luguera Ruiz,[a] Marta La Mantia,[a] Daniele Merli, [b] Stefano Protti, [a,*] Maurizio Fagnoni[a,*]

[a] *PhotoGreen Lab, Department of Chemistry, University of Pavia, Viale Taramelli 12, 27100 Pavia, Italy. E-mail: stefano.protti@unipv.it, fagnoni@unipv.it*

[b] *Department of Chemistry, University of Pavia, Viale Taramelli 12, 27100 Pavia, Italy.*

1. **Experimental part** **S1**
2. **Tables and Figures** **S12**
3. **Deuteration experiments** **S19**
4. **References** **S21**
5. **Copy of ^{1}H and ^{13}C NMR spectra** **S23**

1. Experimental Part

General. ^{1}H and ^{13}C NMR spectra were recorded on a 300 e 75 MHz spectrometer, respectively. The attributions were based on ^{1}H and ^{13}C NMR experiments; chemical shifts are reported in ppm downfield from TMS. GC analysis were performed using a HP SERIES 5890 II equipped with a fire ion detector (FID, temperature 350 °C). Analytes were separated using a Restek Rtx-5MS (30 m×0.25 mm×0.25 µm) capillary column with nitrogen as a carrier gas at 1 mL min^{-1}. The injector temperature was 250 °C. The GC oven temperature was held at 80 °C for 2 min, increased to 250 °C by a temperature ramp of 10 °C min^{-1}, and held for 10 min. HRMS data were acquired using a X500B QTOF System (SCIEX, Framingham, MA 01701 USA) available at the CGS of the University of Pavia, equipped with the Twin Sprayer ESI probe and coupled to an ExionLC™ system (SCIEX). The SCIEX OS software 2.1.6 was used as operating platform. For MS detection the following parameters were applied: Curtain gas 30 psi, Ion source gas 1 45 psi, Ion source gas 2 55 psi, Temperature 450°C, Polarity negative, Ion spray voltage -4500 V, TOF mass range 50-1600 Da, declustering potential -60 V and collision energy -10 V.' The electrochemical characterization (CV) of compound **1a-g** was carried out by means of a Amel model 4330 module equipped with a 20 mL standard three-electrode cell with a glassy carbon (0.49 cm^2 geometrical area) working electrode, a platinum wire as auxiliary electrode and a Ag/AgCl, 3 M NaCl reference electrode, all obtained from BASi Electrochemistry. Acetonitrile containing 0.1 M lithium perchlorate were used as solvent and supporting electrolyte, scanning the potential in the range from 0 mV to + 2500 mV, with a 5 mM compound concentration and a scan speed of 50 mV/s.

General Procedure for the Synthesis of 2-Substituted *N*-Methyl Oxazolidines and Oxazoles. Oxazolidines **1a-f** and oxazole **1g** were synthesized and fully characterized by our research group (see Scheme S1). 2-(Methylamino)ethanol or 2-(methylamino)phenol (1 equiv) was added to a suspension of $MgSO_4$ (25 mg mmol^{-1}) and the corresponding aldehyde (1 equiv) in Et_2O (1.7 mL mmol^{-1}). The mixture was stirred and heated at reflux overnight. The crude mixture was diluted with DCM and filtered, and the obtained solution was evaporated affording the desired oxazolidine in high purity. No further purification was needed.

Scheme S1: Synthesis of oxazolidines **1a-g.**

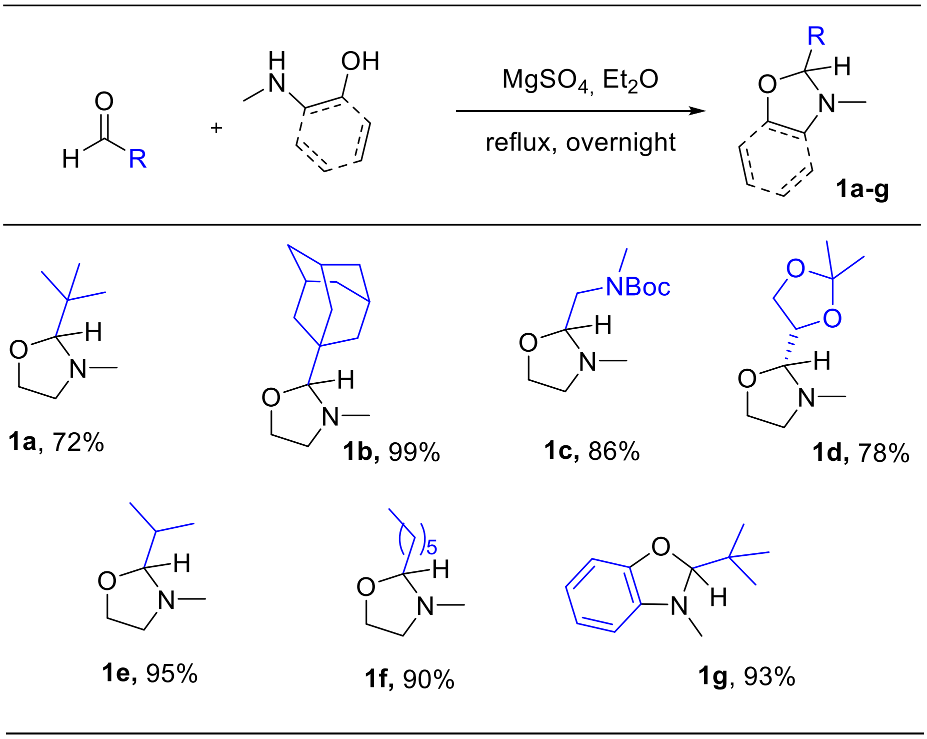

2-*tert*-Butyl-3-methyloxazolidine (1a). From *N*-methylaminoethanol (11.6 mmol, 1 equiv, 0.93 mL), $MgSO_4$ (314 mg), pivalaldehyde (11.6 mmol, 1 equiv, 1.3 mL) in Et_2O (20 mL). The crude mixture was diluted with DCM, filtered off and the obtained solution was concentrated in vacuo affording **1a** (72% yield, colourless oil) that was used without further purification.

1a. ^{1}H NMR (300 MHz, Acetone-*d*$_6$) δ 3.74 (ddt, *J* = 8.8, 6.7, 4.4 Hz, 1H), 3.71–3.60 (m, 2H), 3.01 (ddd, *J* = 10.4, 7.6, 6.0 Hz, 1H), 2.64 (ddd, *J* = 10.4, 5.9, 4.5 Hz, 1H), 2.41 (s, 3H), 0.85 (s, 9H). ^{13}C NMR (75 MHz, Acetone-*d*$_6$) δ 106.6, 65.3, 56.4, 44.5, 37.1, 25.5. HRMS (EI) m/z: $[M+H]^+$ calculated for $C_8H_{17}NO$ 143.1383, found 143.1379.

2-Adamantan-1-yl)-3-methyloxazolidine (1b). From *N*-methylaminoethanol (6.1 mmol, 1 equiv, 0.49 mL), $MgSO_4$ (153 mg) Adamantane-1-carbaldehyde[S1] (6.1 mmol, 1 equiv, 1 g) in Et_2O (10 mL). The crude mixture was diluted with DCM, filtered off and the obtained solution was concentrated in vacuo affording **1b** (99% yield, oil) that was employed without further purification.

1b. ^{1}H NMR (300 MHz, Acetone-*d*$_6$) δ 3.80–3.57 (m, 2H), 3.50 (s, 1H), 2.95 (dt, *J* = 10.3, 6.9 Hz, 1H), 2.63 (dt, *J* = 10.3, 5.1 Hz, 1H), 2.39 (s, 3H), 2.00–1.85 (m, 3H), 1.83–1.42 (m, 13H). ^{13}C NMR (75 MHz, acetone-*d*$_6$) δ 106.8, 65.2, 56.4, 44.8, 39.1, 38.4, 38.1, 29.1, 29.0. HRMS (EI) m/z: $[M+H]^+$ calculated for $C_{14}H_{23}NO$ 222.1852, found 222.1841.

***tert*-Butyl methyl((3-methyloxazolidin-2-yl)methyl)carbamate (1c).**

From *N*-methylaminoethanol (2.9 mmol, 1 equiv, 0.23 mL), $MgSO_4$ (75 mg), *N*-Boc-2-aminoacetaldehyde (2.9 mmol, 1 equiv. 0.48 mL) in Et_2O (5 mL). The crude mixture was diluted with DCM, filtered off and the obtained solution was concentrated in vacuo affording **1c** (86% yield, pale yellow oil) that was used without further purification.

1c. ^{1}H NMR (300 MHz, Acetone- d_6) δ 1.42 (s, 9H), 2.37 (s, 3H), 2.49–2.58 (q, 1H, J = 9 Hz), 2.81–2.84 (m, 1H), 2.85 (s, 3H), 3.42–3.48 (dd, 1H, J = 3 and 15 Hz), 3.76–3.80 (m, 2H), 3.95–3.99 (m, 1H). ^{13}C NMR (75 MHz, Acetone- d_6) δ 148.1, 98.0, 79.6, 65.4, 55.2, 52.9, 40.6, 28.9, 30.1. HRMS (EI) m/z: $[M+H]^+$ calculated for $C_{11}H_{22}N_2O_3$ 231.1703, found 231.1699.

2-(2,2-Dimethyl-1,3-dioxolan-4-yl)-3-methyloxazolidine (1d). From *N*-methylaminoethanol (7.7 mmol, 1 equiv, 0.62 mL), $MgSO_4$ (193 mg), 2,2-dimethyl-1,3-dioxolane-4-carbaldehyde (7.7 mmol, 1 equiv. 0.96 mL) in Et_2O (13 mL). The crude mixture was diluted with DCM, filtered off and the obtained solution was concentrated in vacuo affording **1d** (78% yield, pale yellow oil), that was used without further purification.

1d. ^{1}H NMR (200 MHz, Acetone-d_6) δ 4.10–4.02 (m, 1H), 4.01–3.92 (m, 2H), 3.85–3.73 (m, 3H), 3.21–3.07 (m, 1H), 2.60–2.46 (m, 1H), 2.42 (s, 3H), 1.34 (s, 3H), 1.27 (s, 3H). ^{13}C NMR (75 MHz, Acetone-d_6) δ 110.2, 98.9, 98.4, 79.1, 78.4, 67.1, 66.7, 66.2, 65.4, 56.0, 55.7, 42.8, 40.8, 27.3, 27.1, 26.1, 26.0. HRMS (EI) m/z: $[M+H]^+$ calculated for $C_9H_{17}NO_3$ 188.1281, found 188.1276.

2-Isopropyl-3-methyloxazolidine (1e): From *N*-methylaminoethanol (13.9 mmol, 1 equiv., 1.1 mL), $MgSO_4$ (348 mg), 2-methylpropanal (13.9 mmol, 1 equiv. 1.3 mL) in Et_2O (24 mL). The crude mixture was diluted with DCM, filtered off and the obtained solution concentrated in vacuo affording **1e** (95% yield, oil) that was used without further purification. Spectroscopical data are in accordance with the literature.[S2]

1e. ^{1}H NMR (300 MHz, Acetone-d_6) δ 3.82–3.60 (m, 2H), 3.58 (d, J = 4.0 Hz, 1H), 3.09 (ddd, J = 9.5, 6.2, 4.1 Hz, 1H), 2.51 (ddd, J = 9.4, 7.9, 7.2 Hz, 1H), 1.68 (dd, J = 6.8, 4.0 Hz, 1H), 0.91 (d, J = 6.9 Hz, 3H), 0.85 (d, J = 6.7 Hz, 3H). ^{13}C NMR (75 MHz, Acetone- d_6) δ 102.4, 65.0, 55.7, 40.2, 19.1, 16.2. HRMS (EI) m/z: $[M+H]^+$ calculated for $C_7H_{15}NO$ 130.1226, found 130.1224.

2-Hexyl-3-methyloxazolidine (1f). From *N*-methylaminoethanol (7 mmol, 1 equiv, 0.56 mL) was added to a suspension of $MgSO_4$ (185 mg), heptanal (7 mmol, 1 equiv. 0.98 mL) in Et_2O (12 mL). The crude mixture was diluted with DCM, filtered off and the obtained solution was concentrated in

vacuo to give **1f** (90% yield, oil) that was used without further purification. Spectroscopic data are in accordance with the literature.[S3]

1f. ^{1}H NMR (300 MHz, Acetone-*d$_6$*) δ 3.79–3.69 (m, 3H), 3.10 (ddd, *J* = 9.8, 5.8, 4.2 Hz, 1H), 2.49 (dt, *J* = 9.5, 8.0 Hz, 1H), 2.26 (s, 3H), 1.59–1.23 (m, 10H), 0.88 (d, *J* = 6.9 Hz, 3H). ^{13}C NMR (75 MHz, Acetone-*d$_6$*) δ 98.0, 64.6, 55.5, 39.1, 34.1, 32.7, 25.5, 23.3, 14.3. HRMS (EI) m/z: $[M+H]^+$ calculated for $C_{10}H_{21}NO$ 172.1696, found 172.1693.

2-(*tert*-Butyl)-3-methyl-2,3-dihydrobenzo[d]oxazole (1g). From *N*-methylaminophenol (2.9 mmol, 1 equiv., 0.31 mL), $MgSO_4$ (75 mg), 2-(*N*-methylamino)benzaldehyde (2.9 mmol, 1 equiv, 0.32 mL) in Et_2O (5 mL). The crude mixture was diluted with DCM, filtered off and the obtained solution was concentrated in vacuo affording **1g** (93% yield, dark blue oil) that was used without further purification.

1g. ^{1}H NMR (300 MHz, Acetone-*d$_6$*) δ 6.77–6.54 (m, 4H), 4.97 (s, 1H), 2.85 (s, 3H), 0.96 (s, 10H). ^{13}C NMR (75 MHz, Acetone-*d$_6$*) δ 152.1, 152.0, 143.4, 143.4, 121.4, 120.6, 110.1, 109.7, 107.3, 41.2, 38.1, 24.3. HRMS (EI) m/z: $[M+H]^+$ calculated for $C_{12}H_{17}NO$ 192.1383, found 192.1375.

General Procedure for the Synthesis of Compounds 3-28 In a Pyrex glass vessel (see Figure S1), a solution of the corresponding oxazolidine **1a-g** (0.375 mmol, 1.5 equiv, 0.075 M), olefin **2a-j** (0.25 mmol, 1 equiv, 0.05 M) and $[Acr\text{-}Mes]^+(BF_4)^-$ (0.025 mmol, 10 mol%) in DCE (5 mL) was irradiated for 24 h by means of a 18W EvoluChem lamp (emission located at 405 nm). The photolyzed solution was then evaporated and the resulting residue purified by flash column chromatography by means of automated Isolera (Biotage) apparatus and a SiO_2 cartridge.

Dimethyl 2-(*tert*-butyl)succinate (3). From **1a** (0.375 mmol, 1.5 equiv, 0.075 M, 58 µL), dimethyl maleate (**2a,** 0.25 mmol, 1 equiv, 0.05 M, 31 µL), $[Acr\text{-}Mes]^+(BF_4)^-$ (0.025 mmol, 10 mol%, 10 mg) in DCE (5 mL). Purification by column chromatography (eluent: Cyclohexane/AcOEt 100/0 to 4/1) afforded **3** (colourless oil, 44 mg) in 88% yield. Spectroscopic data are in accordance with the literature.[S4]

3. ^{1}H NMR (300 MHz, Chloroform-d) δ 3.69 (s, 3H), 3.66 (s, 3H), 2.87 – 2.39 (m, 3H), 0.96 (s, 9H). ^{13}C NMR (75 MHz, Chloroform-d) δ 174.9, 173.3, 51.9, 51.5, 51.4, 32.8, 29.9, 27.9.

Dimethyl 2-(3,3-dimethylbutan-2-yl)malonate (4). From **1a** (0.375 mmol, 1.5 equiv, 0.075 M, 58 µL), dimethyl 2-ethylidenemalonate (**2b**, 0.25 mmol, 1 equiv, 0.05 M, 35 µL), $[Acr\text{-}Mes]^+(BF_4)^-$ (0.025 mmol, 10 mol%, 10 mg) in DCE (5 mL). Purification by column chromatography (eluent:

Cyclohexane/AcOEt 100/0 to 4/1) gave **4** (pale yellow oil, 42 mg) in 78% yield. Spectroscopic data are in accordance with the literature. Compound **4** was obtained in 72% yield when starting from 1 mmol of **2b**.

4. ^{1}H NMR (300 MHz, Chloroform-d) δ 3.71 (s, 3H), 3.70 (s, 3H), 3.55 (d, *J* = 5.4 Hz, 1H), 2.23 (qui, *J* = 7.1 Hz, 1H), 0.99 (d, *J* = 7.2, 3H), 0.88 (s, 9H). ^{13}C NMR (75 MHz, Chloroform-d) δ 170.5, 169.8, 52.9, 52.4, 51.9, 42.8, 33.4, 27.3, 12.0. HRMS (EI) m/z: $[M+H]^+$ calculated for $C_{11}H_{20}O_4$ 217.1434, found 217.1431.

Allyl 2,4,4-trimethylpentanoate (5). From **1a** (0.375 mmol, 1.5 equiv, 0.075 M, 58 µL), allyl methacrylate (**2c**, 0.25 mmol, 1 equiv, 0.05 M, 33 µL), $[Acr\text{-}Mes]^+(BF_4)^-$ (0.025 mmol, 10 mol%, 10 mg) in DCE (5 mL). Purification by column chromatography (eluent: Cyclohexane/AcOEt 100/0 to 9/1) afforded **5** (colourless oil, 21 mg) in 46% yield.

5. ^{1}H NMR (300 MHz, Chloroform-*d*) δ 6.05–5.79 (m, 1H), 5.41–5.17 (m, 2H), 4.64–4.44 (m, 2H), 2.63–1.98 (m, 2H), 1.95–1.80 (m, 1H), 1.17 (d, *J* = 6.9 Hz, 3H), 0.89 (s, 9H). ^{13}C NMR (75 MHz, Chloroform-*d*) δ 177.7, 132.5, 118.2, 65.3, 65.1, 47.9, 36.4, 29.6, 20.5. Anal. Calcd for $C_{11}H_{20}O_2$ C, 71.70; H, 10.94. Found 71.7; 10.8. GC-MS (m/z): 184.3 (20, M^+), 127.3 (100), 109.2 (20), 97.3 (10).

***N,N*,4,4-tetramethylpentanamide (6).** From **1a** (0.375 mmol, 1.5 equiv, 0.075 M, 58 µL), *N,N*-dimethylacrylamide (**2d**, 0.25 mmol, 1 equiv, 0.05 M, 25 µL), $[Acr\text{-}Mes]^+(BF_4)^-$ (0.025 mmol, 10 mol%, 10 mg) in DCE (5 mL). Purification by column chromatography (eluent: DCM/MeOH 100/0 to 95/5) gave **6** (colourless oil, 34.3 mg) in 87% yield. Spectroscopic data are in accordance with the literature.[S5]

6. ^{1}H NMR (300 MHz, Chloroform-d) δ 3.01 (s, 6H), 2.43 – 2.32 (m, 2H), 1.63 – 1.47 (m, 2H), 0.92 (s, 9H). ^{13}C NMR (75 MHz, Chloroform-d) δ 171.6, 39.11, 30.2, 29.8, 29.3, 29.1.

2-(2,2-Dimethyl-1-phenylpropyl)malononitrile (7). From **1a** (0.375 mmol, 1.5 equiv, 0.075 M, 58 µL), 2-benzylidenmalononitrile (**2e**, 0.25 mmol, 1 equiv, 0.05 M, 58 µL), $[Acr\text{-}Mes]^+(BF_4)^-$ (0.025 mmol, 10 mol%, 10 mg) in DCE (5 mL). Purification by column chromatography (eluent: Cyclohexane/AcOEt 100/0 to 9/1) afforded **7** (white solid, mp 53.0–54.0 °C, 30.1 mg) in 57% yield. Spectroscopic data are in accordance with the literature.[S6]

7. ^{1}H NMR (300 MHz, acetone-d_6) δ 7.55 – 7.47 (m, 2H), 7.47 – 7.33 (m, 3H), 5.07 (d, *J* = 6.4 Hz, 1H), 3.33 (d, *J* = 6.4 Hz, 1H), 1.11 (s, 9H). ^{13}C NMR (75 MHz, acetone-d_6) δ 138.4, 130.4, 129.2, 129.0, 115.3, 115.1, 56.4, 35.5, 28.7, 25.7.

3-(*tert*-Butyl)cyclohexan-1-one (8). From **1a** (0.375 mmol, 1.5 equiv, 0.075 M, 58 µL), cyclohexenone (**2f**, 0.25 mmol, 1 equiv, 0.05 M, 24 µL), $[Acr\text{-}Mes]^+(BF_4)^-$ (0.025 mmol, 10 mol%, 10 mg) in DCE (5 mL). Purification by column chromatography (eluent: Cyclohexane/AcOEt 100/0 to 9/1) afforded **8** (colourless oil, 19.5 mg) in 51% yield. Spectroscopic data are in accordance with the literature.[S7]

8. ^{1}H NMR (300 MHz, Chloroform-*d*) δ 2.49-2.40 (m, 1H), 2.39–2.30 (m, 1H), 2.29–2.20 (m, 1H), 2.17 (s, 1H), 2.14–1.99 (m, 2H), 1.99–1.89 (m, 1H), 1.38–1.23 (m, 2H), 0.89 (s, 9H). ^{13}C NMR (75 MHz, Chloroform-*d*) 13C NMR (75 MHz, Chloroform-*d*) δ 213.2, 49.5, 43.8, 41.5, 32.9, 27.3, 26.3, 25.8.

3-Neopentylbicyclo[2.2.1]heptan-2-one (9): From **1a** (0.375 mmol, 1.5 equiv, 0.075 M, 58 µL), 3-methylenebicyclo[2.2.1]heptan-2-one (**2g**, 0.25 mmol, 1 equiv, 0.05 M, 28 µL), $[Acr\text{-}Mes]^+(BF_4)^-$ (0.025 mmol, 10 mol%, 10 mg) in DCE (5 mL). Purification by column chromatography (eluent: Cyclohexane/AcOEt 100/0 to 9/1) gave **9** (pale yellow oil, 30.5 mg) in 71% yield. Spectroscopic data are in accordance with the literature.[S8]

9. ^{1}H NMR (300 MHz, Acetone-d_6) δ 2.65 (d, J = 5.1 Hz, 1H), 2.46 (d, J = 5.0 Hz, 1H), 1.89–1.46 (m, 6H), 1.33–1.10 (m, 3H), 0.92 (s, 9H). ^{13}C NMR (75 MHz, Acetone-d_6) δ 218.9, 51.8, 50.4, 41.3, 40.1, 37.7, 31.1, 30.0, 25.9, 22.0. HRMS (EI) m/z: $[M+H]^+$ calculated for $C_{12}H_{20}O$ 181.1587, found 181.1585.

((3,3-Dimethylbutyl)sulfonyl)benzene (10). From **1a** (0.375 mmol, 1.5 equiv, 0.075 M, 58 µL), phenyl vinyl sulfone (**2h,** 0.25 mmol, 1 equiv, 0.05 M, 42 mg), $[Acr\text{-}Mes]^+(BF_4)^-$ (0.025 mmol, 10 mol%, 10 mg) in DCE (5 mL). Purification by column chromatography (eluent: DCM/MeOH 100/0 to 95/5) afforded **10** (pale yellow oil, 46.6 mg) in 82% yield. Spectroscopic data are in accordance with the literature.[S9]

10. ^{1}H NMR (300 MHz, Chloroform-*d*) δ 7.96–7.86 (m, 2H), 7.70–7.51 (m, 3H), 3.12–2.99 (m, 2H), 1.66–1.53 (m, 2H), 0.86 (s, 9H).^{13}C NMR (75 MHz, Chloroform-*d*) δ 139.4, 133.7, 129.4, 128.2, 53.1, 35.8, 30.2, 29.0.

2-(3,3-Dimethylbutyl)pyridine (11). From **1a** (0.375 mmol, 1.5 equiv, 0.075 M, 58 µL), 2-vinylpyridine (**2i**, 0.25 mmol, 1 equiv, 0.05 M, 26 µL), $[Acr\text{-}Mes]^+(BF_4)^-$ (0.025 mmol, 10 mol%, 10 mg) in DCE (5 mL). Purification by column chromatography (eluent: Cyclohexane/AcOEt 100/0 to 8/1) gave **11** (pale yellow oil, 26.7 mg) in 65% yield. Spectroscopic data are in accordance with the literature.[S10]

11. ^{1}H NMR (300 MHz, Chloroform-*d*) δ 8.52 (d, *J* = 5.0 Hz, 1H), 7.60 (td, *J* = 7.7, 1.9 Hz, 1H), 7.17 (d, *J* = 7.9 Hz, 1H), 7.13–7.04 (m, 1H), 2.85–2.72 (m, 2H), 1.70–1.55 (m, 2H), 0.98 (s, 9H). ^{13}C NMR (75 MHz, Chloroform-*d*) δ 163.2, 149.0, 136.8, 122.9, 121., 44.4, 33.9, 30.7, 29.5.

2-(3,3-Dimethylbutyl)pyrazine (12). From **1a** (0.375 mmol, 1.5 equiv, 0.075 M, 58 µL), 2-vinylpyrazine (**2j**, 0.25 mmol, 1 equiv, 0.05 M, 25 µL), $[Acr\text{-}Mes]^+(BF_4)^-$ (0.025 mmol, 10 mol%, 10 mg) in DCE (5 mL). Purification by column chromatography (eluent: Cyclohexane/AcOEt 100/0 to 4/1) gave **12** (pale yellow oil, 28.5 mg) in 69% yield. Spectroscopic data are in accordance with the literature.[S11]

12. ^{1}H NMR (300 MHz, Chloroform-*d*) δ 8.49 (d, *J* = 1.9 Hz, 2H), 8.40 (d, *J* = 2.2 Hz, 1H), 2.87–2.74 (m, 2H), 1.68–1.54 (m, 2H), 0.98 (s, 9H). ^{13}C NMR (75 MHz, Chloroform-*d*) δ 158.8, 144.6, 143.9, 142.0, 44.0, 31.2, 30.7, 29.4. HRMS (EI) m/z: $[M+H]^+$ calculated for $C_{10}H_{16}N_2$ 165.1386, found 165.1380. GC-MS (m/z): 164.3 (40), 149.40, 107.1 (100), 942 (20).

Dimethyl 2-(adamantan-1-yl)succinate (13). From **1b** (0.375 mmol, 1.5 equiv, 0.075 M, 82 mg), **2a** (0.25 mmol, 1 equiv, 0.05 M, 31 µL), $[Acr\text{-}Mes]^+(BF_4)^-$ (0.025 mmol, 10 mol%, 10 mg) in a DCE /MeOH mixture 5:1 (5 mL). Purification by column chromatography (eluent: Cyclohexane/AcOEt 100/0 to 9/1) afforded **13** (white solid, mp 50–51.8 °C, 60.1 mg) in 87% yield. Spectroscopic data in accordance with the literature.[S4]

13. ^{1}H NMR (300 MHz, Chloroform-*d*) δ 3.69 (s, 3H), 3.65 (s, 3H), 2.83–2.70 (m, 1H), 2.53 (t, *J* = 2.8 Hz, 1H), 2.48 (t, *J* = 2.5 Hz, 1H), 1.98 (s, 3H), 1.74–1.58 (m, 10H), 1.46 (d, *J* = 12.5 Hz, 2H). ^{13}C NMR (75 MHz, Chloroform-*d*) δ 173.5, 52.4, 51.9, 51.4, 40.1, 36.9, 34.6, 31.1, 28.7.

Allyl 3-(adamantan-1-yl)-2-methylpropanoate (14). From **1b** (0.375 mmol, 1.5 equiv, 0.075 M, 82 mg), **2c** (0.25 mmol, 1 equiv, 0.05 M, 33 µL), $[Acr\text{-}Mes]^+(BF_4)^-$ (0.025 mmol, 10 mol%, 10 mg) in a DCE/MeOH mixture 5:1 (5 mL). Purification by column chromatography (eluent: Cyclohexane/AcOEt 100/0 to 4/1) gave **14** (pale yellow oil, 59.7 mg) in 91% yield.

14. ^{1}H NMR (300 MHz, acetone-*d*$_6$) δ 5.95 (ddt, *J* = 17.3, 10.8, 5.6 Hz, 1H), 5.33 (dq, *J* = 17.2, 1.7 Hz, 1H), 5.20 (dq, *J* = 10.4, 1.5 Hz, 1H), 4.55 (dq, *J* = 5.7, 1.4 Hz, 2H), 2.57 (ddp, *J* = 14.1, 7.1, 3.6 Hz, 1H), 1.91 (s, 4H), 1.75–1.63 (m, 9H), 1.48 (dq, *J* = 11.9, 2.6 Hz, 6H), 1.40 (d, *J* = 12.6 Hz, 2H), 1.12 (d, *J* = 7.0 Hz, 3H), 1.04 (dd, *J* = 14.1, 3.2 Hz, 1H). ^{13}C NMR (75 MHz, acetone-*d*$_6$) δ 177.4, 133.8, 117.9, 65.2, 49.3, 43.0, 37.7, 36.5, 34.9, 33.3, 20.8. HRMS (EI) m/z: $[M+H]^+$ calculated for $C_{17}H_{26}O_2$ 263.2006, found 263.2002.

3-(Adamantan-1-yl)-N,N-dimethylpropanamide (15). From **1b** (0.375 mmol, 1.5 equiv, 0.075 M, 82 mg), **2d** (0.25 mmol, 1 equiv, 0.05 M, 25 µL), $[Acr\text{-}Mes]^+(BF_4)^-$ (0.025 mmol, 10 mol%, 10 mg) in a DCE/MeOH mixture 5:1 (5 mL). Purification by column chromatography (eluent: Cyclohexane/AcOEt 100/0 to 9/1) afforded **15** (pale yellow oil, 40.8 mg) in 70% yield. Spectroscopic data in accordance with the literature.[S12]

15. ^{1}H NMR (300 MHz, Chloroform-*d*) δ 3.08-2.89 (m, 6H), 2.32–2.21 (m, 2H), 1.96 (s, 3H), 1.80–1.58 (m, 10H), 1.46–1.37 (m, 2H).^{13}C NMR (75 MHz, Chloroform-*d*) δ ^{13}C NMR (75 MHz, Chloroform-d) δ 174.2, 42.3, 39.4, 37.3, 37.1, 32.1, 28.8, 27.1.

2-((Adamantan-1-yl)(phenyl)methyl)malononitrile (16). From **1b** (0.375 mmol, 1.5 equiv, 0.075 M, 82 mg), **2e** (0.25 mmol, 1 equiv, 0.05 M, 38 mg), $[Acr\text{-}Mes]^+(BF_4)^-$ (0.025 mmol, 10 mol%, 10 mg) in a DCE/MeOH mixture 5:1 (5 mL). Purification by column chromatography (eluent: Cyclohexane/AcOEt 100/0 to 95/5) gave **16** (white solid, mp 167–169 °C, 59.4 mg) in 83% yield. Spectroscopic data in accordance with the literature.[S13]

16. ^{1}H NMR (300 MHz, Chloroform-*d*) δ ^{1}H NMR (300 MHz, DMSO-d6) δ 7.49-7.29 (m, 4H), 4.24 (d, *J* = 5.3 Hz, 1H), 2.80 (d, *J* = 5.4 Hz, 1H), 2.03 (s, 3H), 1.73–1.54 (m, 12H). ^{13}C NMR (75 MHz, Chloroform-*d*) δ 135.4, 129.8 128.8, 128.7, 113.6, 113.43, 58.2, 40.6, 36.7, 36.5, 28.5, 23.9.

3-((Adamantan-1-yl)methyl)bicyclo[2.2.1]heptan-2-one (17). From **1b** (0.375 mmol, 1.5 equiv., 0.075 M, 82 mg), **2g** (0.25 mmol, 1 equiv, 0.05 M, 28 µL), $[Acr\text{-}Mes]^+(BF_4)^-$ (0.025 mmol, 10 mol%, 10 mg) in a DCE/MeOH mixture 5:1 (5 mL). Purification by column chromatography (eluent: Cyclohexane/AcOEt 100/0 to 9/1) afforded **17** (white solid, mp 68.8–70.0 °C, 62 mg) in 86% yield

17. ^{1}H NMR (300 MHz, Chloroform-*d*) δ 2.63 (s, 1H), 2.59 (d, *J* = 5.1 Hz, 1H), 2.05 (dd, *J* = 8.8, 4.2 Hz, 1H), 1.95 (s, 3H), 1.74–1.56 (m, 10H), 1.49 (s, 6H), 1.42 (s, 3H), 1.04 (dd, *J* = 14.5, 9.1 Hz, 1H). ^{13}C NMR (75 MHz, Chloroform-*d*) δ 221.2, 50.1, 49.8, 42.9, 41.0, 40.1, 37.6, 37.2, 32.6, 28.8, 27.1, 25.5, 21.6. HRMS (EI) m/z: $[M+H]^+$ calculated for $C_{18}H_{26}O$ 259.2056, found 259.2045.

1-(2-(Phenylsulfonyl)ethyl)adamantane (18). From **1b** (0.375 mmol, 1.5 equiv, 0.075 M, 82 mg), **2h** (0.25 mmol, 1 equiv, 0.05 M, 42 mg), $[Acr\text{-}Mes]^+(BF_4)^-$ (0.025 mmol, 10 mol%, 10 mg) in a DCE/MeOH mixture 5:1 (5 mL). Purification by column chromatography (eluent: DCM/MeOH 100/0 to 95/5) gave **18** (pale yellow oil, 28.3 mg) 37% yield. Spectroscopic data in accordance with the literature.[S12]

18. ^{1}H NMR (300 MHz, Chloroform-*d*) δ 7.93–7.87 (m, 2H), 7.69–7.60 (m, 1H), 7.60–7.51 (m, 2H), 3.11–2.98 (m, 2H), 1.93 (s, 3H), 1.72–1.51 (m, 6H), 1.51–1.43 (m, 2H), 1.39 (s, 6H). ^{13}C NMR (75 MHz, Chloroform-*d*) δ 139.4, 133.7, 128.1, 51.4, 42.0, 36.9, 36.0, 32.0, 28.5.

2-(2-Adamantan-1-yl)ethyl)pyridine (19). From **1b** (0.375 mmol, 1.5 equiv, 0.075 M, 82 mg), **2i** (0.25 mmol, 1 equiv, 0.05 M, 26 µL), $[Acr\text{-}Mes]^{+}(BF_4)^{-}$ (0.025 mmol, 10 mol%, 10 mg) in a DCE/MeOH mixture 5:1 (5 mL). Purification by column chromatography (eluent: Cyclohexane/AcOEt 100/0 to 9/1) afforded **19** (colourless oil, 25.8 mg) in 43% yield. Spectroscopic data in accordance with the literature.[S10]

19. ^{1}H NMR (300 MHz, Chloroform-*d*) δ 8.51 (dt, *J* = 5.0, 1.3 Hz, 1H), 7.57 (td, *J* = 7.6, 1.9 Hz, 1H), 7.14 (d, *J* = 7.8 Hz, 1H), 7.07 (ddd, *J* = 7.6, 4.9, 1.2 Hz, 1H), 2.80–2.68 (m, 2H), 1.98 (s, 3H), 1.82–1.58 (m, 10H), 1.56–1.54 (m, 2H), 1.53–1.44 (m, 2H). ^{13}C NMR (75 MHz, Chloroform-*d*) δ 163.6, 149.3, 136.4, 122.8, 120.9, 44.9, 42.6, 37.4, 32.6, 32.00, 28.9.

2-(2-Adamantan-1-yl)ethyl)pyrazine (20). From **1b** (0.375 mmol, 1.5 equiv, 0.075 M, 82 mg), **2j** (0.25 mmol, 1 equiv, 0.05 M, 25 µL), $[Acr\text{-}Mes]^{+}(BF_4)^{-}$ (0.025 mmol, 10 mol%, 10 mg) in a DCE/MeOH mixture 5:1 (5 mL). Purification by column chromatography (eluent: Cyclohexane/AcOEt 100/0 to 9/1) gave **20** (pale yellow oil, 26.8 mg) in 44% yield.

20. ^{1}H NMR (300 MHz, chloroform-*d*) δ 8.48 (s, 2H), 8.40 (s, 1H), 2.86–2.70 (m, 2H), 2.07–1.92 (m, 3H), 1.68 (qd, *J* = 11.8, 5.9 Hz, 6H), 1.58–1.40 (m, 8H). ^{13}C NMR (75 MHz, chloroform-*d*) δ 158.8, 144.5, 143.6, 141.7, 44.1, 42.2, 37.0, 32.4, 28.9, 28.6. HRMS (EI) m/z: $[M+H]^{+}$ calculated for $C_{16}H_{22}N_2$ 243.1856, found 243.1851.

Dimethyl 2-(((*tert*-butoxycarbonyl)(methyl)amino)methyl)succinate (21). From **1c** (0.375 mmol, 1.5 equiv, 0.075 M, 85 mg), **2a** (0.25 mmol, 1 equiv, 0.05 M, 31 µL), $[Acr\text{-}Mes]^{+}(BF_4)^{-}$ (0.025 mmol, 10 mol%, 10 mg) in DCE (5 mL). Purification by column chromatography (eluent: Cyclohexane/AcOEt 100/0 to 9/1) afforded **21** (pale yellow oil, 68.1 mg) in 86% yield. Spectroscopic data in accordance with the literature.[S14]

21. ^{1}H NMR (300 MHz, Chloroform-*d*) δ 3.70 (s, 3H), 3.67 (s, 3H), 3.47 (d, *J* = 6.8 Hz, 2H), 3.15 (m, 1H), 2.84 (s, 3H), 2.72 (dd, *J* = 16.9, 8.5 Hz, 1H), 2.48 (dd, *J* = 16.8, 5.3 Hz, 1H), 1.45 (s, 9H). ^{13}C NMR (75 MHz, Chloroform-*d*) δ 173.8, 172.1, 79.9, 52.3, 51.9, 50.5, 40.6, 33.5, 28.5.

***tert*-Butyl (3,3-dicyano-2-phenylpropyl)(methyl)carbamate (22).** From **1c** (0.375 mmol, 1.5 equiv, 0.075 M, 85 mg), **2e** (0.25 mmol, 1 equiv, 0.05 M, 38 mg), $[Acr\text{-}Mes]^{+}(BF_4)^{-}$ (0.025 mmol, 10 mol%,

10 mg) in DCE (5 mL). Purification by column chromatography (eluent: Cyclohexane/AcOEt 100/0 to 7/3) gave **22** (pale yellow oil, 53.2 mg) in 72% yield. Spectroscopic data in accordance with the literature.[S15]

22. ^{1}H NMR (300 MHz, Chloroform-*d*) δ 7.48–7.39 (m, 3H), 7.38–7.31 (m, 2H), 4.14 (s, 1H), 3.99 (dd, *J* = 13.9, 8.2 Hz, 1H), 3.65–3.55 (m, 1H), 3.50 (dd, *J* = 13.9, 6.0 Hz, 1H), 2.81 (s, 3H), 1.48 (s, 9H). ^{13}C NMR (75 MHz, Chloroform-*d*) δ 135.1, 129.5, 129.4, 128.2, 112.2, 111.7, 81.1, 51.8, 45.6, 35.8, 28.5, 27.3.

***tert*-Butyl methyl(2-(3-oxobicyclo[2.2.1]heptan-2-yl)ethyl)carbamate (23).** From **1c** (0.375 mmol, 1.5 equiv, 0.075 M, 85 mg), **2g** (0.25 mmol, 1 equiv, 0.05 M, 28 µL), $[Acr\text{-}Mes]^{+}(BF_4)^{-}$ (0.025 mmol, 10 mol%, 10 mg) in DCE (5 mL). Purification by column chromatography (eluent: Cyclohexane/AcOEt 100/0 to 1/1) afforded **23** (pale yellow oil, 39 mg) in 54% yield.

23. ^{1}H NMR (300 MHz, Chloroform-*d*) 3.39–3.10 (m, 2H), 2.83 (s, 3H), 2.75–2.56 (m, 2H), 2.01–1.75 (m, 3H), 1.71–1.53 (m, 6H), 1.45 (s, 9H). ^{13}C NMR (75 MHz, Chloroform-*d*) δ 219.8, 155.8, 79.5, 51.3, 50.4, 38.5, 37.2, 34.1, 28.6, 27.0, 25.5, 21.3. HRMS (EI) m/z: $[M+Na]^{+}$ calculated for $C_{15}H_{25}NO_3Na$ 290.1727, found 290.1723.

***tert*-Butyl methyl(3-(pyrazin-2-yl)propyl)carbamate (24).** From **1c** (0.375 mmol, 1.5 equiv, 0.075 M, 85 mg), **2j** (0.25 mmol, 1 equiv, 0.05 M, 25 µL), $[Acr\text{-}Mes]^{+}(BF_4)^{-}$ (0.025 mmol, 10 mol%, 10 mg) in DCE (5 mL). Purification by column chromatography (eluent: Cyclohexane/AcOEt 100/0 to 1/1) gave **33** (pale yellow oil, 26.7 mg) in 43% yield.

24. ^{1}H NMR (300 MHz, Chloroform-*d*) δ 8.47 (dd, *J* = 5.1, 1.8 Hz, 2H), 8.40 (d, *J* = 2.5 Hz, 1H), 3.30 (s, 3H), 2.95–2.74 (m, 5H), 1.98 (qui, *J* = 7.4 Hz, 2H), 1.43 (s, 9H). ^{13}C NMR (75 MHz, Chloroform-*d*) δ 155.9, 144.7, 144.2, 142.4, 48.3, 34.2, 32.7, 28.6, 27.4, 27.3. HRMS (EI) m/z: $[M+H]^{+}$ calculated for $C_{13}H_{21}N_3O_2$ 252.1707, found 252.1711.

Dimethyl 2-(2,2-dimethyl-1,3-dioxolan-4-yl)succinate (25). From **1d** (0.375 mmol, 1.5 equiv, 0.075 M, 69 mg), **2a** (0.25 mmol, 1 equiv, 0.05 M, 31 µL), $[Acr\text{-}Mes]^{+}(BF_4)^{-}$ (0.025 mmol, 10 mol%, 10 mg) in DCE (5 mL). Purification by column chromatography (eluent: Cyclohexane/AcOEt 100/0 to 7/3) afforded **25** in 90% yield (mixture of diastereoisomers d.r. 1:1, pale yellow oil, 51.4 mg). Spectroscopic data in accordance with the literature.[S16]

25. (Mixture of diastereoisomers). ^{1}H NMR (300 MHz, Chloroform-*d*) δ 4.35 (q, *J* = 6.1 Hz, 1H), 4.29–4.06 (m, 2H), 4.03–3.94 (m, 2H), 3.84–3.75 (m, 1H), 3.71 (s, 4H), 3.68 (s, 4H), 3.25–3.13 (m, 1H), 2.85–2.72 (m, 2H), 2.51 (dd, *J* = 16.8, 4.7 Hz, 1H), 1.47 (s, 1H), 1.43–1.34 (m, 7H), 1.32 (s,

6H). ^{13}C NMR (75 MHz, Chloroform-*d*) δ 172.6, 172.2, 109.7, 75.3, 66.4, 52.3, 52.0, 44.4, 32.0, 26.4, 25.1.

2-((2,2-Dimethyl-1,3-dioxolan-4-yl)(phenyl)methyl)malononitrile (26). From **1d** (0.375 mmol, 1.5 equiv, 0.075 M, 69 mg), **1e** (0.25 mmol, 1 equiv, 0.05 M, 38 mg), $[Acr\text{-}Mes]^+(BF_4)^-$ (0.025 mmol, 10 mol%, 10 mg) in DCE (5 mL). Purification by column chromatography (eluent: Cyclohexane/AcOEt 100/0 to 95/5) gave **26** in 65% yield (mixture of diastereoisomers d.r. 1:1, pale yellow solid, 97–99.9 °C, 41 mg).

26. ^1H NMR (300 MHz, Chloroform-*d*) δ 7.49–7.30 (m, 5H), 4.68–4.58 (m, 1H), 4.48 (d, *J* = 4.1 Hz, 1H), 3.94 (dd, *J* = 8.9, 6.2 Hz, 1H), 3.56 (dd, *J* = 9.0, 5.2 Hz, 1H), 3.16 (dd, *J* = 10.5, 4.1 Hz, 1H), 1.50 (s, 3H), 1.40 (s, 3H). ^{13}C NMR (75 MHz, Chloroform-*d*) δ 133.4, 129.8, 129.7, 128.6, 111.1, 77.6, 77.2, 76.7, 75.0, 67.9, 50.9, 27.3, 27.2, 25.2. HRMS (EI) m/z: $[M+Na]^+$ calculated for $C_{15}H_{16}N_2O_2Na$ 279.1104, found 279.1106.

3-((2,2-Dimethyl-1,3-dioxolan-4-yl)methyl)bicyclo[2.2.1]heptan-2-one (27). From **1d** (0.5 mmol, 2 equiv, 0.1 M, 92 mg), **2g** (0.25 mmol, 1 equiv, 0.05 M, 29 µL), $[Acr\text{-}Mes]^+(BF_4)^-$ (0.025 mmol, 10 mol%, 10 mg) in DCE (5 mL). Purification by column chromatography (eluent: Cyclohexane/AcOEt 100/0 to 1/1) afforded **27** in 74% yield (mixture of diastereoisomers d.r. 1:1, colourless oil, 41 mg).

27. (Mixture of diastereoisomers). ^1H NMR (300 MHz, Chloroform-*d*) δ 4.29–4.10 (m, 1H), 4.11–3.99 (m, 1H), 3.60–3.48 (m, 1H), 2.72–2.65 (m, 1H), 2.61 (t, *J* = 5.1 Hz, 1H), 2.25-2.15 (m, 1H), 2.02–1.43 (m, 8H), 1.39 (d, *J* = 2.8 Hz, 4H), 1.34 (s, 4H). ^{13}C NMR (75 MHz, Chloroform-*d*) δ 219.5, 109.1, 109.0, 75.6, 74.2, 69.7, 69.5, 51.5, 50.6, 50.4, 39.8, 38.6, 37.3, 31.0, 30.2, 27.1, 25.9, 25.8, 25.6, 25.5, 21.6, 21.5. HRMS (EI) m/z: $[M+H]^+$ calculated for $C_{13}H_{20}O_3$ 247.1305, found 247.1306.

2,2-Dimethyl-4-(2-(phenylsulfonyl)ethyl)-1,3-dioxolane (28). From **1d** (0.5 mmol, 2 equiv, 0.1 M, 92 mg), **2h** (0.25 mmol, 1 equiv, 0.05 M, 42 mg), $[Acr\text{-}Mes]^+(BF_4)^-$ (0.025 mmol, 10 mol%, 10 mg) in DCE (5 mL). Purification by column chromatography (eluent: Cyclohexane/AcOEt 100/0 to 2/3) gave **28** (pale yellow oil, 55 mg) in 82% yield. Spectroscopic data are in accordance with the literature.[S17]

28. ^1H NMR (300 MHz, Chloroform-*d*) δ 7.91 (d, *J* = 7.9, Hz, 2H), 7.74–7.59 (m, 1H), 7.62–7.48 (m, 2H), 4.23–4.08 (m, 1H), 4.03 (dd, *J* = 8.2, 6.0 Hz, 1H), 3.54 (dd, *J* = 8.2, 6.1 Hz, 1H), 3.29 (ddd, *J* = 14.3, 10.7, 5.2 Hz, 1H), 3.15 (ddd, *J* = 14.1, 10.7, 5.4 Hz, 1H), 2.07–1.95 (m, 1H), 1.95–1.83 (m, 1H), 1.32 (s, 3H), 1.28 (s, 3H). ^{13}C NMR (75 MHz, chloroform-*d*) δ 139.2, 133.9, 129.5, 128.1, 109.6, 73.9, 68.9, 52.9, 27.1, 26.9, 25.5.

2. Tables and Figures.

Table S1. Optimization of the Photoredox Catalyzed Synthesis of 3.

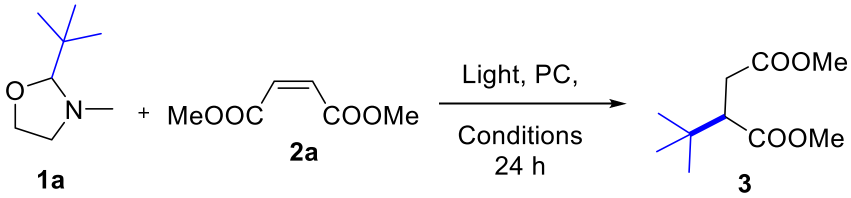

Entry	Conditions	3 (% Yield)
1	**2a** (0.05 M), **1a** (1.5 equiv), $Ir(ppy)_3$ (5 mol%), Ar, DCE, 405 nm,	-
2	**2a** (0.05 M), **1a** (1.5 equiv), $Ir(ppy)_3$ (5 mol%), air, DCE, 405 nm,	11
3	**2a** (0.05 M), **1a** (1.5 equiv), $Ru(bpy)_3Cl_2$ (5 mol%), air, DCE, 405 nm	5
4	**2a** (0.05 M), **1a** (1.5 equiv), $Ph_3Pyrylium^+$ BF_4^- (10 mol%), air, DCE, 405 nm	9
5	**2a** (0.05 M), **1a** (1.5 equiv), 4CzIPN (10 mol%), air, DCE, 405 nm	24
6	**2a** (0.05 M), **1a** (1.5 equiv), 4CzIPN (10 mol%), Ar, DCE, 405 nm	34
**7**	**2a (0.05 M), 1a (1.5 equiv), Acr-Mes$^+$ BF_4^- (10 mol%), air, DCE, 405 nm**	**88**
8	**2a** (0.05 M), **1a** (1.3 equiv), Acr-Mes$^+$ BF_4^- (10 mol%), air, DCE, 405 nm	66
9	**2a** (0.05 M), **1a** (1.5 equiv), Acr-Mes$^+$ BF_4^- (5 mol%), air, DCE, 405 nm	38
10	**2a** (0.05 M), **1a** (1.5 equiv), Acr-Mes$^+$ BF_4^- (10 mol%), air, DCM, 405 nm	52
11	**2a** (0.05 M), **1a** (1.5 equiv), Acr-Mes$^+$ BF_4^- (10 mol%), air, $CHCl_3$, 405 nm	10
12	**2a** (0.05 M), **1a** (1.5 equiv), Acr-Mes$^+$ BF_4^- (10 mol%), air, MeOH, 405 nm	5
13	**2a** (0.05 M), **1a** (1.5 equiv), Acr-Mes$^+$ BF_4^- (10 mol%), air, MeCN, 405 nm	-
14	**2a** (0.05 M), **1a** (1.5 equiv), Acr-Mes$^+$ BF_4^- (10 mol%), air, MeCN, no light	-
15	**2a** (0.05 M), **1a** (1.5 equiv), Acr-Mes$^+$ BF_4^- (10 mol%), air, DCE, TEMPO (1 equiv) 405 nm	13

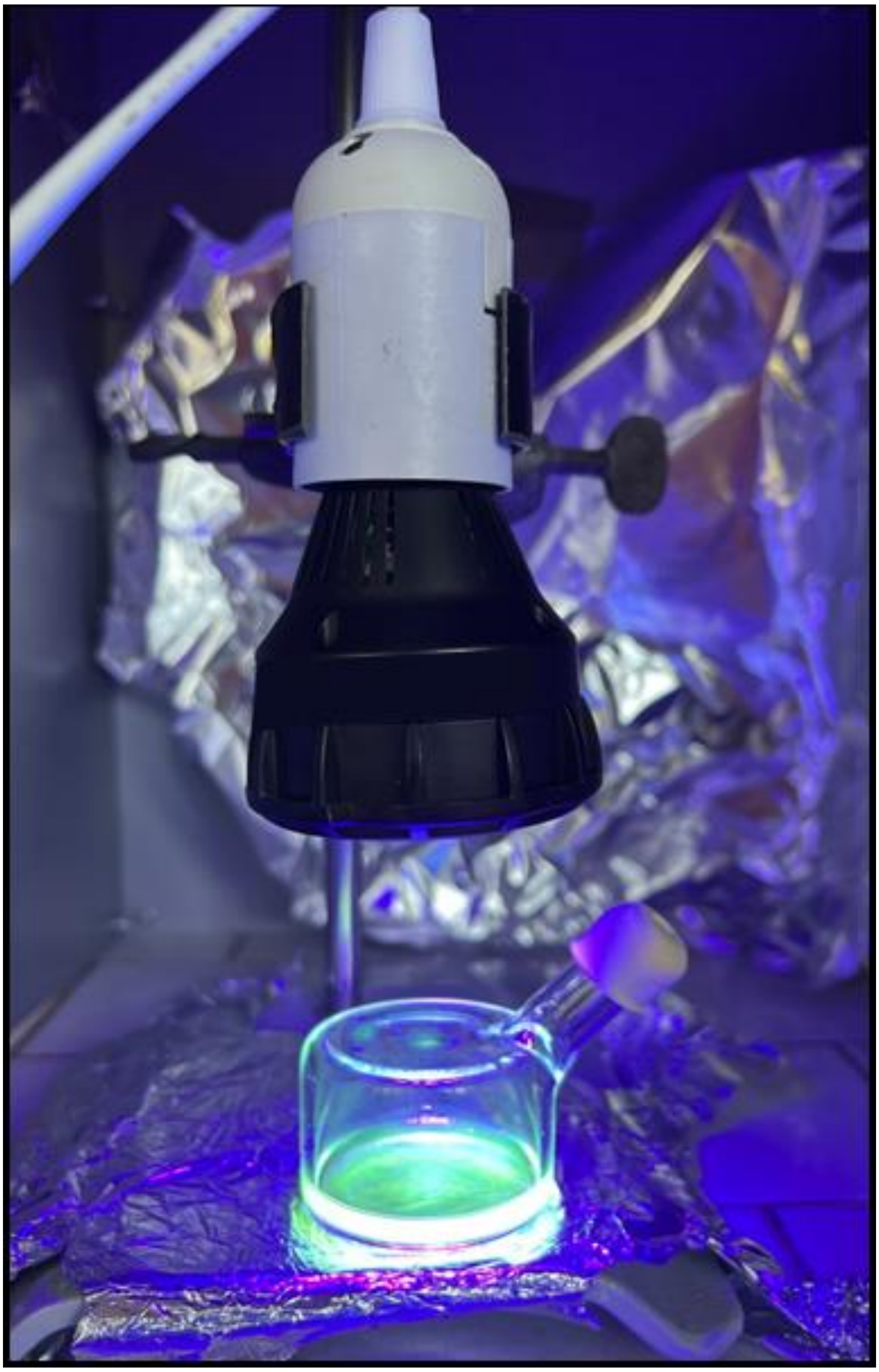

Figure S1. Irradiation system employed in the present work.

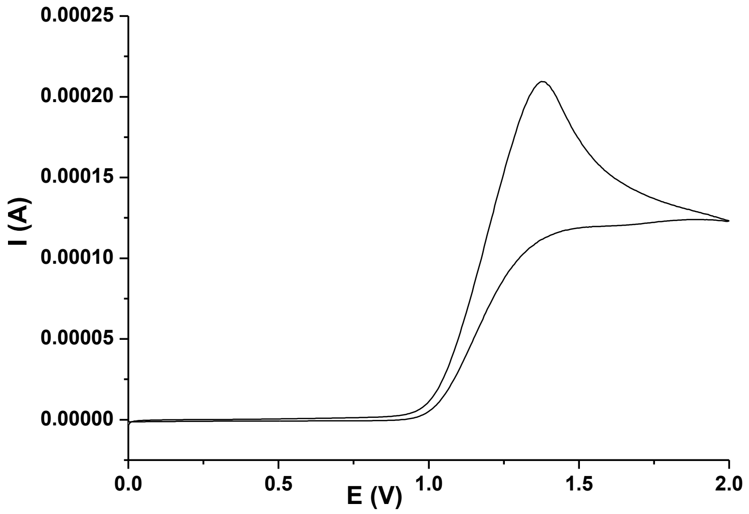

Figure S2. Cyclic voltammetry of **1a**

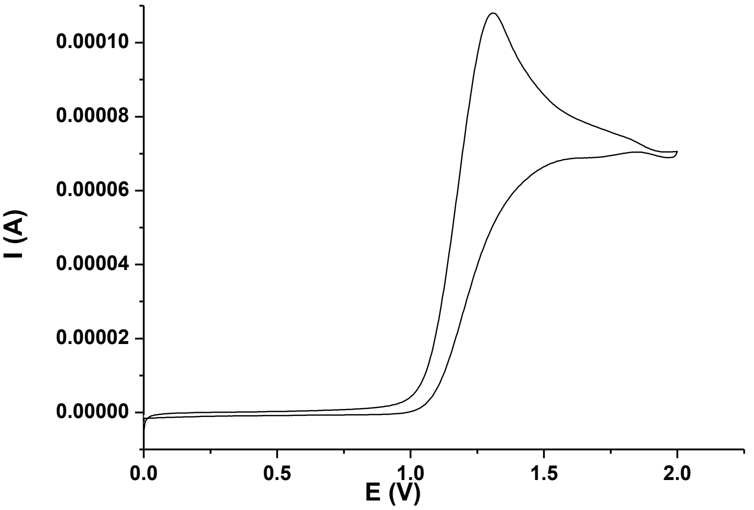

Figure S3. Cyclic voltammetry of **1b**

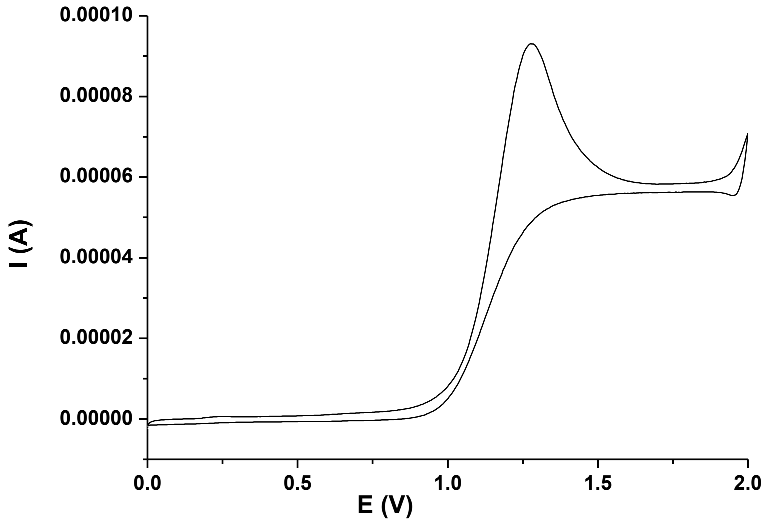

Figure S4. Cyclic voltammetry of **1c**

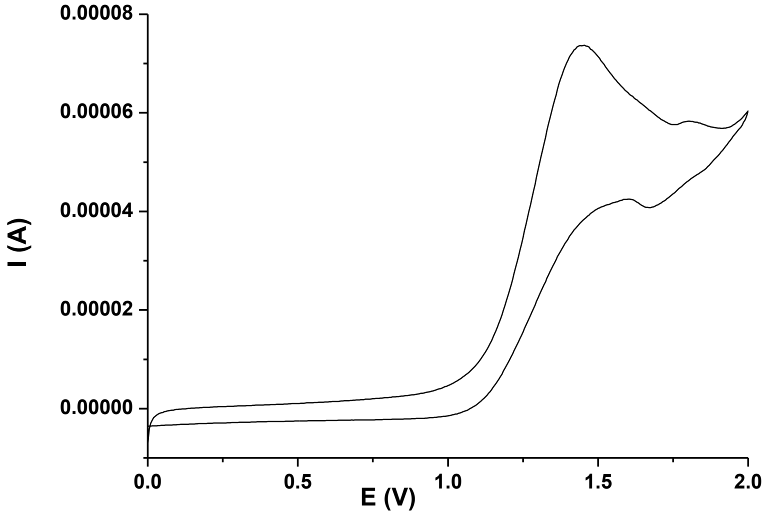

Figure S5. Cyclic voltammetry of **1d**

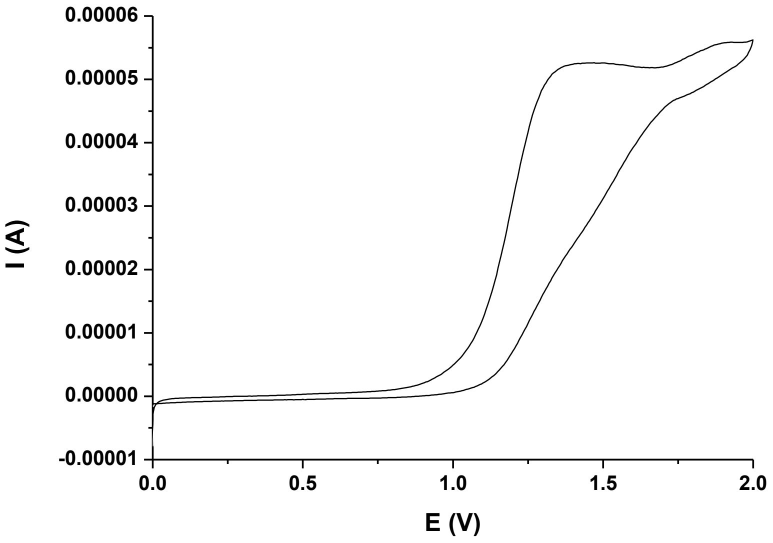

Figure S6. Cyclic voltammetry of **1e**

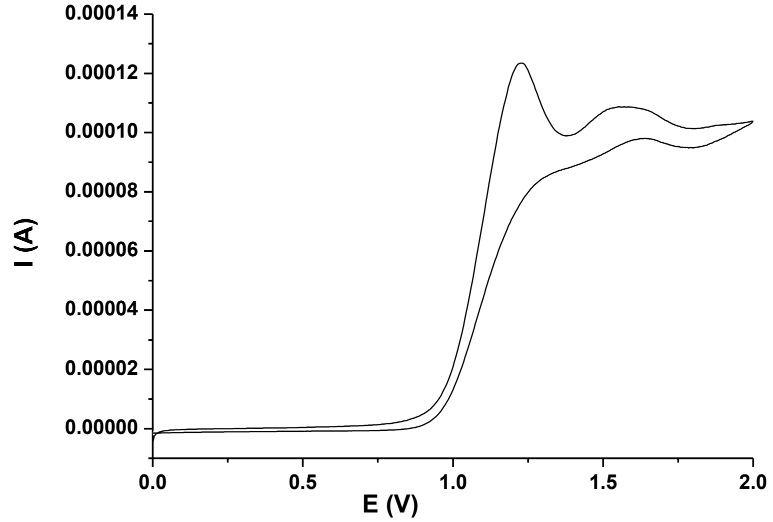

Figure S7. Cyclic voltammetry of **1f**

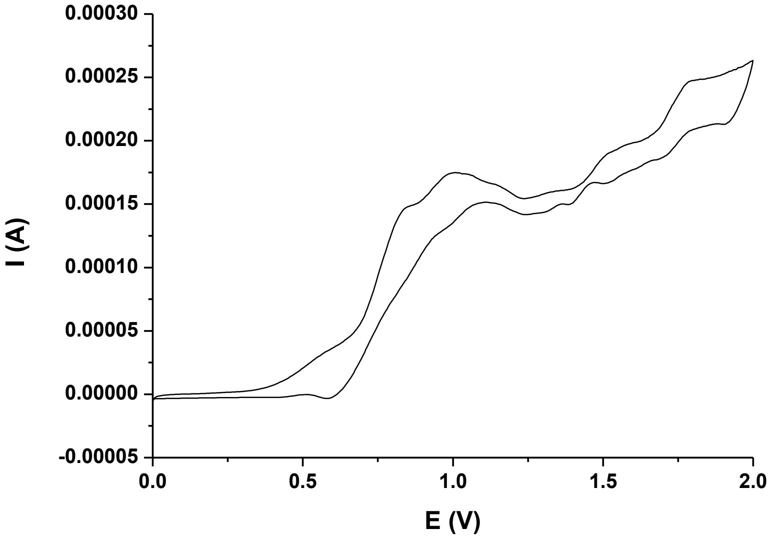

Figure S8. Cyclic voltammetry of **1g**

3. Deuteration experiments.

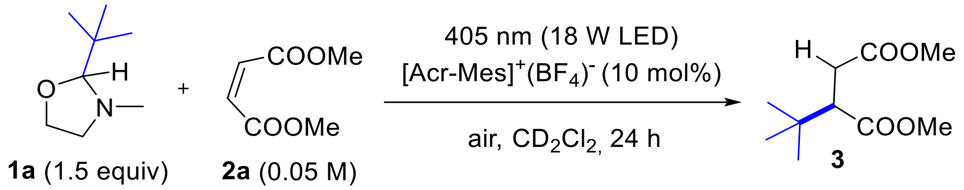

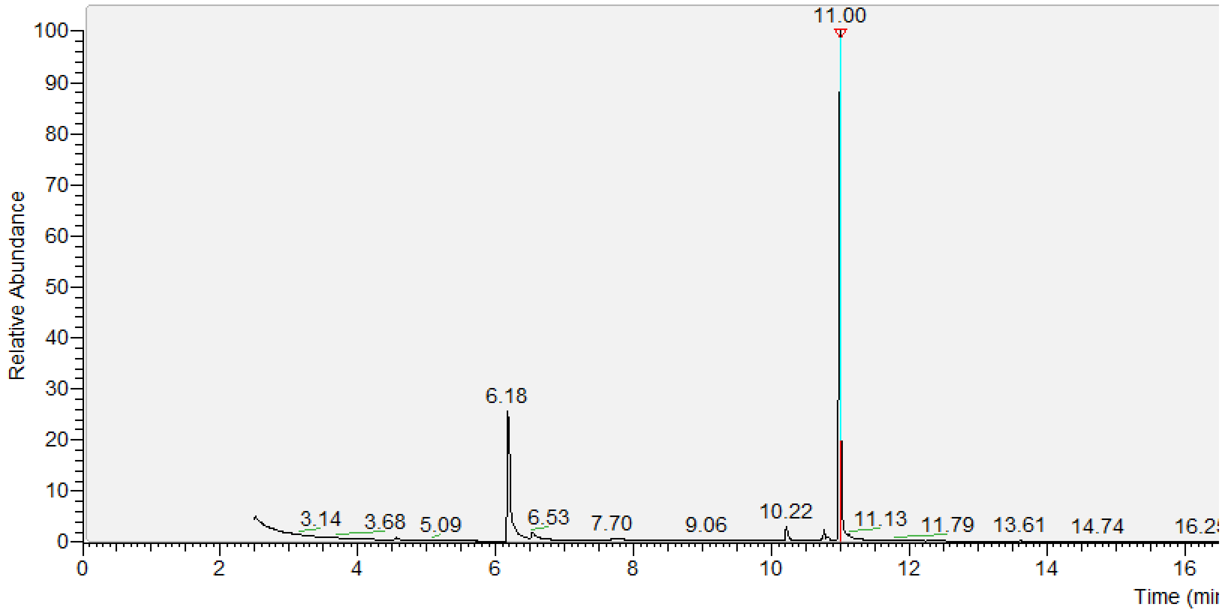

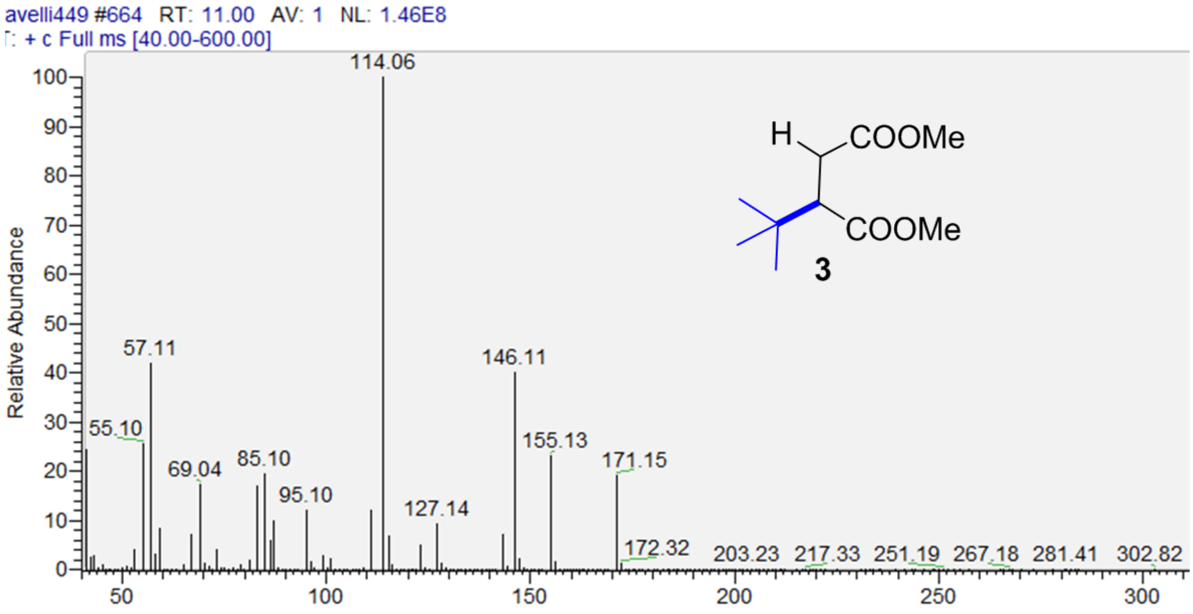

Figure S9. GC-MS fragmentation spectrum of compound **3**. Irradiation carried out in CD_2Cl_2

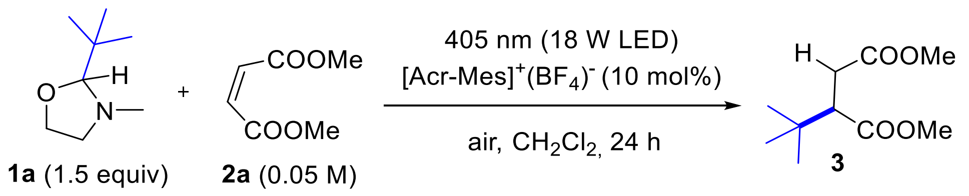

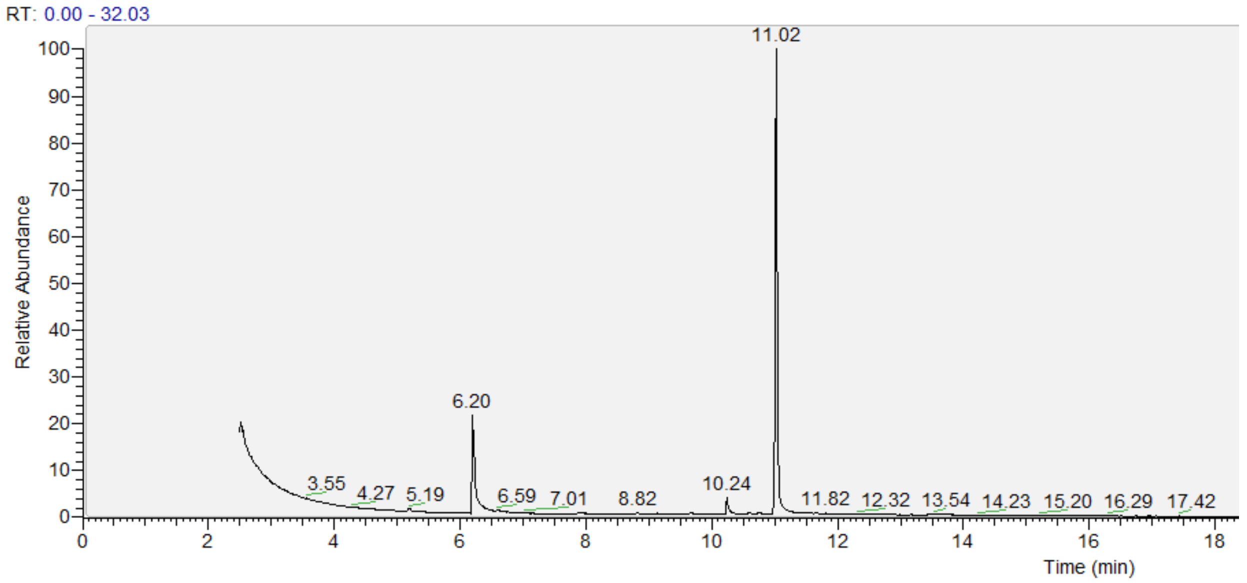

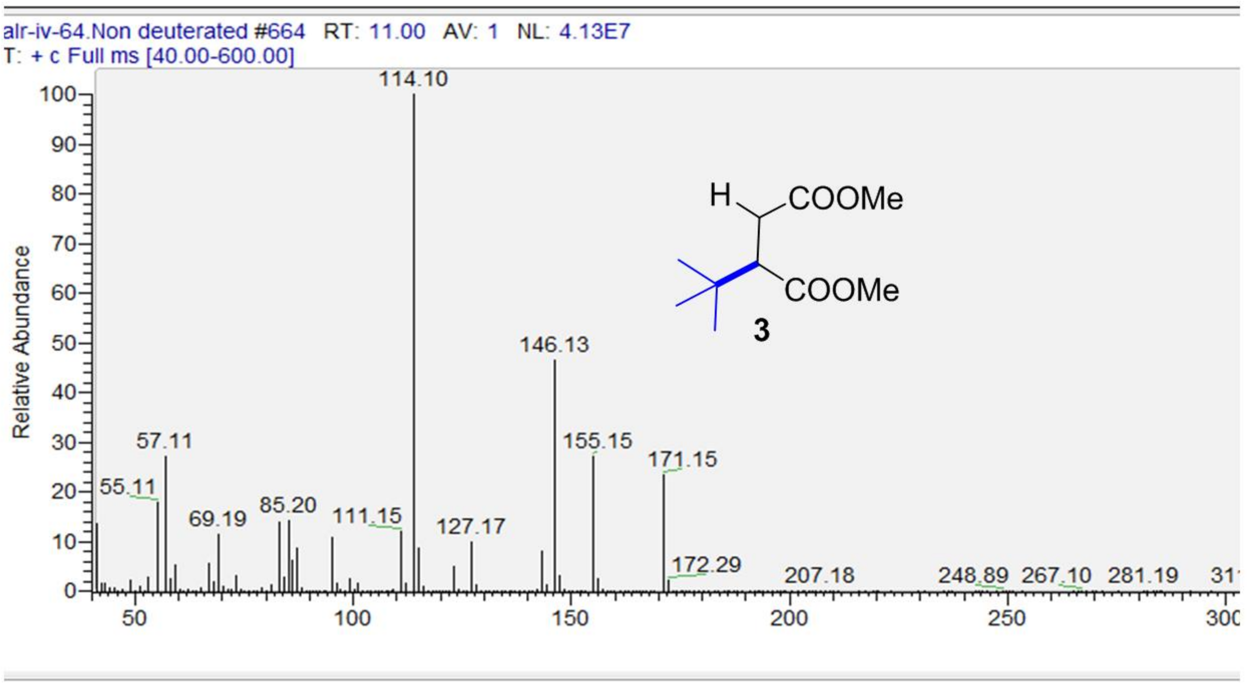

Figure S10. GC-MS fragmentation spectrum of compound **3**. Irradiation carried out in CH_2Cl_2

4. References

(S1) Takeuchi, K.; Kitagawa, I.; Akiyama, F.; Shibata, T.; Kato, M.; Okamoto, K. Ring-Expansion of Bridgehead Aldehydes with 1-Adamantanecarbonyl Cation or Benzoyl Trifluoromethanesulfonate: A New Route to Bicyclic and Tricyclic 1,2-Diols. *Synthesis* **1987**, *1987*, 612–615.

(S2) Bureau, R.; Mortier, J.; Joucla, M. Formation of azomethine ylids by thermolysis of oxazolidines. Study of the reaction in solution and in the gaseous phase. *Bull. Soc. Chim. Fr.* **1993**, *130*, 584–596.

(S3) Waller, R. W.; Diorazio, L. J.; Taylor, B. A.; Motherwell, W. B.; Sheppard, T. D.; Isocyanide based multicomponent reactions of oxazolidines and related systems. *Tetrahedron,* **2010**, *66* 6496–6507.

(S4) Rohe, S.; Morris, A. O.; McCallum, T.; Barriault, L. Hydrogen Atom Transfer Reactions via Photoredox Catalyzed Chlorine Atom Generation. *Angew. Chem. Int. Ed.* **2018**, *57*, 15664–15669.

(S5) Zhou, X.; Zhang, G.; Gao, B.; Huang**,** H. Palladium-Catalyzed Hydrocarbonylative C–N Coupling of Alkenes with Amides. *Org. Lett.* **2018**, *20*, 2208–2212.

(S6) Uchikura, T.; Moriyama, K.; Toda, M.; Mouri, T.; Ibáñez, I.; Akiyama, T. Benzothiazolines as radical transfer reagents: hydroalkylation and hydroacylation of alkenes by radical generation under photoirradiation conditions. *Chem. Commun*. **2019**, *55*, 11171–11174.

(S7) Pitre, S. P.; Allred, T. K.; Overman, L. E. Lewis Acid Activation of Fragment-Coupling Reactions of Tertiary Carbon Radicals Promoted by Visible-Light Irradiation of EDA Complexes. *Org. Lett.* **2021**, *23*, 1103–1106.

(S8) Petrier, C.; De Souza Barbosa, J.; Dupuy, C.; Luche, J. L. Ultrasound in organic synthesis. 7. Preparation of organozinc reagents and their nickel-catalyzed reactions with α,β-unsaturated carbonyl compounds, *J. Org. Chem.* **1985**, *50*, 5761–5765.

(S9) Xue, F.; Wang, F.; Liu, J.; Di, J.; Liao, Q.; Lu, H.; Zhu, M.; He, L.; He, H.; Zhang, D.; Song, H.; Liu, X.Y.; Qin. Y. A Desulfurative Strategy for the Generation of Alkyl Radicals Enabled by Visible-Light Photoredox Catalysis. *Angew. Chem. Int. Ed.* **2019**, *57*, 6667–6671.

(S10) Pang, H.; Wang, Y.; Gallou, F.; Lipshutz, B. H. Fe-catalyzed reductive couplings of terminal (hetero) aryl alkenes and alkyl halides under aqueous micellar conditions. *J. Am. Chem. Soc.* **2019**, *141*, 17117–17124.

(S11) Braquet P.; Pirotzky E.; Godfroid J.-J.; Heymans F. 2-substituted N,N'-ditrimethoxybenzoyl piperazines and therapeutic compositions containing them. *US Pat.* **1991**, US5019576 A

(S12) Sato, R.; Okamoto, R.; Ishizuka, T.; Nakayama, A.; Karanjit, S.; Namba, K. Microwave-assisted Tertiary Carbon Radical Reaction for Construction of Quaternary Carbon Center. *Chem. Lett*. **2019**, *48*, 414–417.

(S13) Xu, Q. H.; Wei, L. P.; Xiao, B. Alkyl-GeMe3: Neutral Metalloid Radical Precursors upon Visible-Light Photocatalysis. *Angew. Chem. Int. Ed.* **2022**, *61*, e202115592.

(S14) Schweitzer-Chaput, B.; Horwitz, M. A.; Beato, E. P.; Melchiorre, P. Photochemical generation of radicals from alkyl electrophiles using a nucleophilic organic catalyst. *Nat. Chem.* **2019**, *11*, 129–135.

(S15) Ohmatsu, K.; Suzuki, R.; Suzuki, R.; Furukawa, Y.; Sato, M.; Ooi, T. Zwitterionic 1, 2, 3-triazolium amidate as a catalyst for photoinduced hydrogen-atom transfer radical alkylation. *ACS Catal.* **2020**, *10*, 4, 2627–2632.

(S16) Hayakawaa, M.; Shimizua, R.; Omoria, H.; Shirotaa, H.; Uchidaa, K.; Mashimoa, H.; Ryuusei, H. X.; Seiya, Y.; Yoshiki, N.; Chuanxiang, W.; Tadashi, L.; Ouchia A. Photochemical addition of cyclic ethers/acetals to olefins using tBuOOtBu: Synthesis of masked ketones/aldehydes and diols. *Tetrahedron* **2020**, *76*, 131557.

(S17) Raviola, C.; Ravelli, D. Efficiency and Selectivity Aspects in the C–H Functionalization of Aliphatic Oxygen Heterocycles by Photocatalytic Hydrogen Atom Transfer. *Synlett* **2019**, *30*, 803–808.

5. Copy of ^{1}H and ^{13}C NMR spectra.

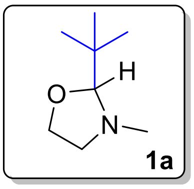

^{1}H NMR (300 MHz, acetone-d_6)

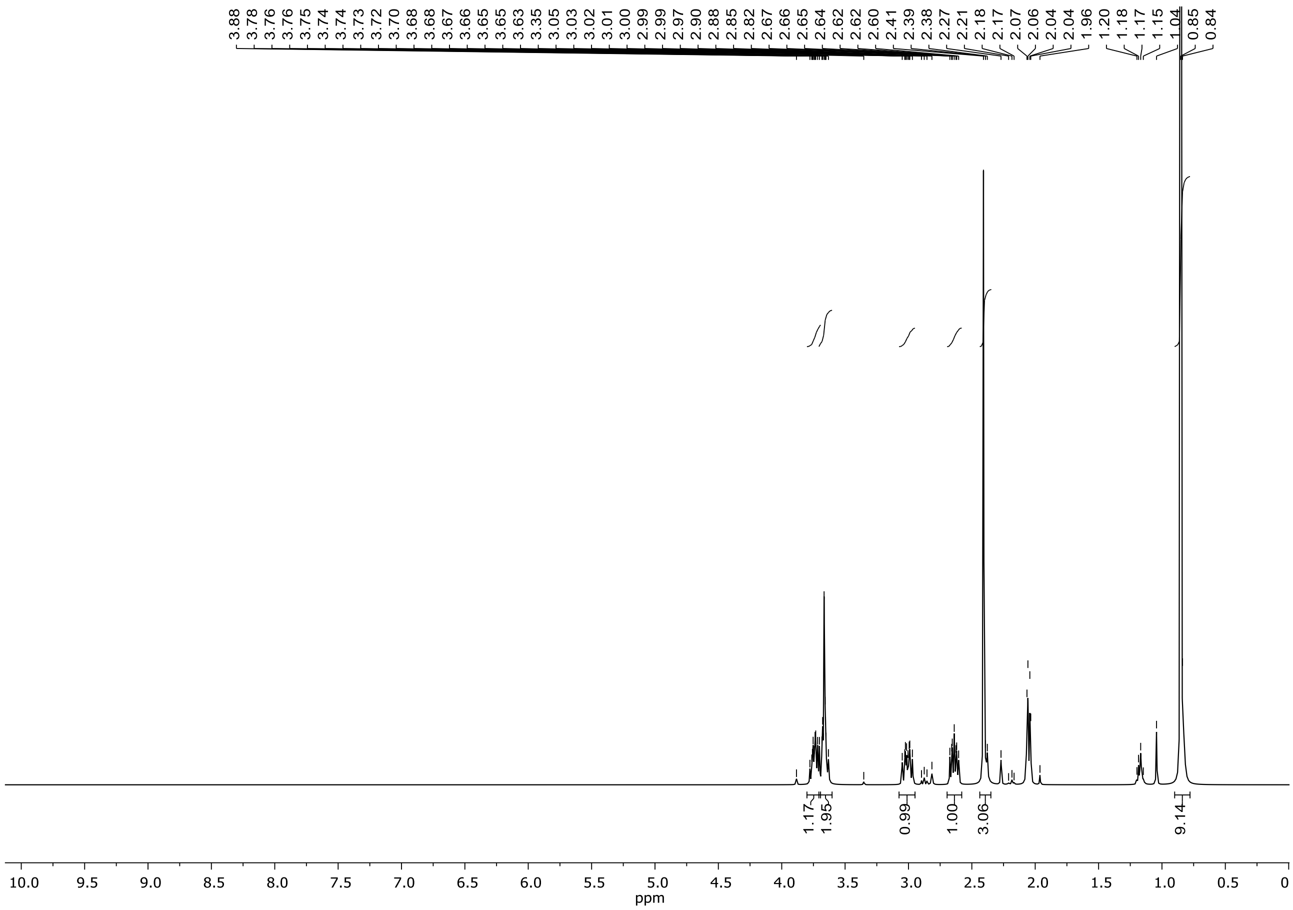

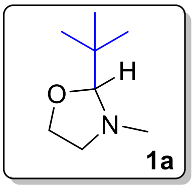

^{13}C NMR (75 MHz, acetone-d_6)

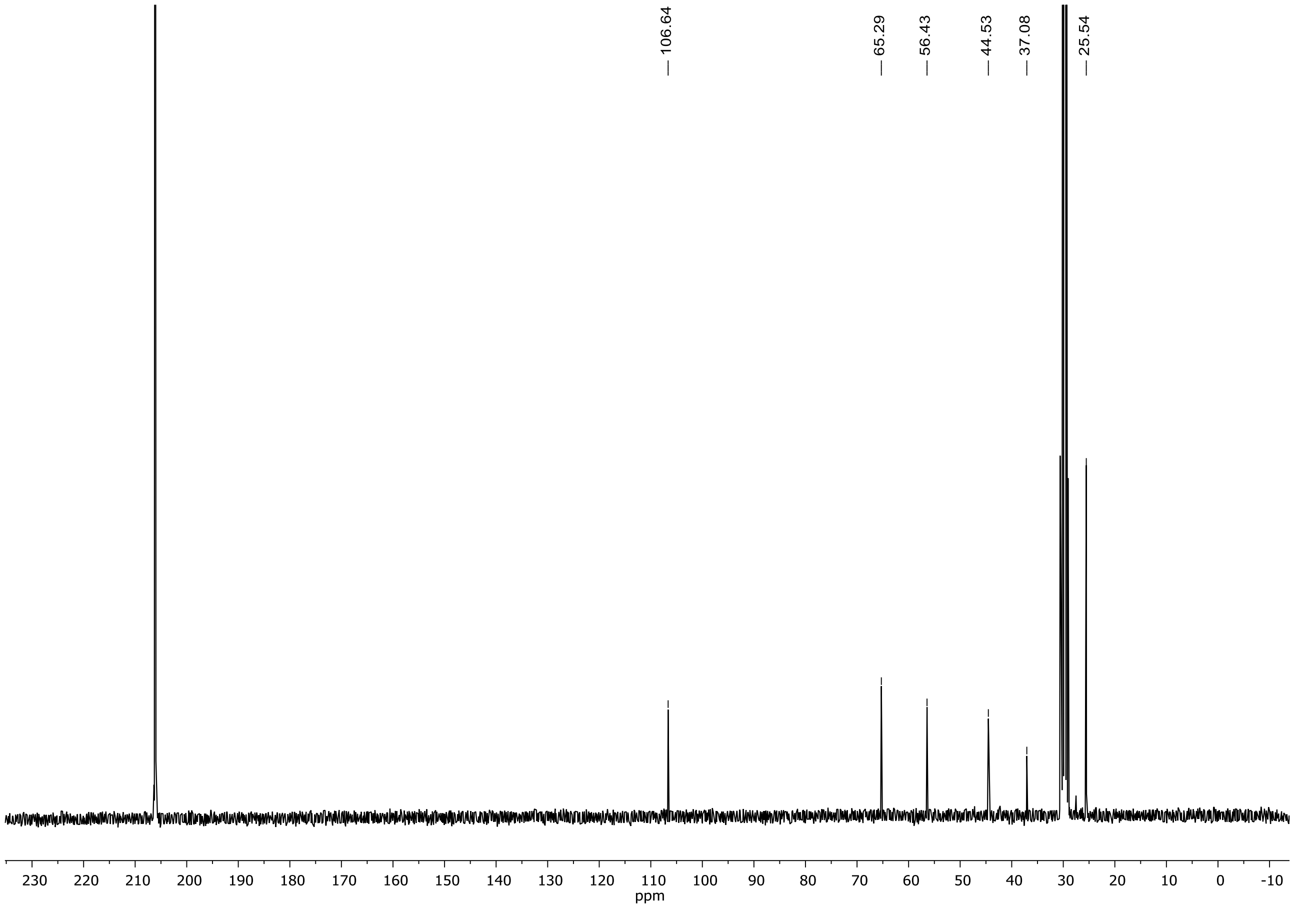

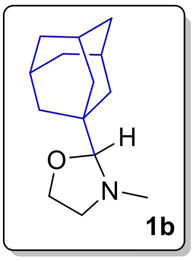

1b

^{1}H NMR (300 MHz, acetone-d_6)

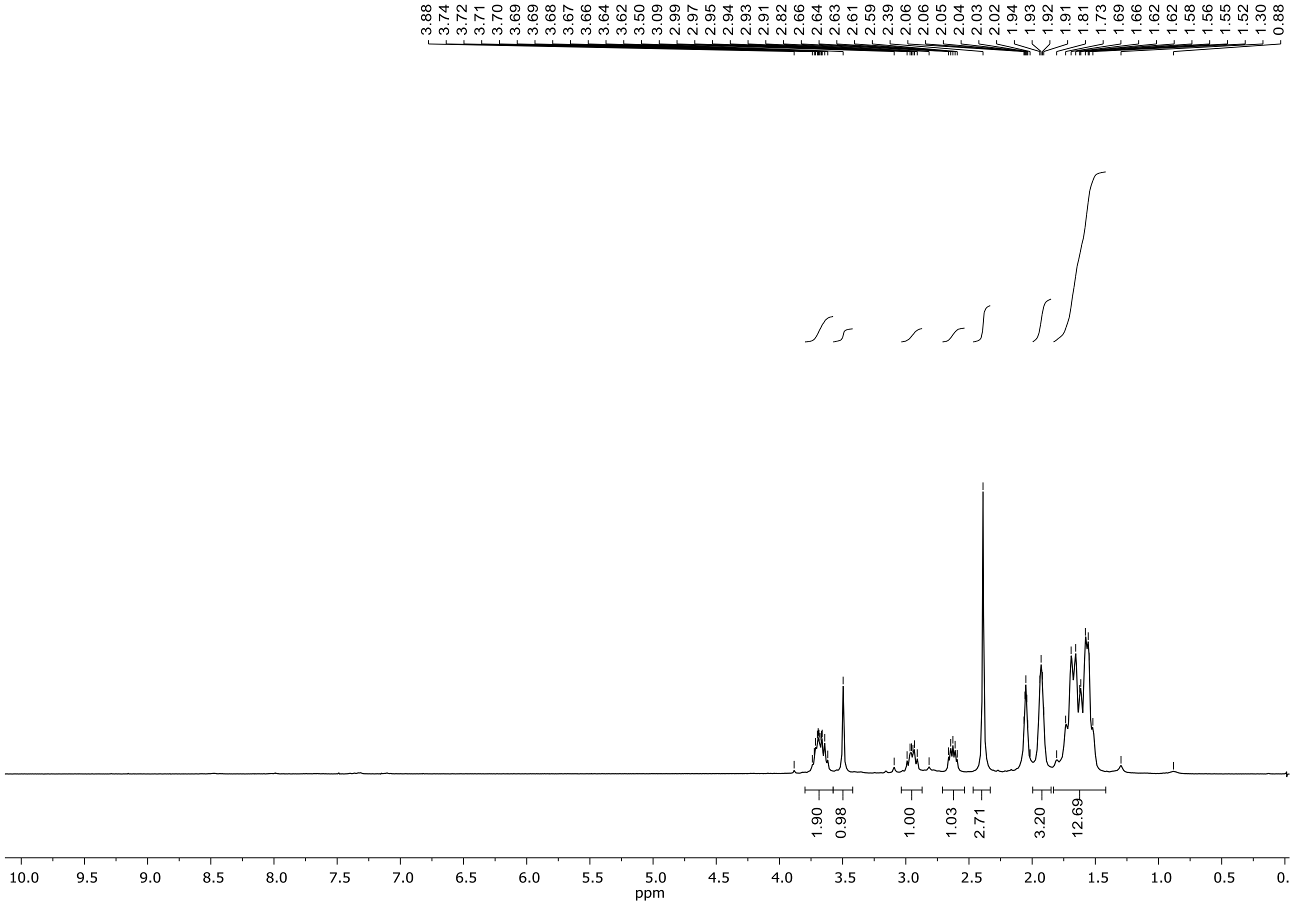

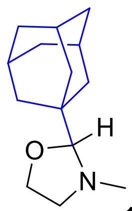

1b

^{13}C NMR (75 MHz, acetone-d_6)

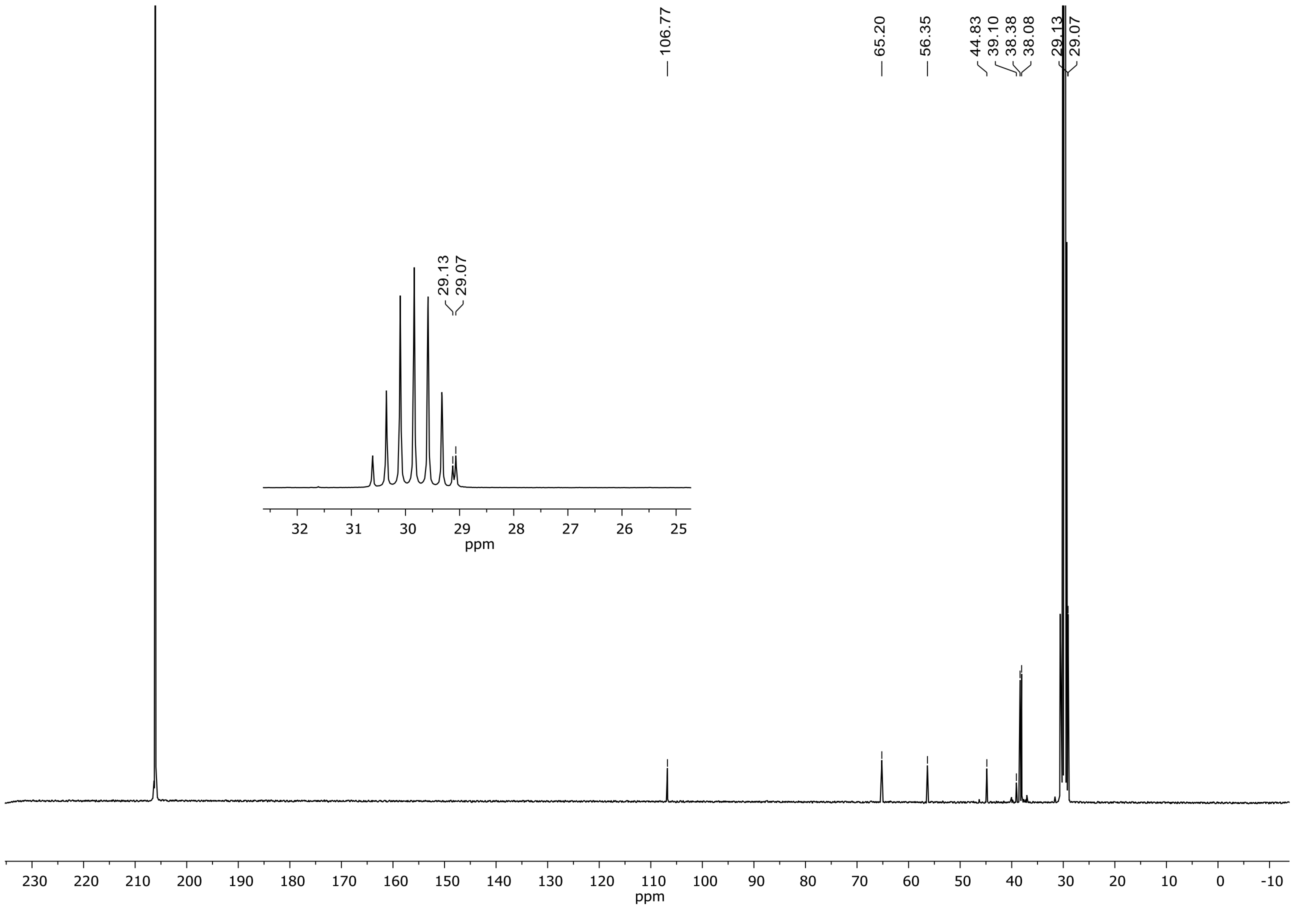

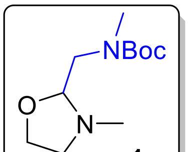

1c

^{1}H NMR (300 MHz, acetone-d_6)

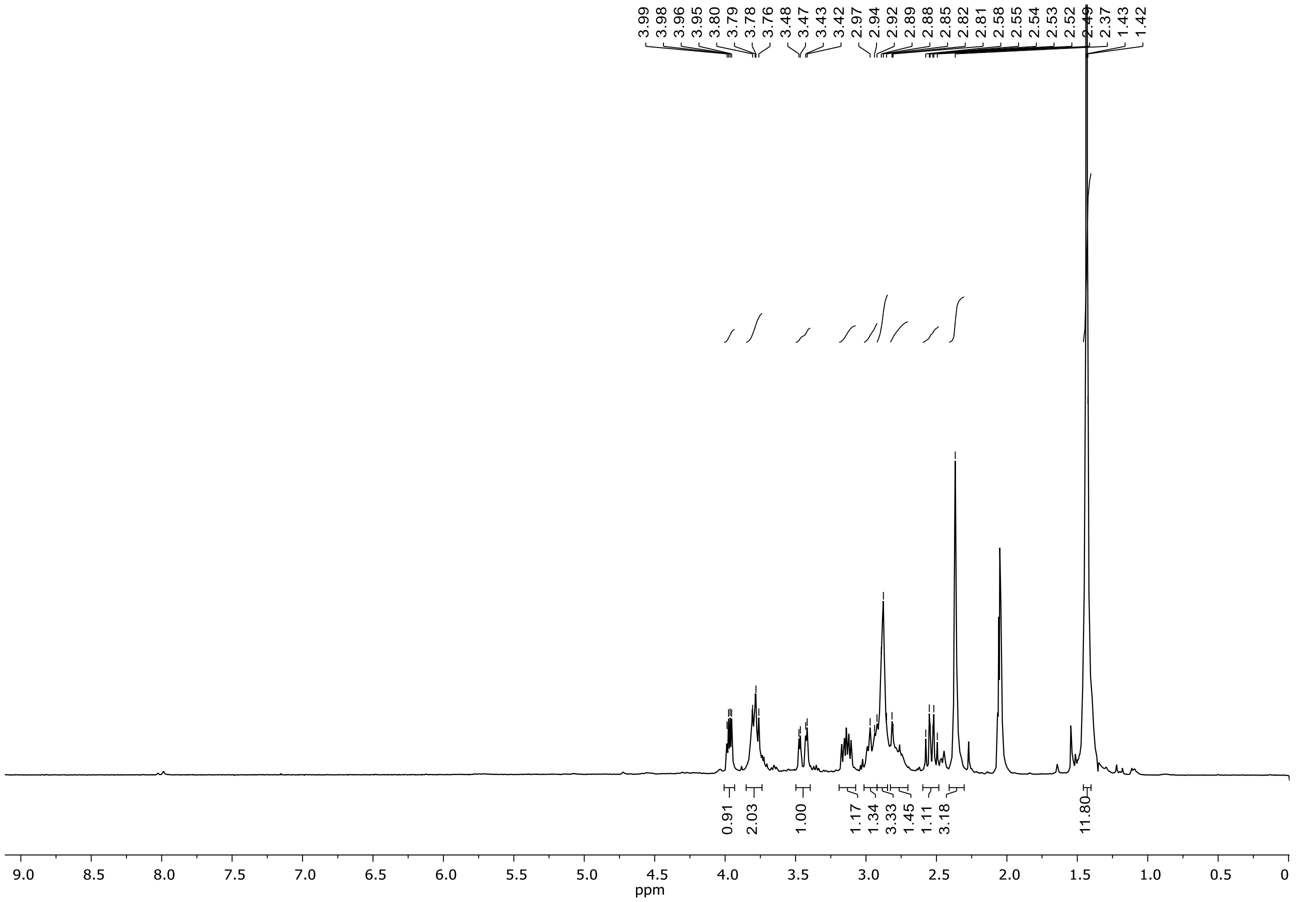

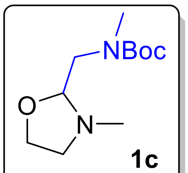

1c

^{13}C NMR (75 MHz, acetone-d_6)

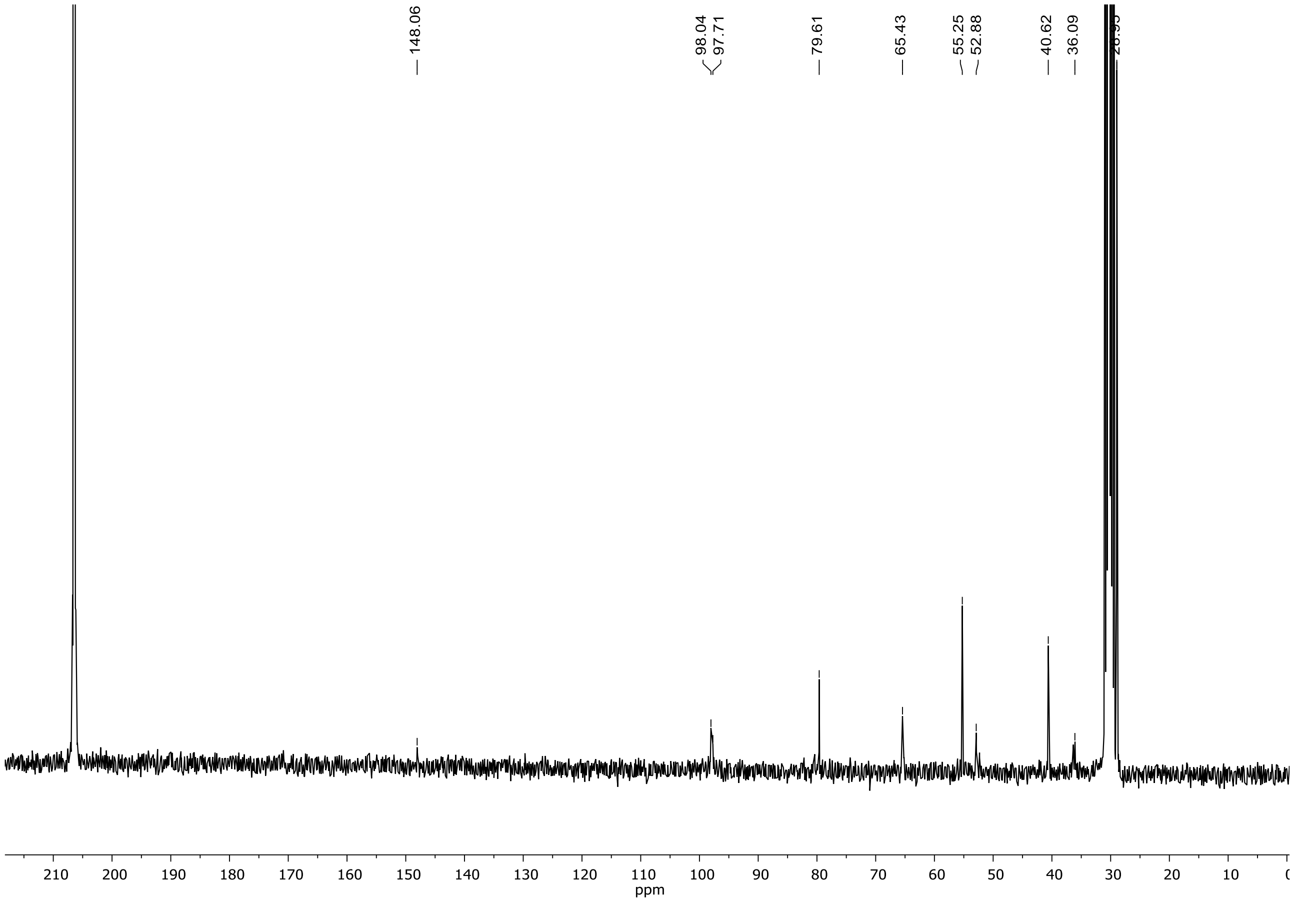

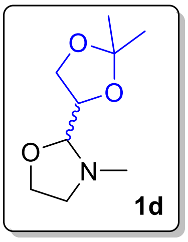

^{1}H NMR (300 MHz, acetone-d_6)

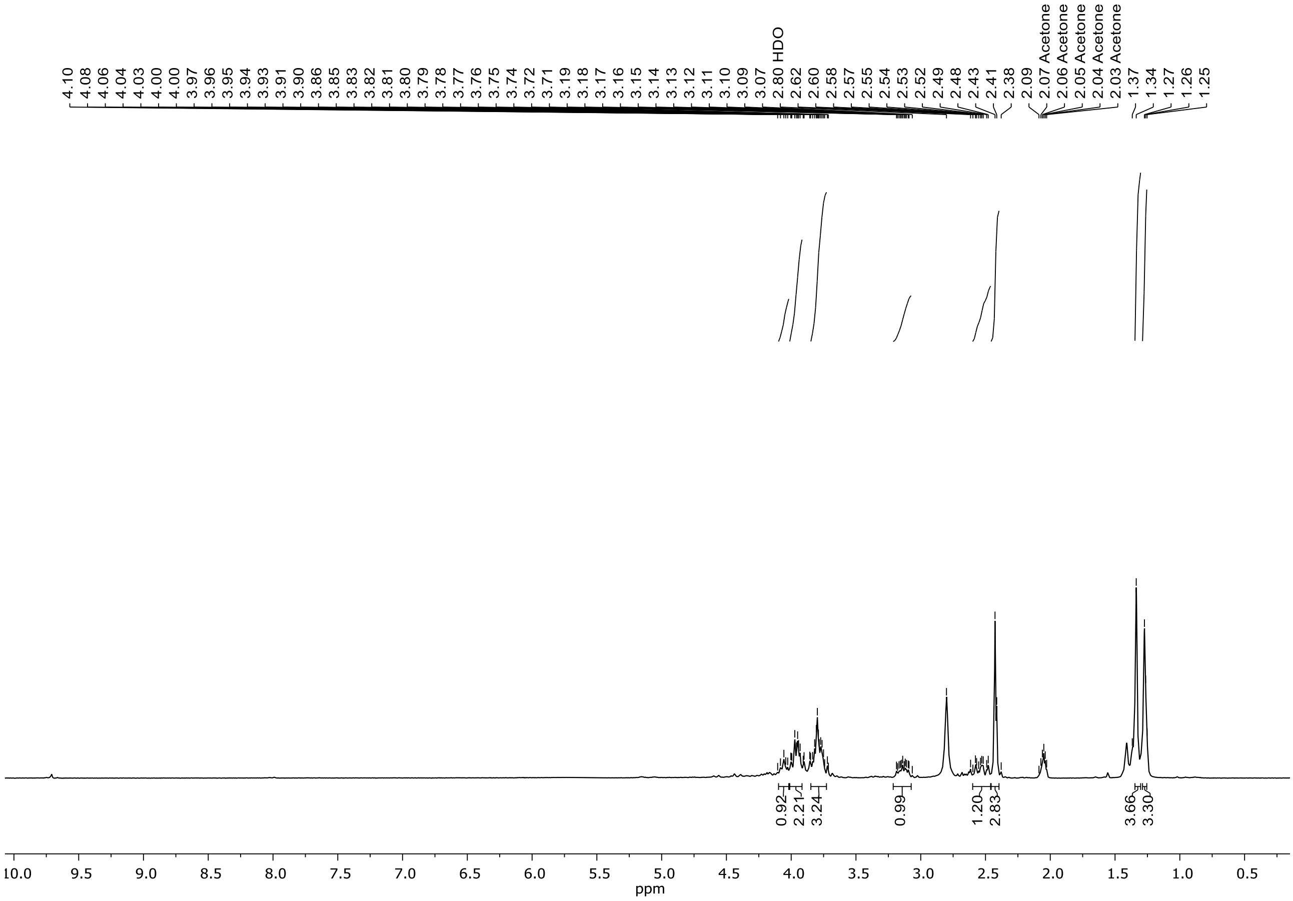

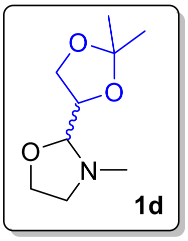

^{13}C NMR (75 MHz, acetone-d_6)

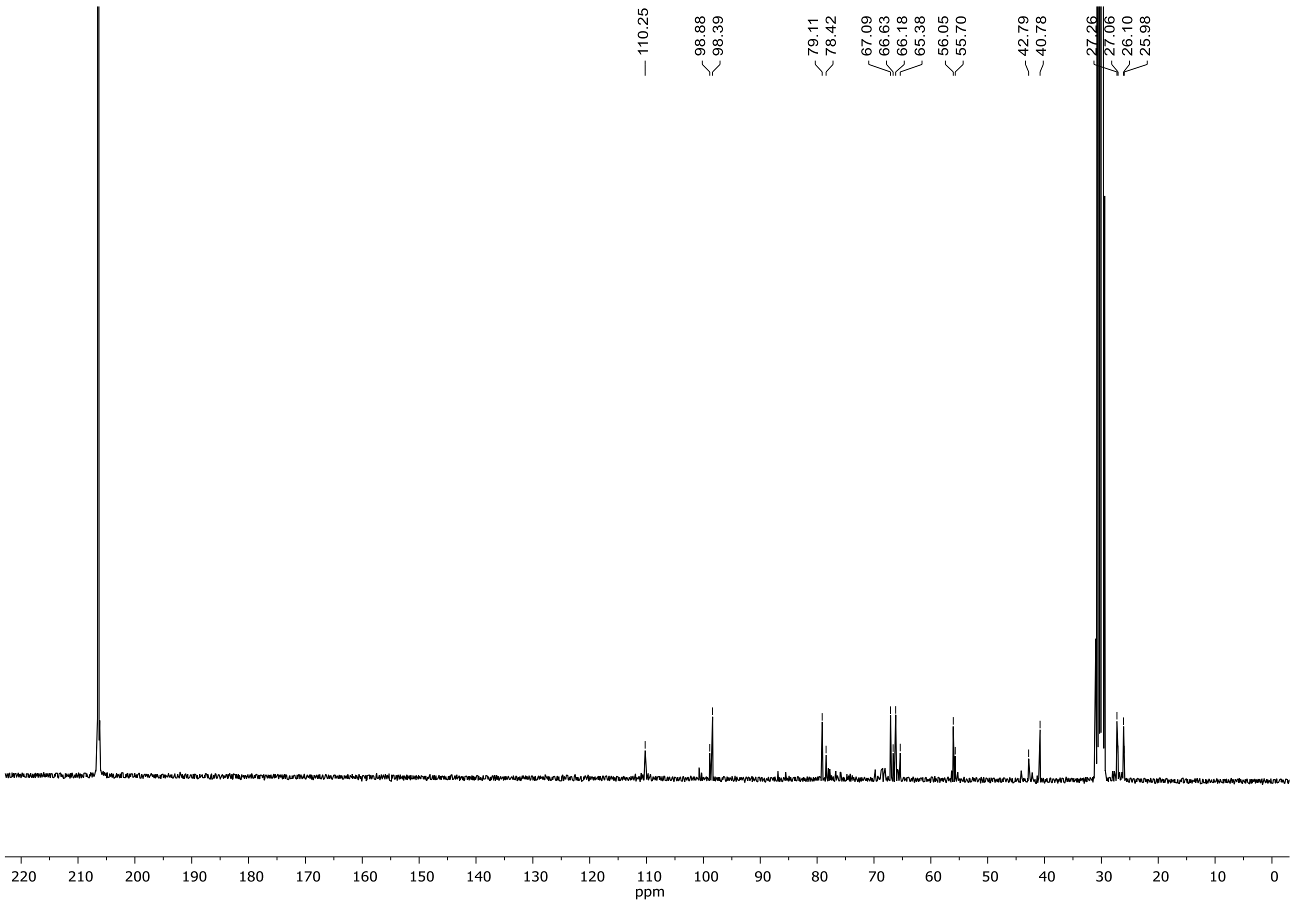

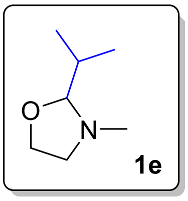

^{1}H NMR (300 MHz, acetone-d_6)

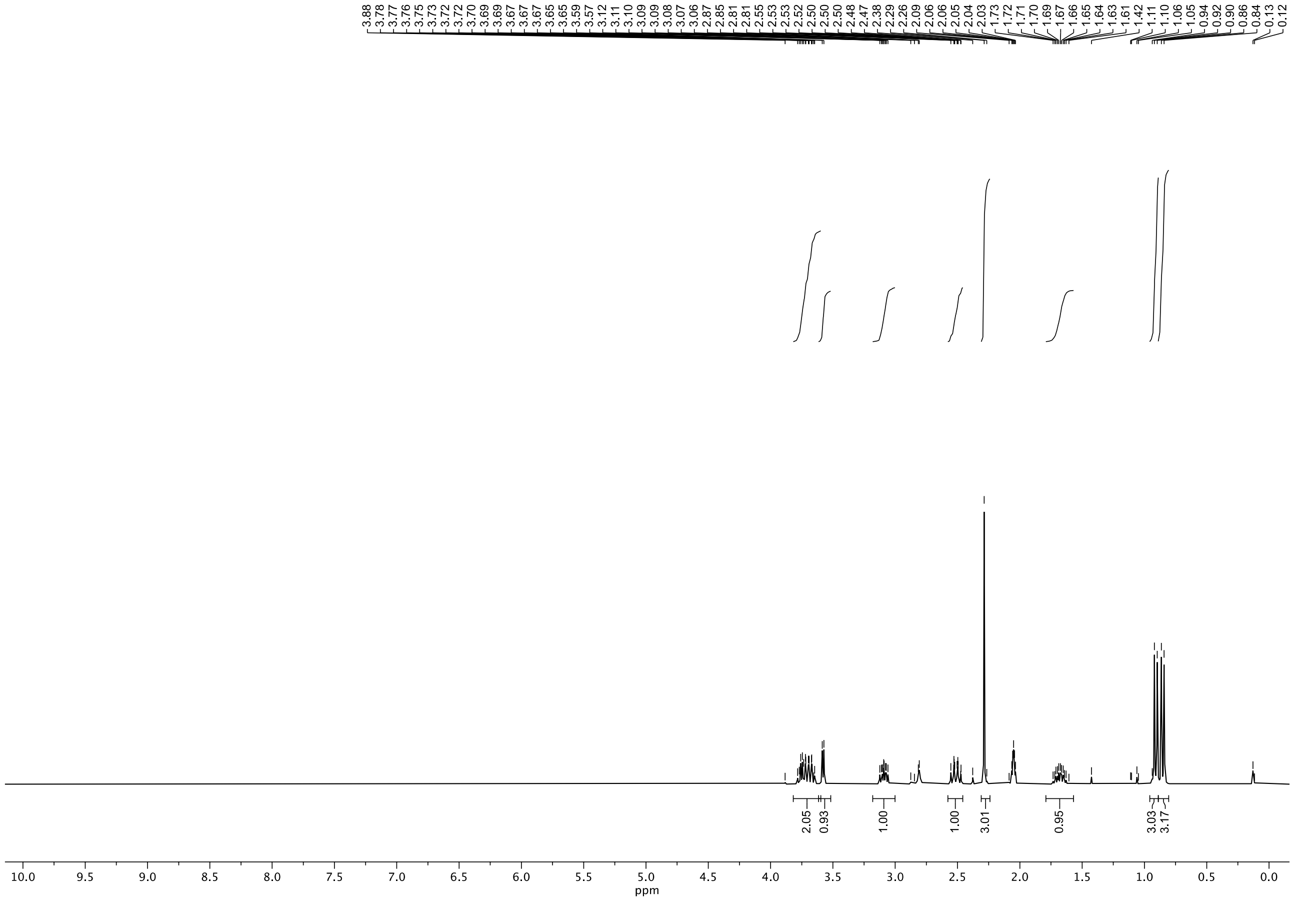

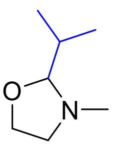

1e

^{13}C NMR (75 MHz, acetone-*d$_6$*)

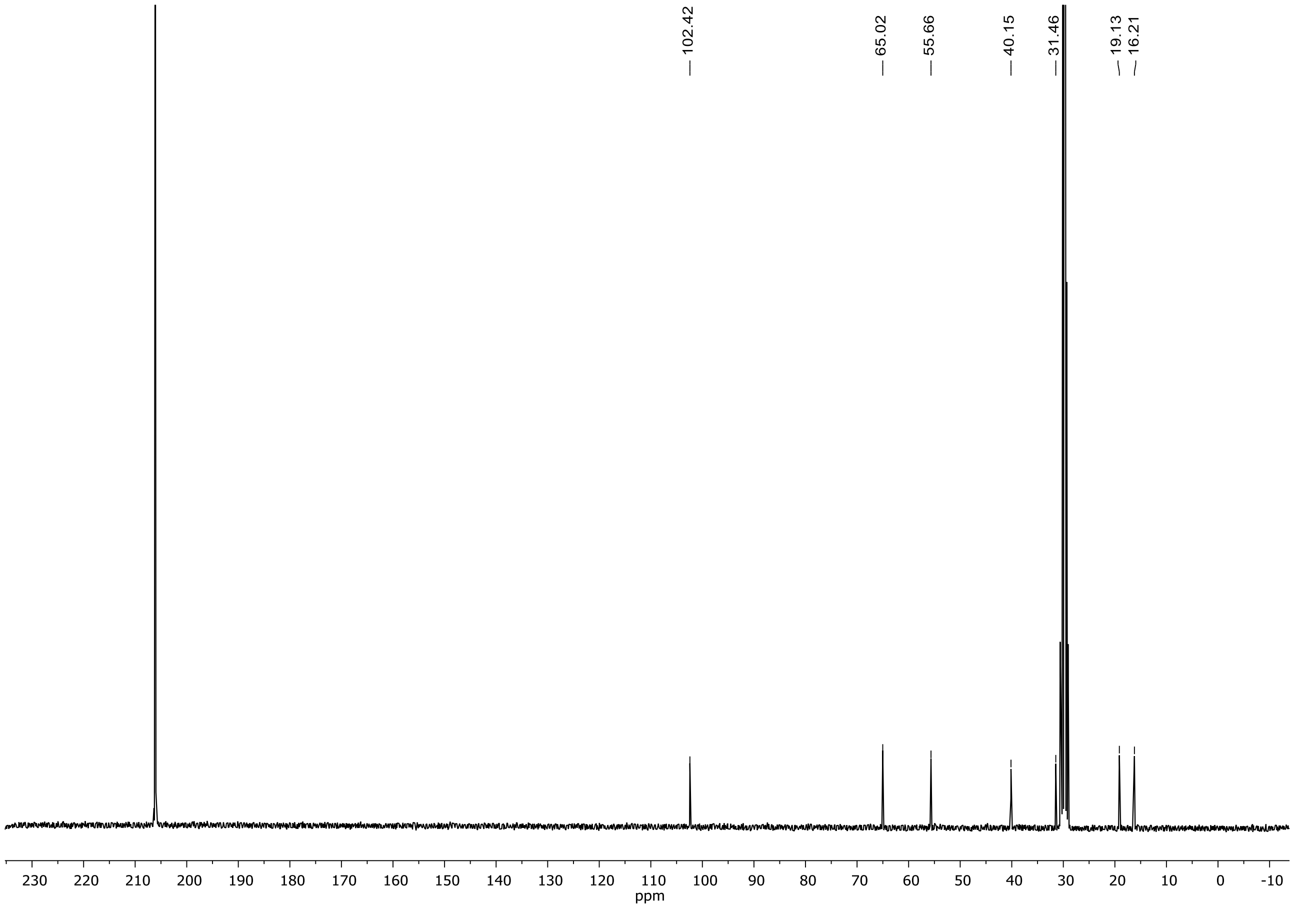

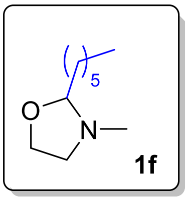

^{1}H NMR (300 MHz, acetone-d_6)

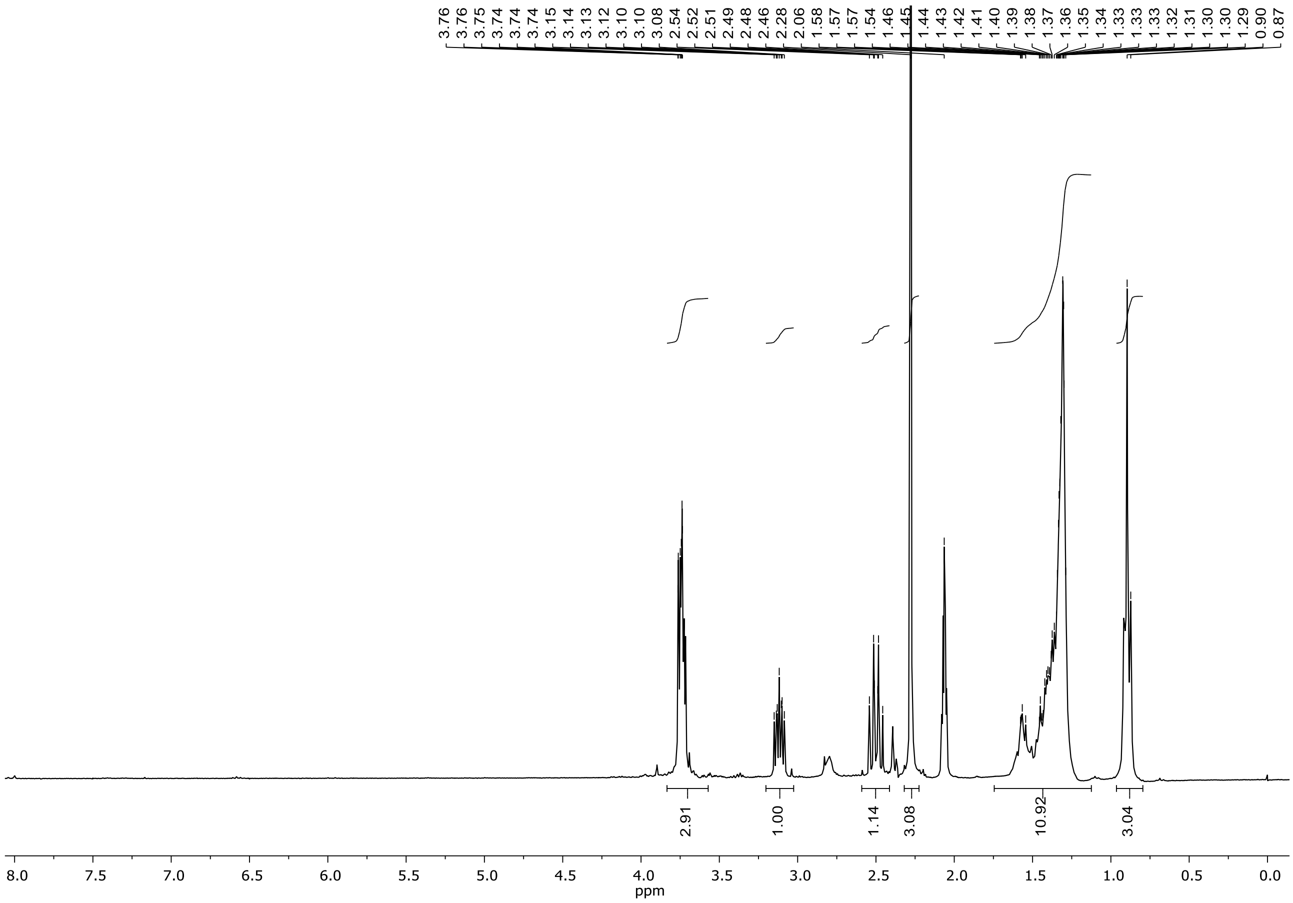

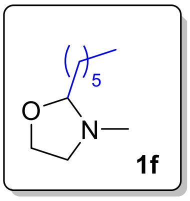

1f

^{13}C NMR (75 MHz, acetone-d_6)

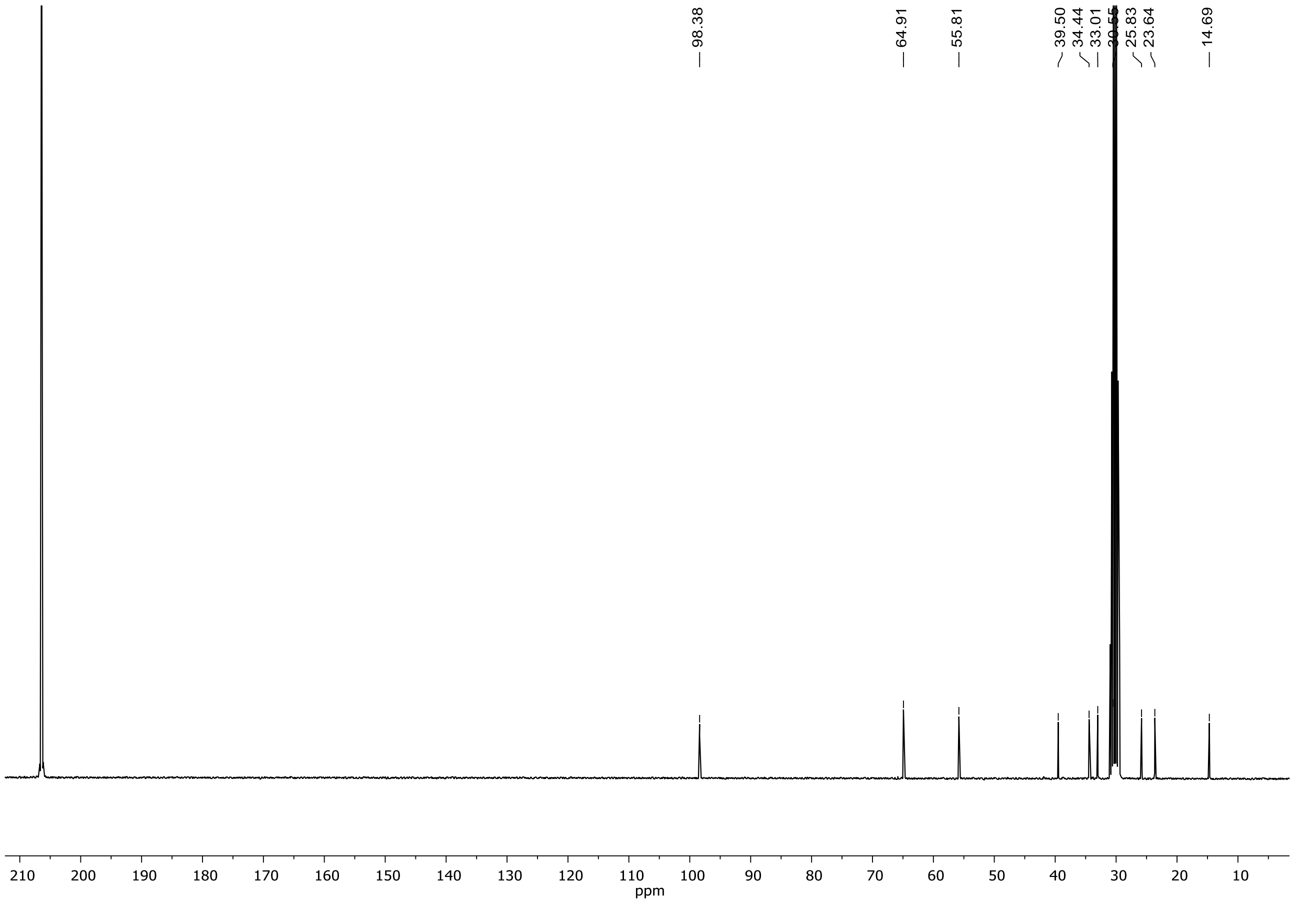

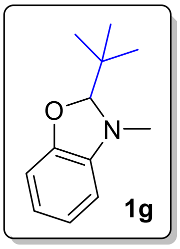

^{1}H NMR (300 MHz, acetone-d_6)

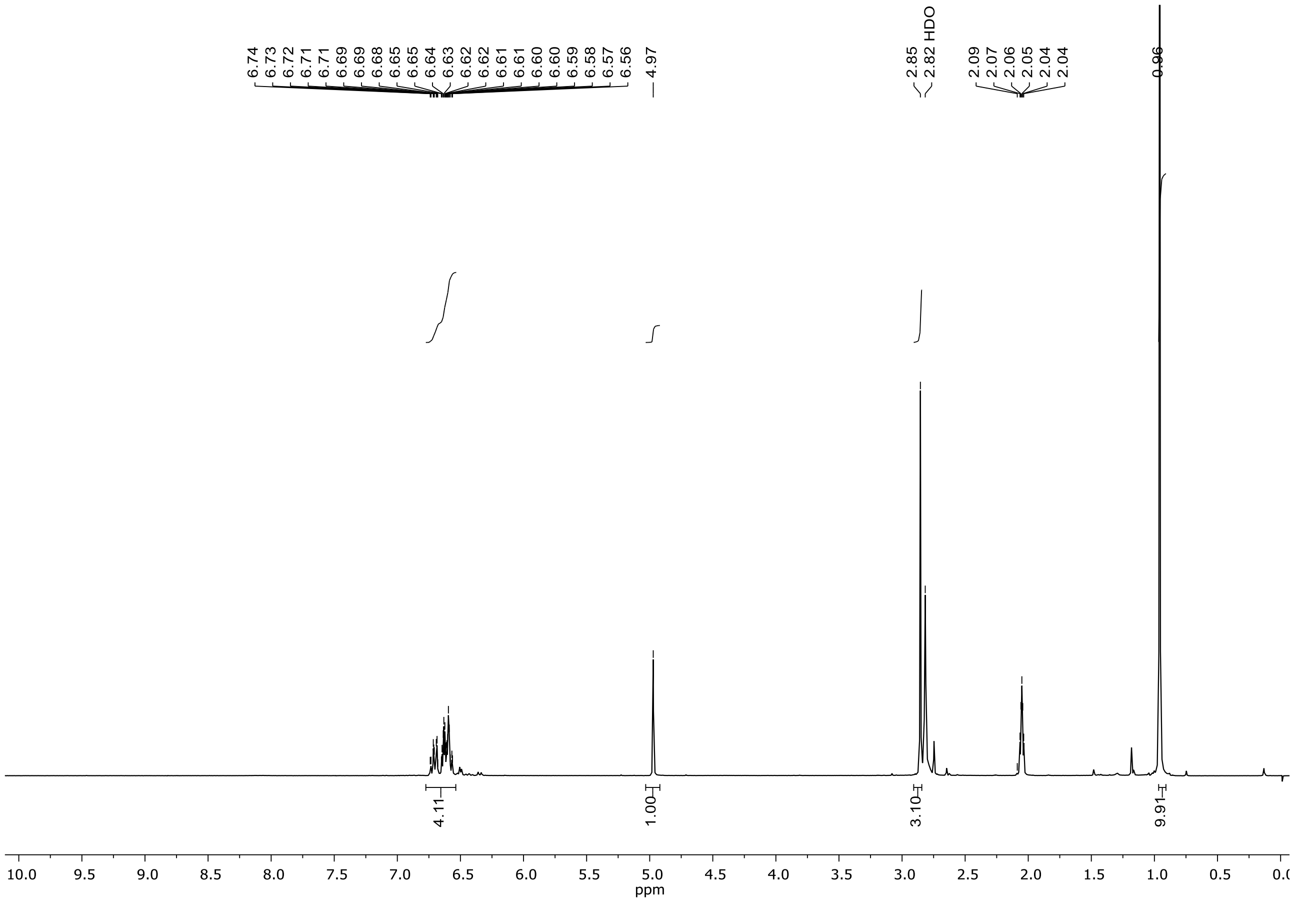

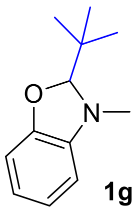

1g

^{13}C NMR (75 MHz, acetone-d_6)

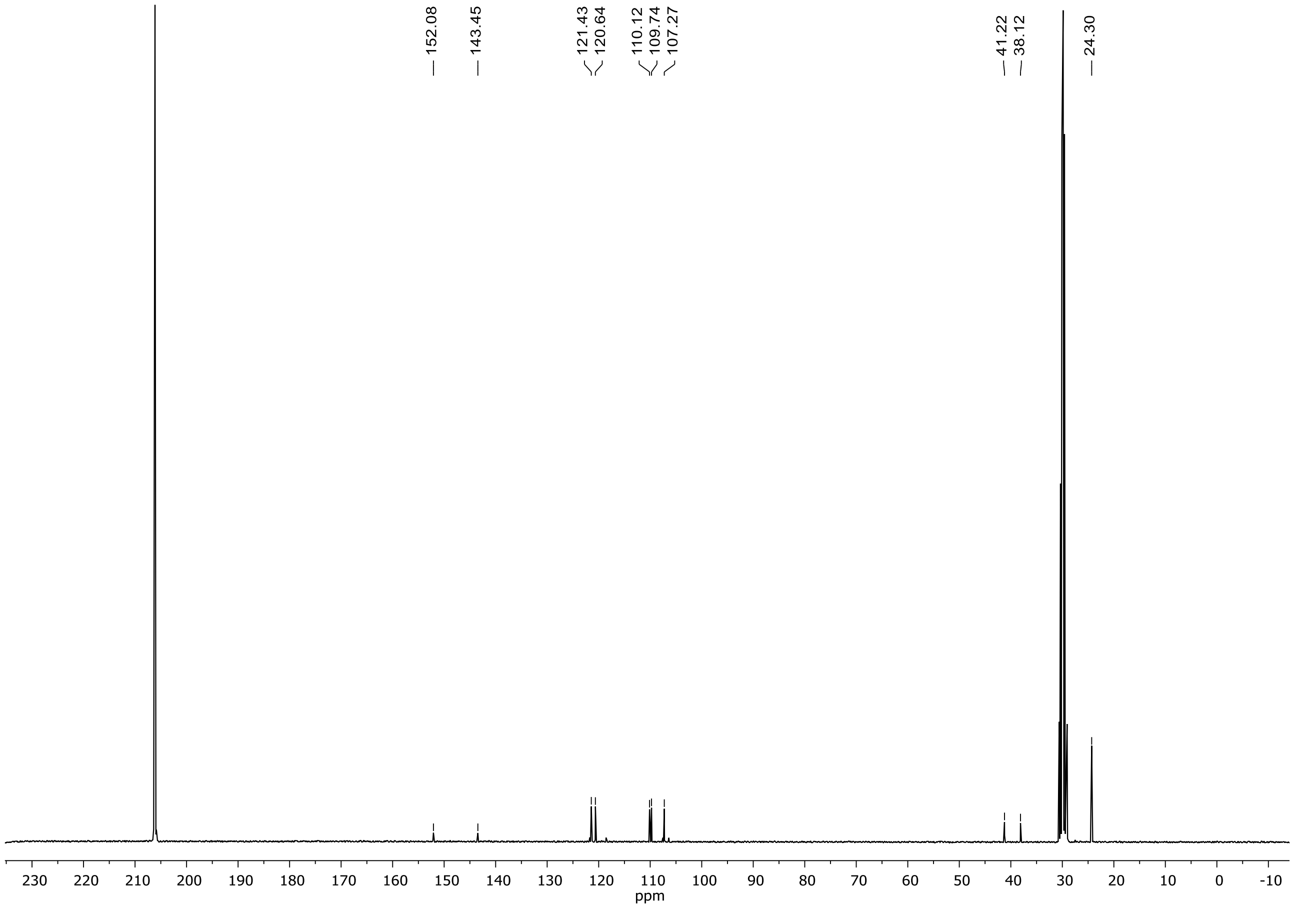

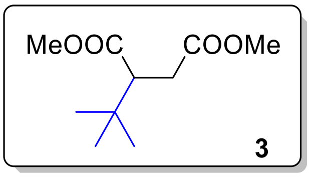

^{1}H NMR (300 MHz, chloroform-d)

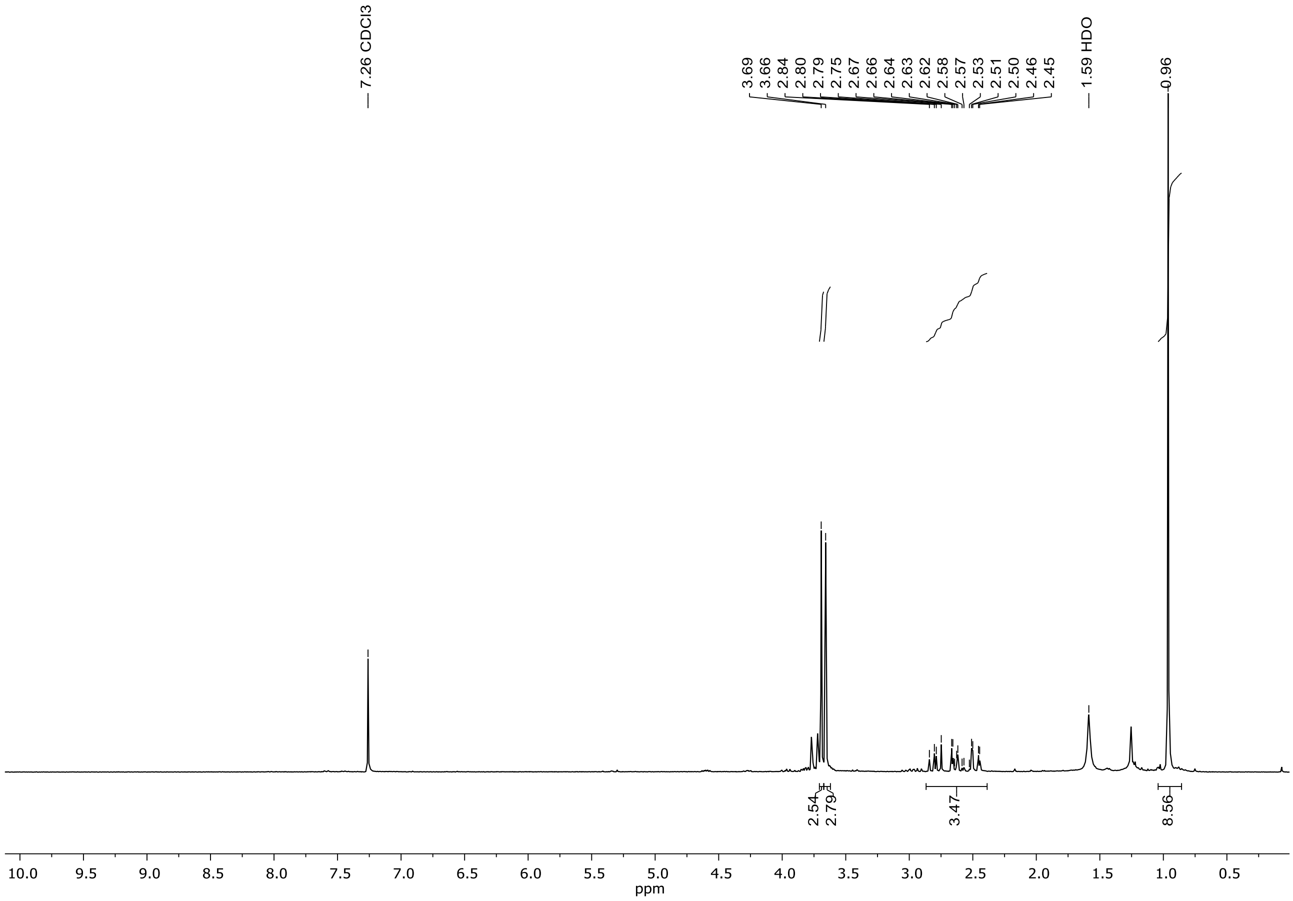

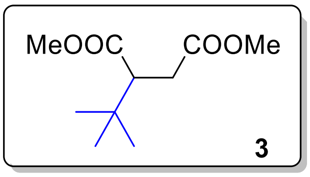

^{13}C NMR (75 MHz, chloroform-*d*)

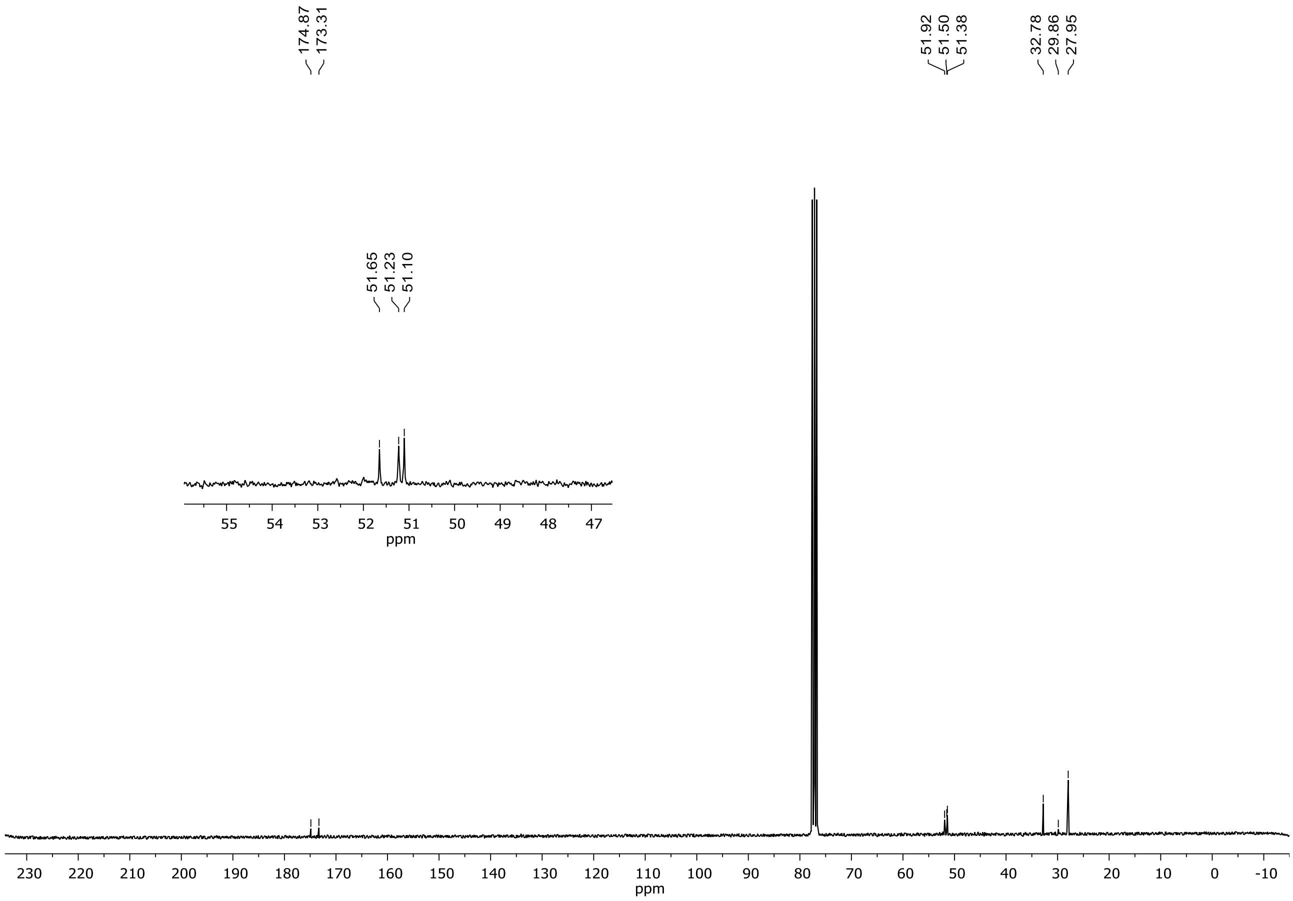

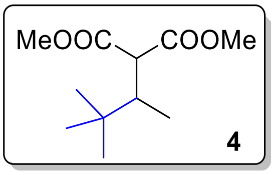

^{1}H NMR (300 MHz, chloroform-*d*)

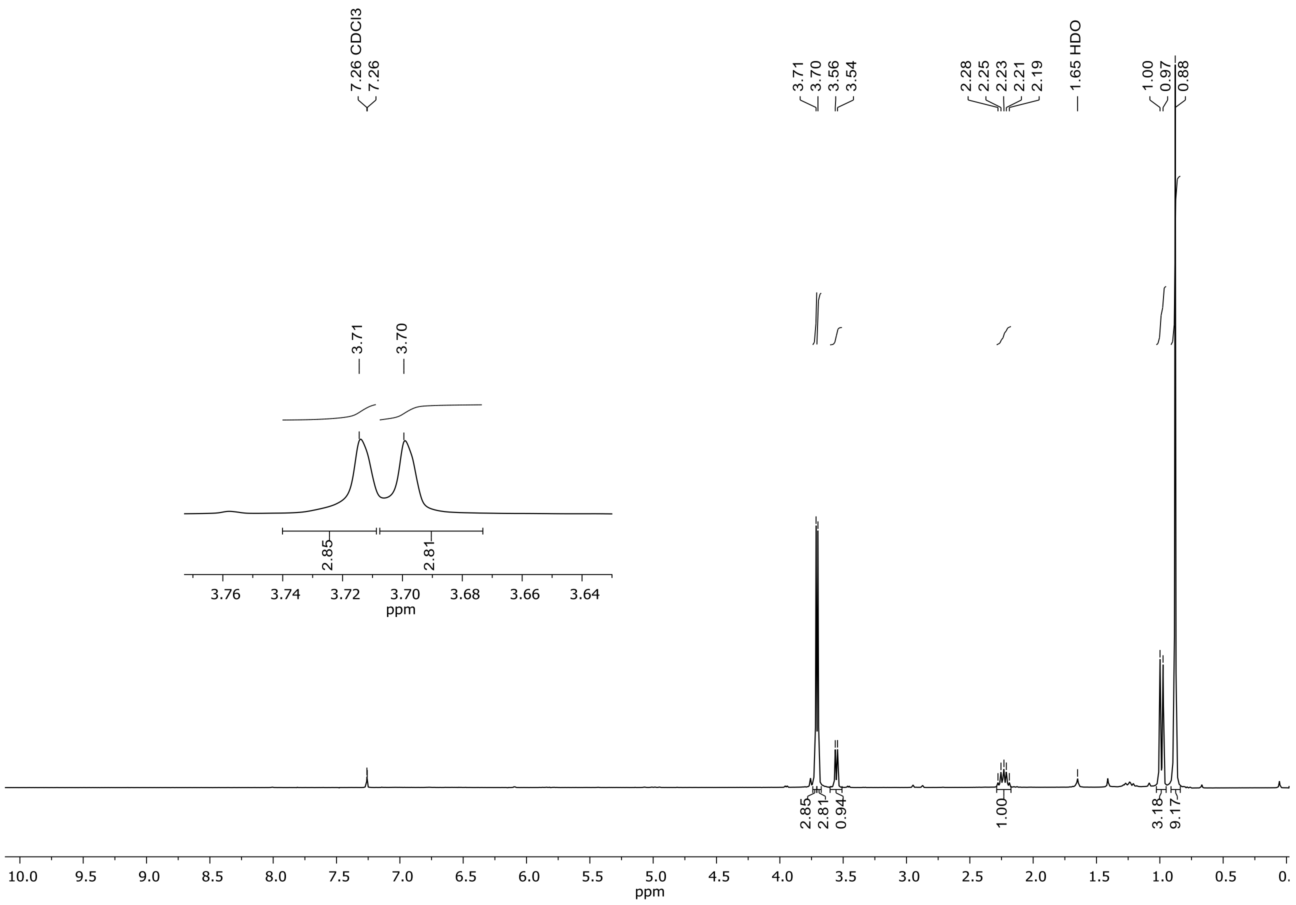

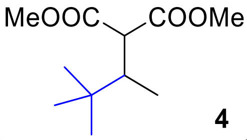

4

^{13}C NMR (75 MHz, chloroform-*d*)

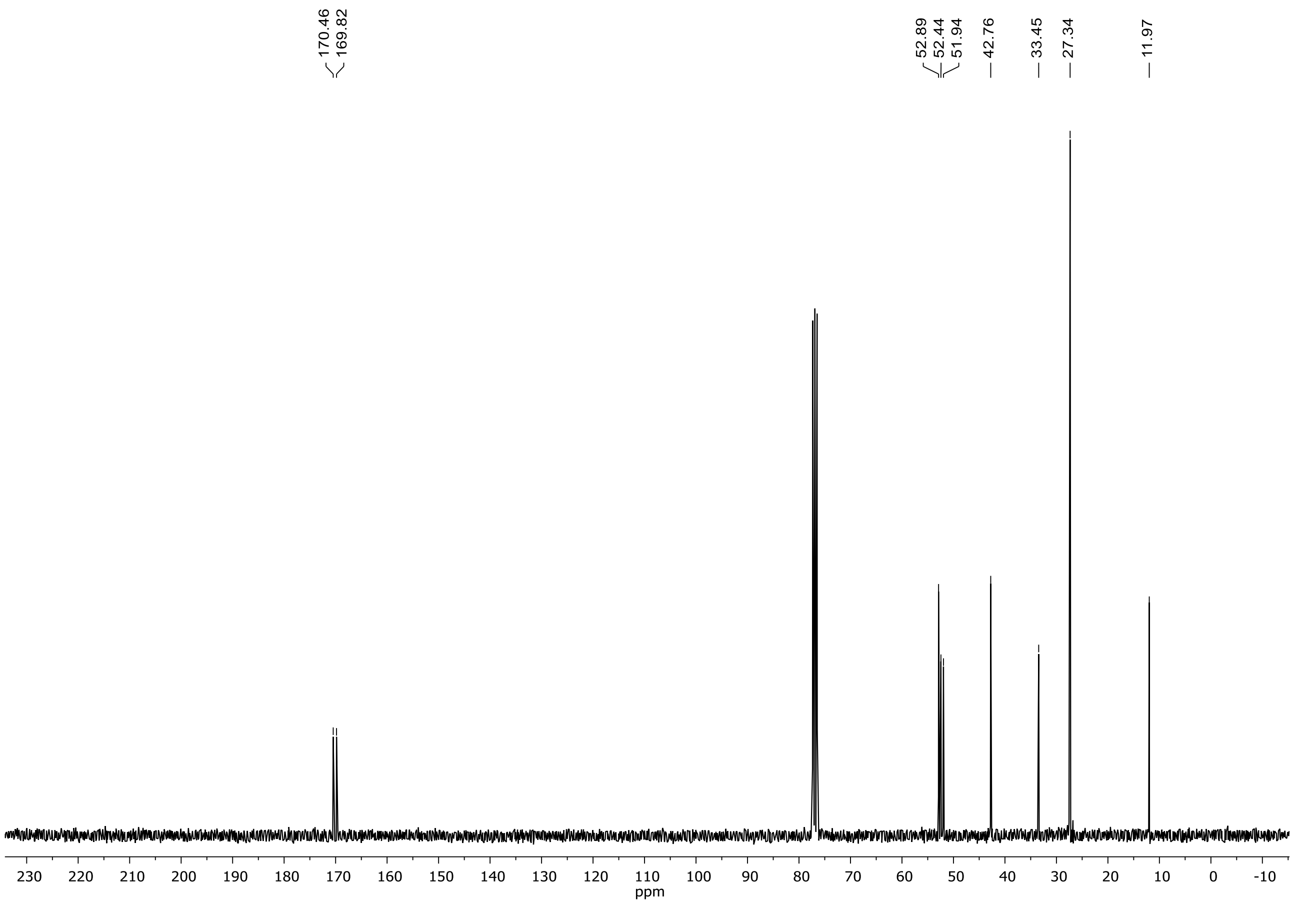

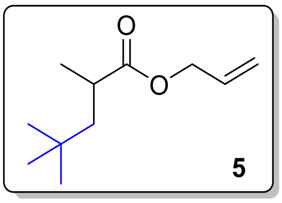

5

^{1}H NMR (300 MHz, chloroform-*d*)

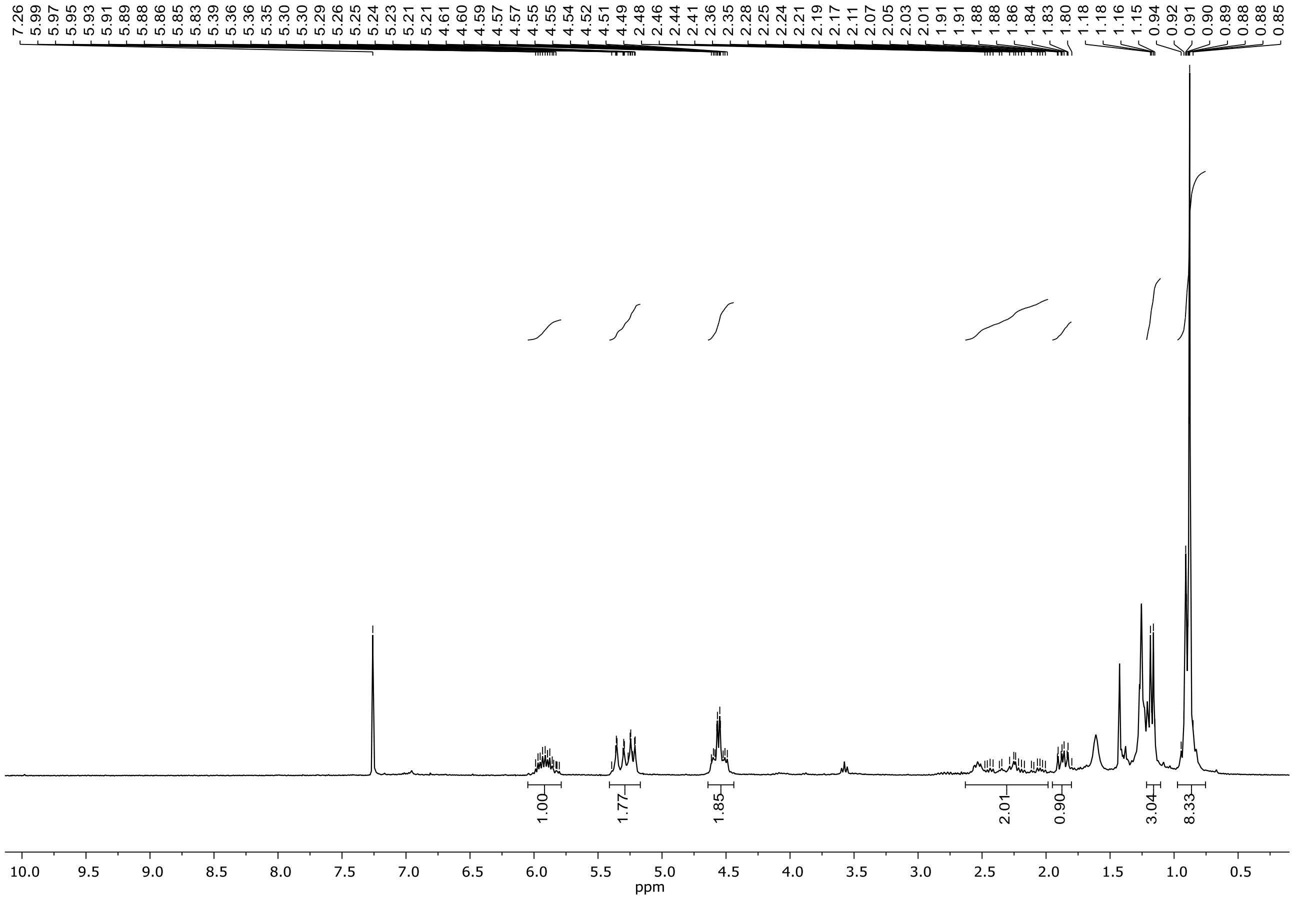

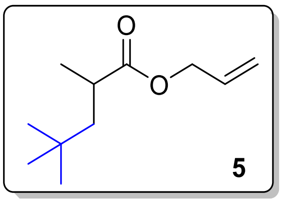

^{13}C NMR (75 MHz, chloroform-*d*)

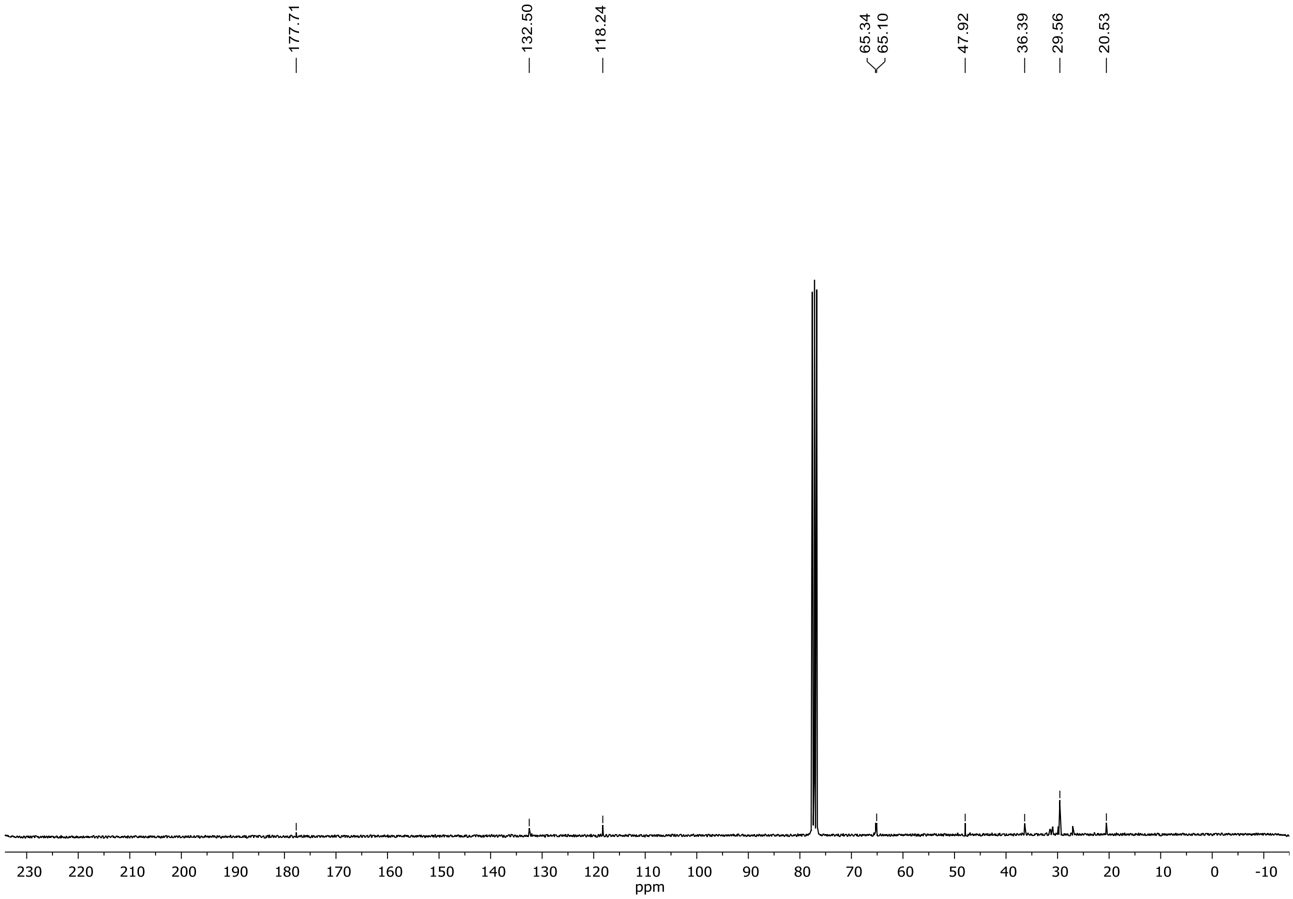

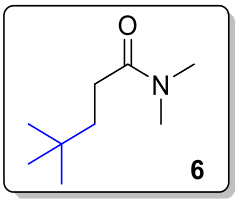

^{1}H NMR (300 MHz, chloroform-*d*)

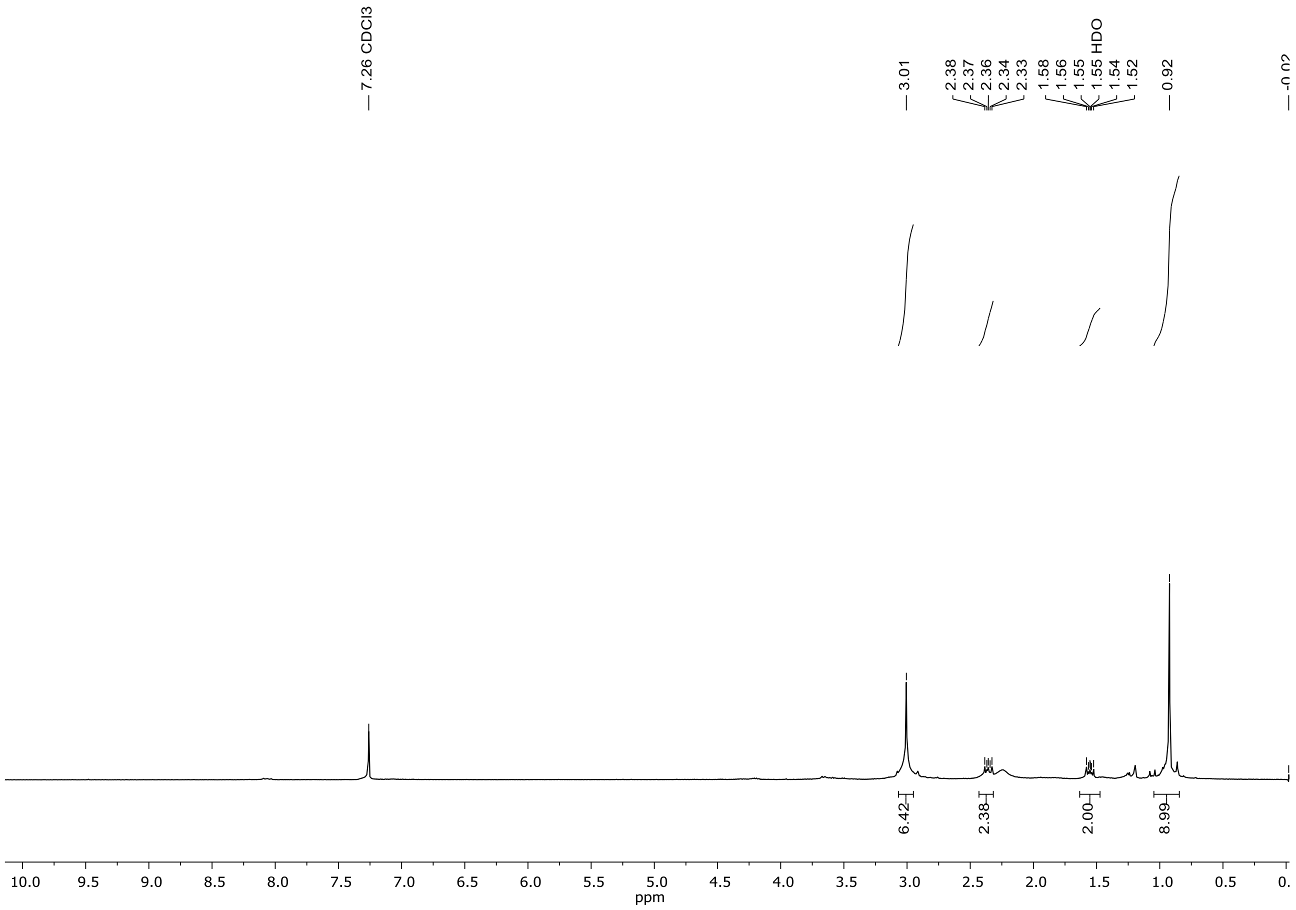

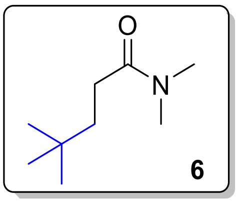

^{13}C NMR (75 MHz, chloroform-*d*)

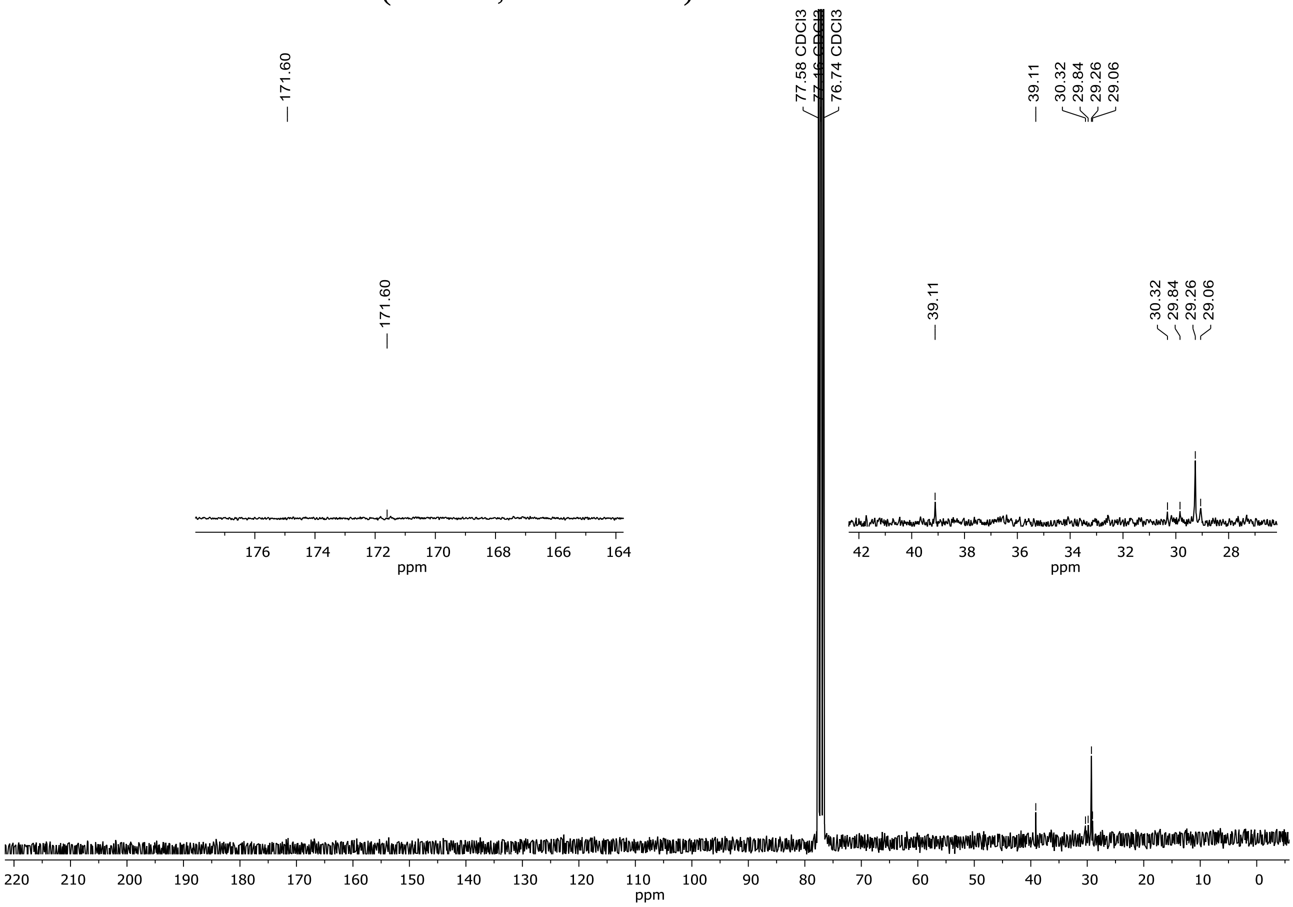

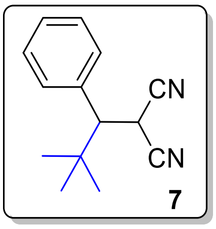

^{1}H NMR (300 MHz, acetone-d_6)

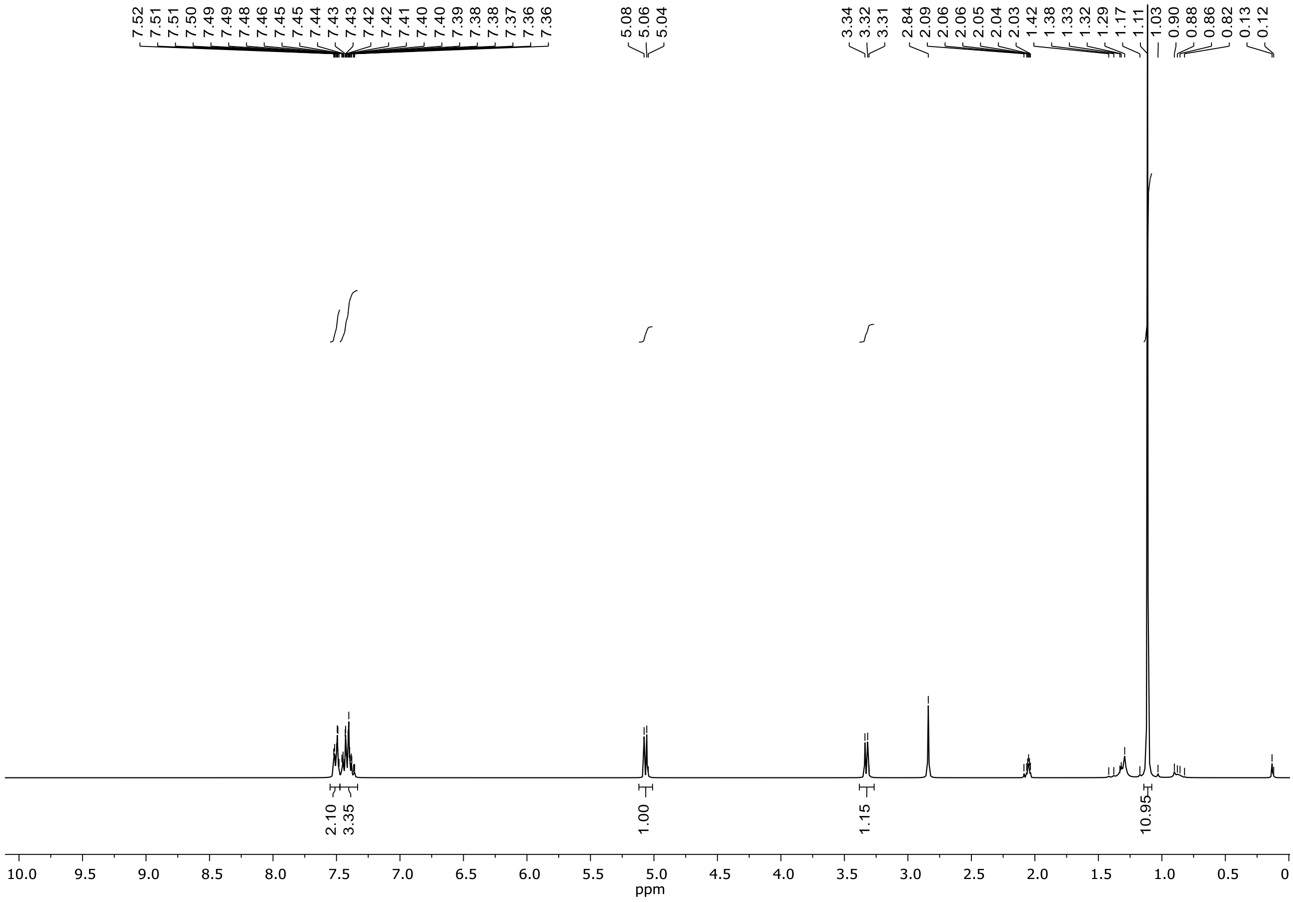

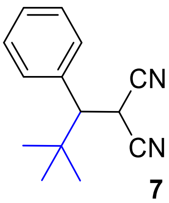

7

^{13}C NMR (75 MHz, acetone-d_6)

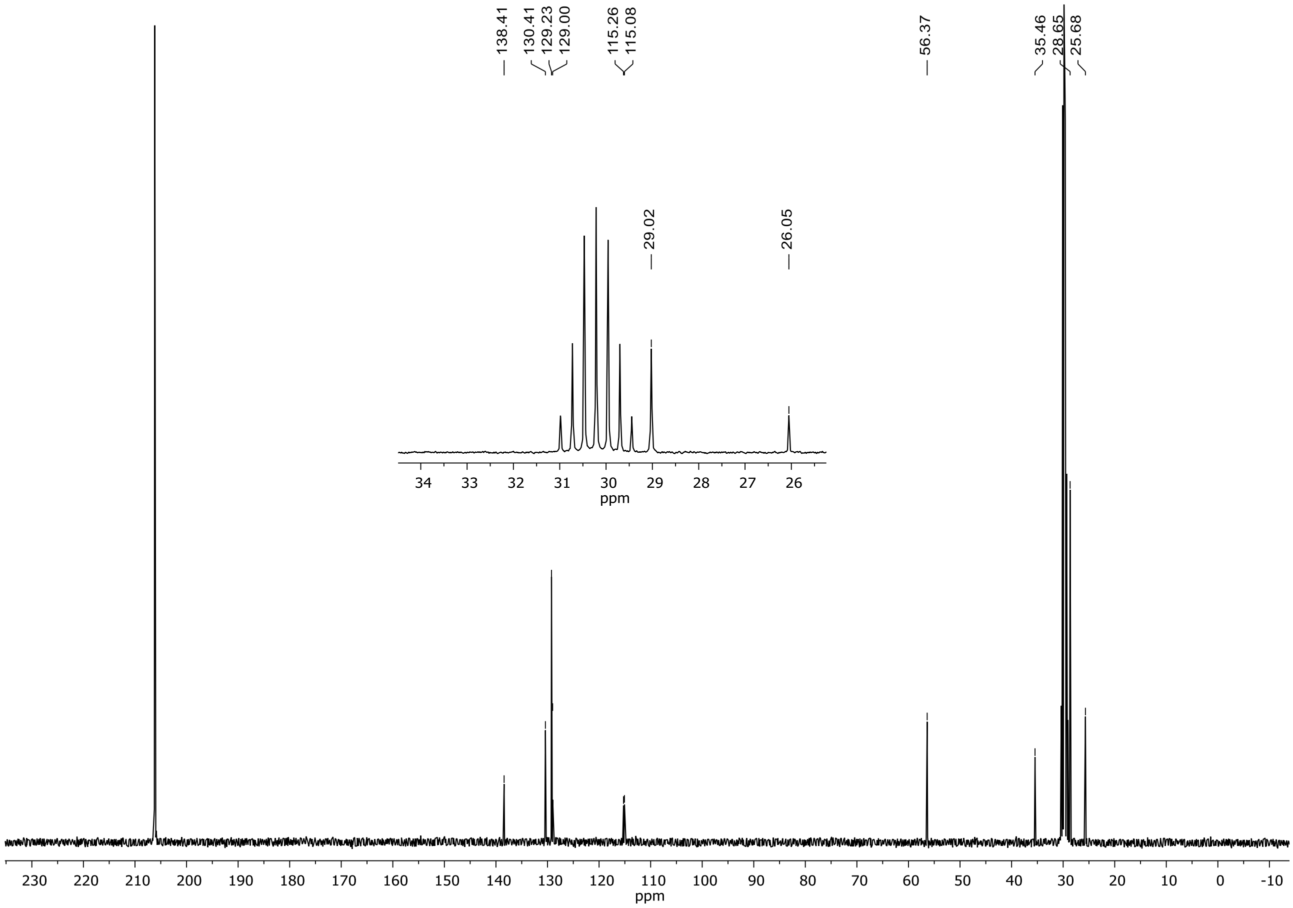

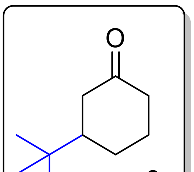
8

^{1}H NMR (300 MHz, chloroform-*d*)

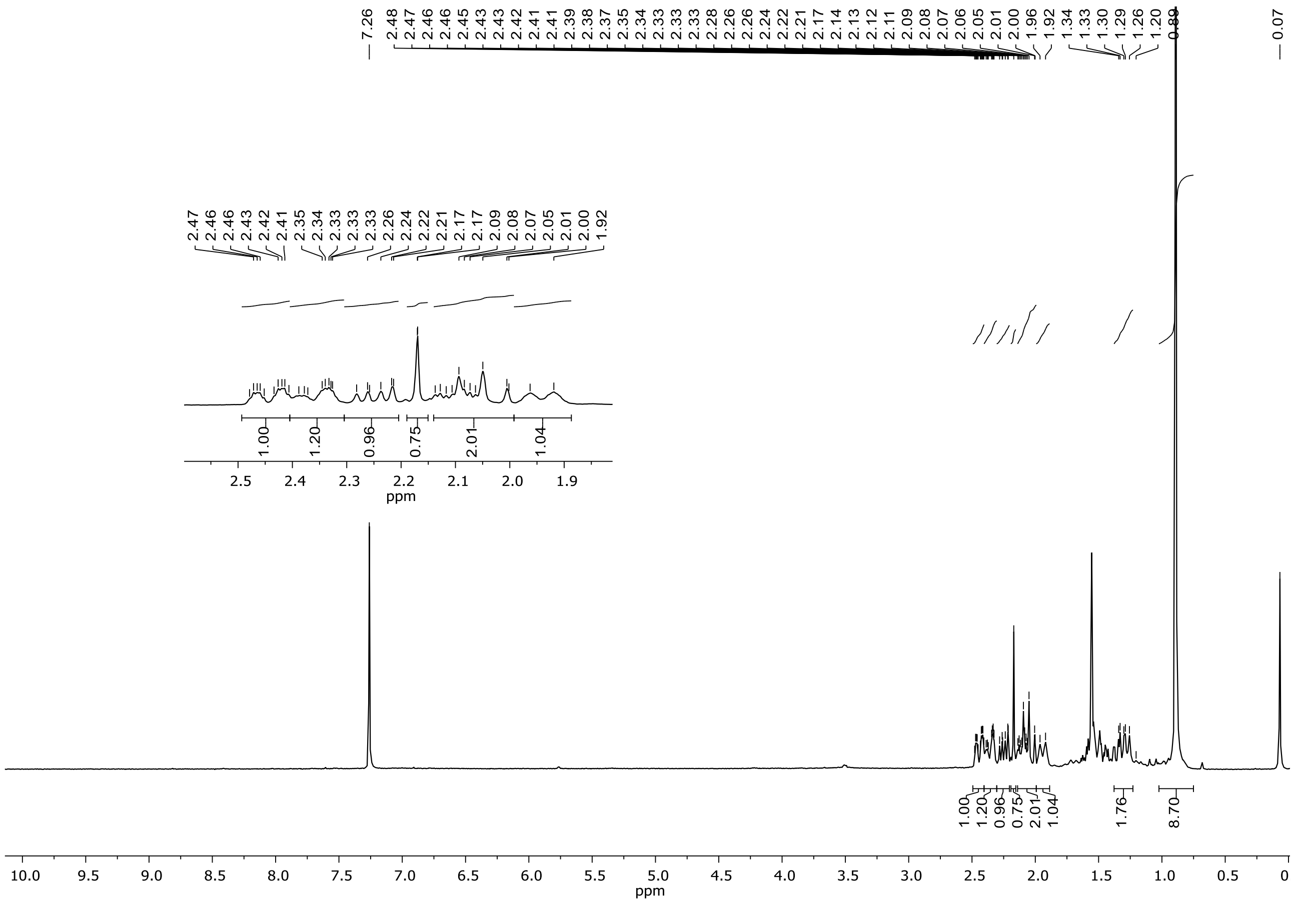

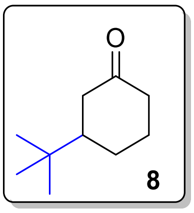

^{13}C NMR (75 MHz, chloroform-*d*)

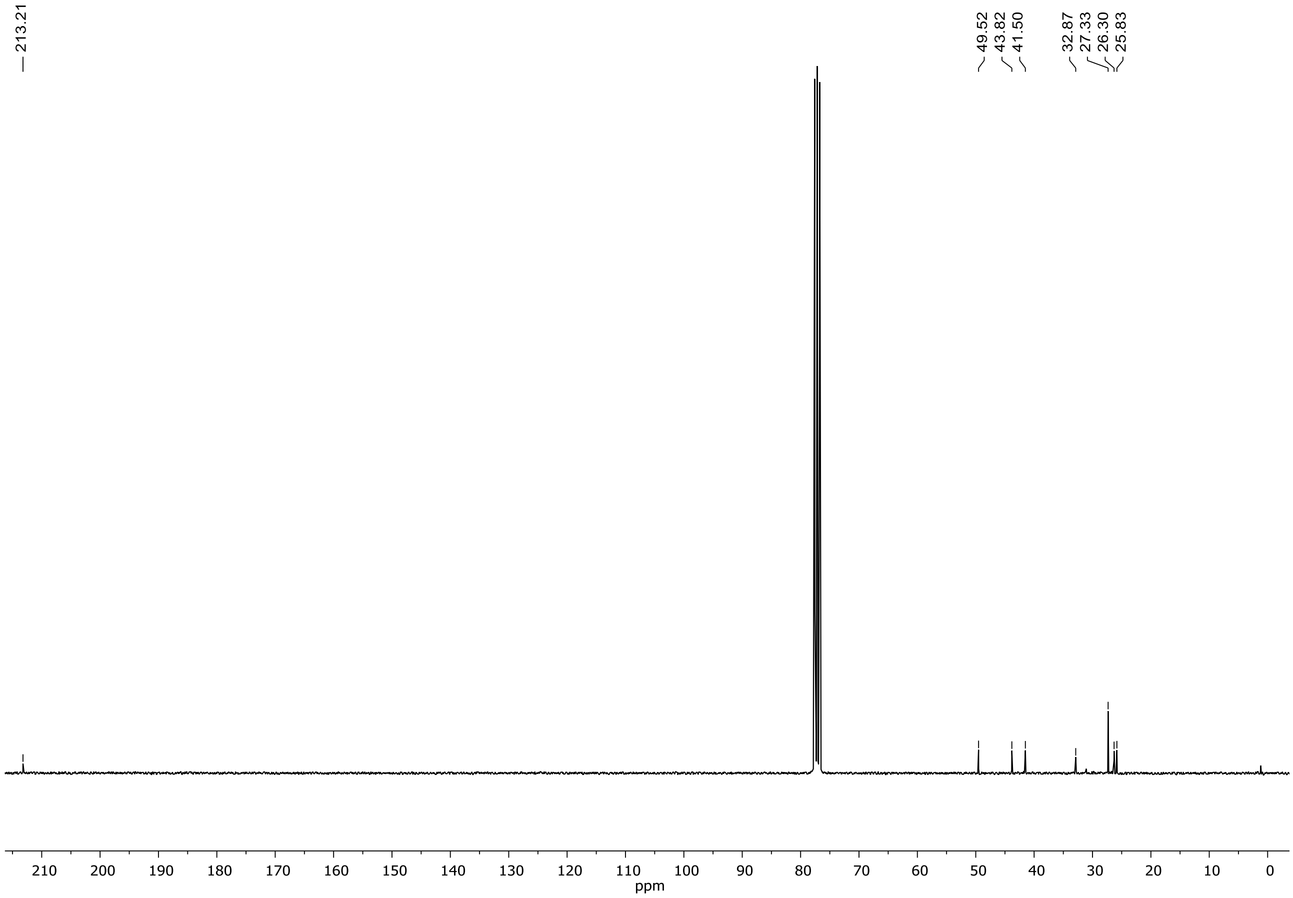

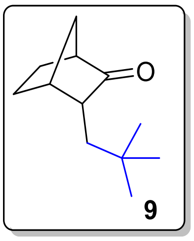

^{1}H NMR (300 MHz, acetone-d_6)

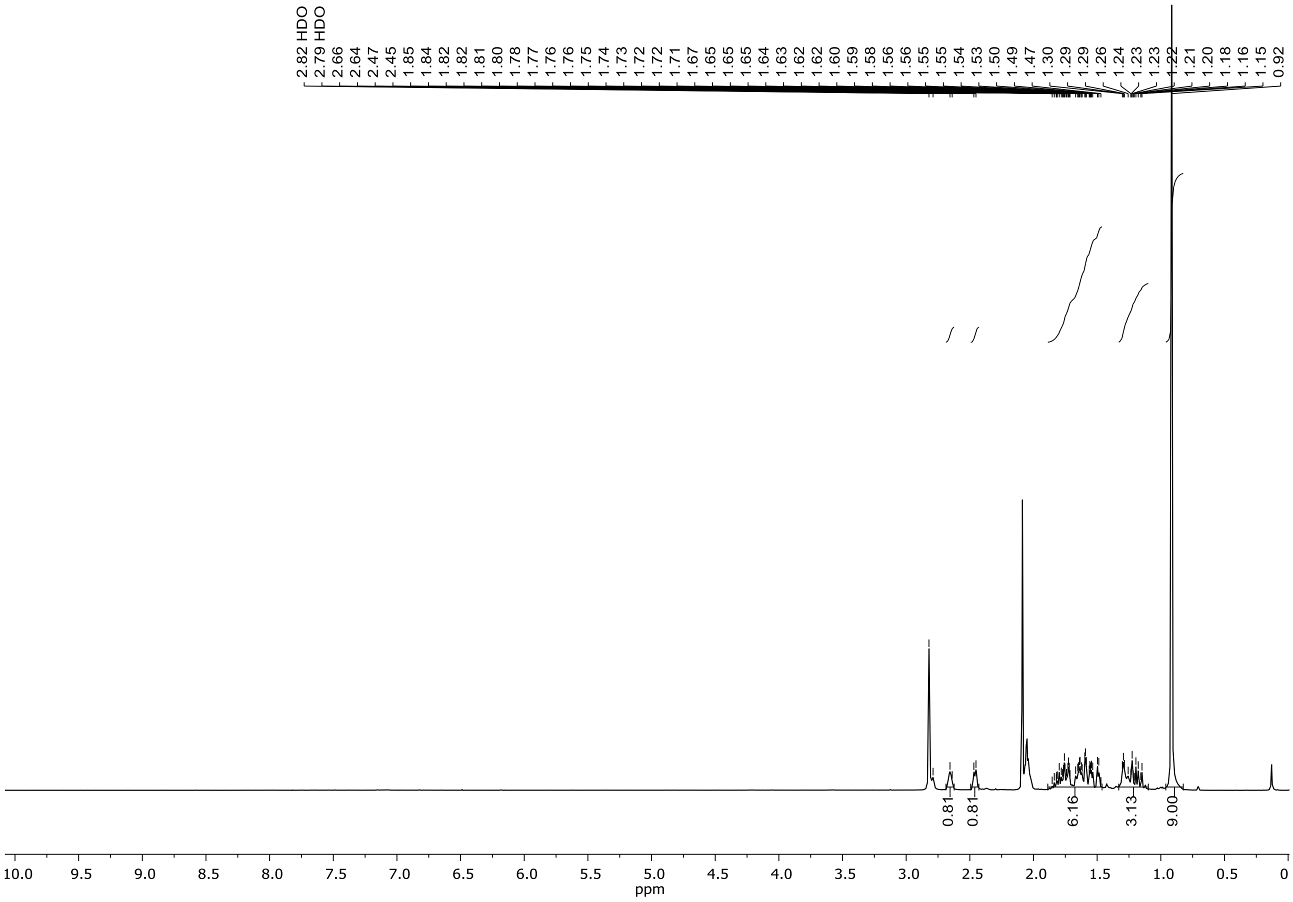

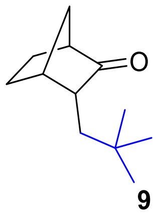

9

^{13}C NMR (75 MHz, acetone-d_6)

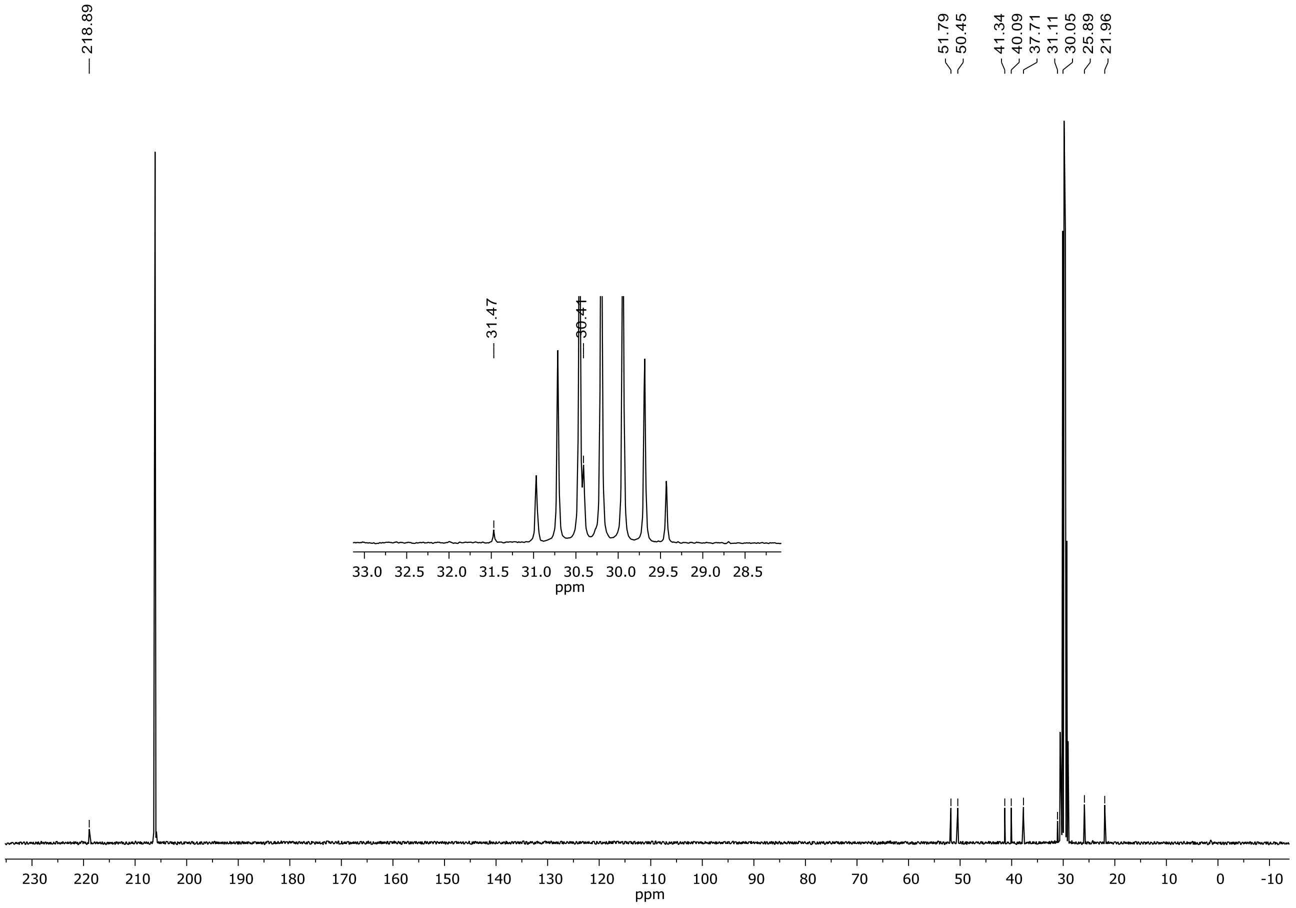

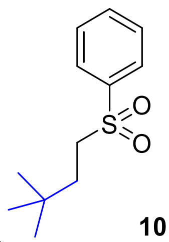

10

^{1}H NMR (300 MHz, chloroform-*d*)

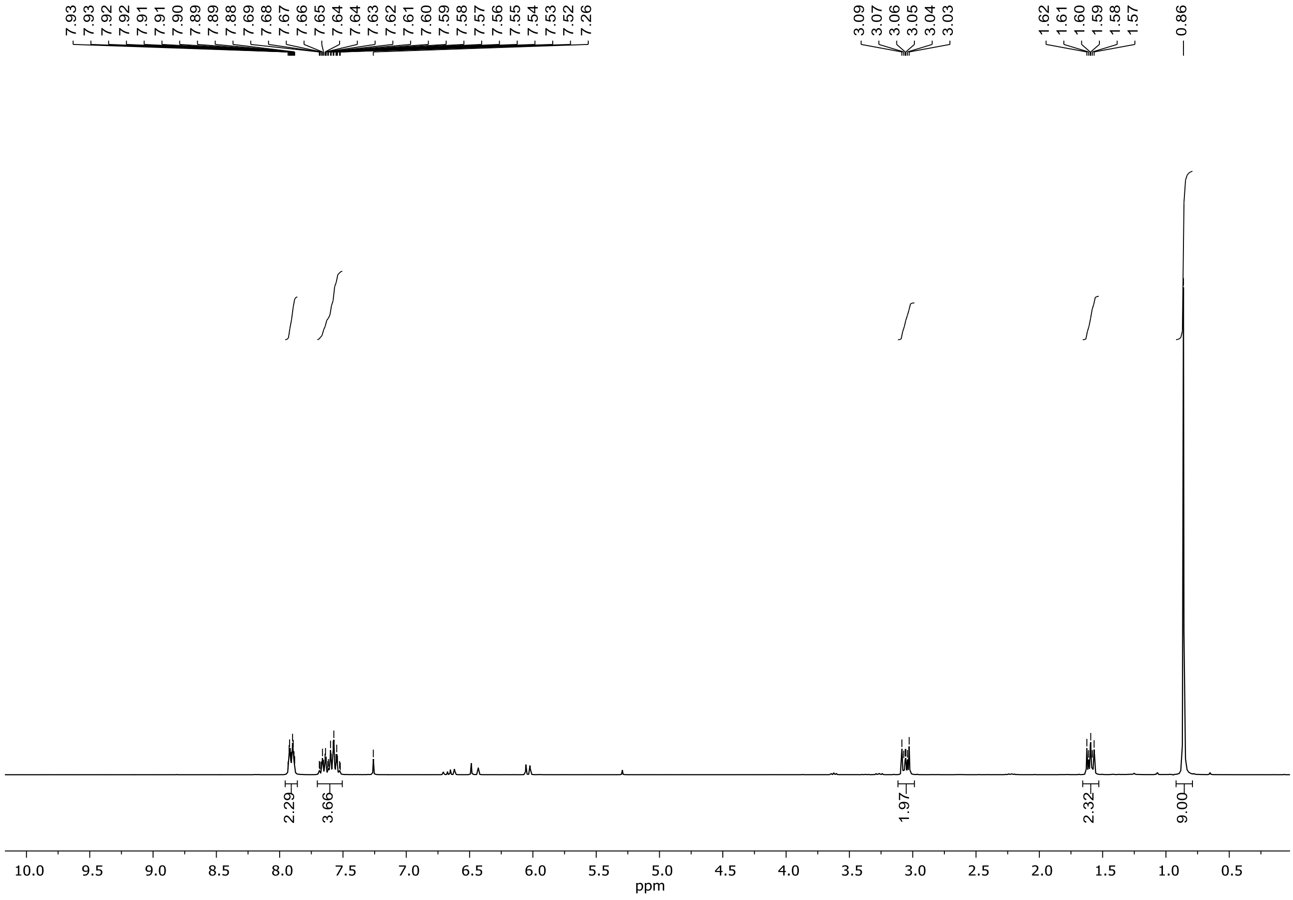

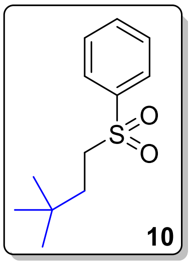

^{13}C NMR (75 MHz, chloroform-*d*)

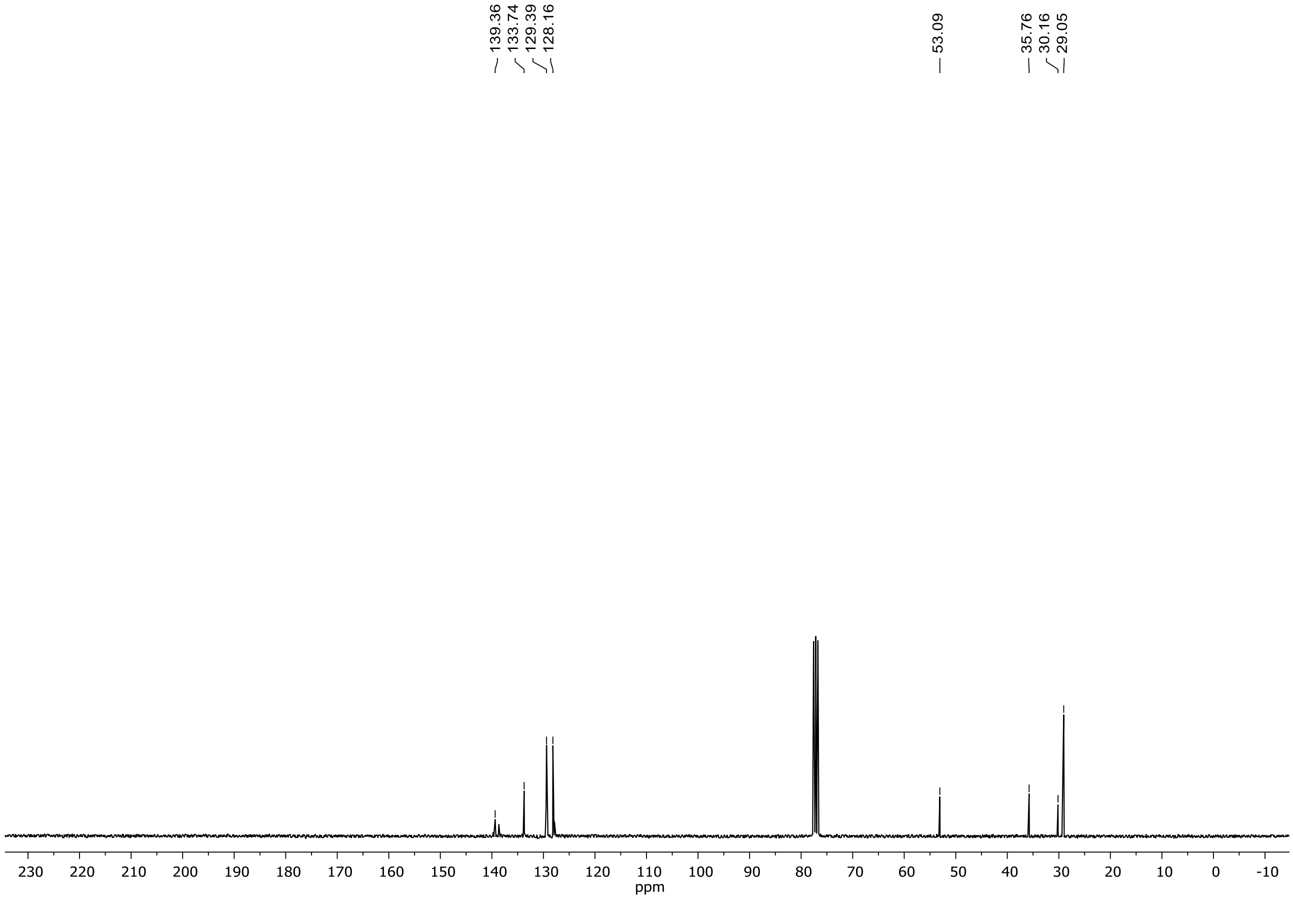

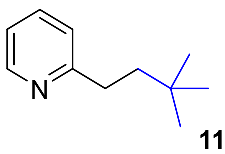

11

^{1}H NMR (300 MHz, chloroform-*d*)

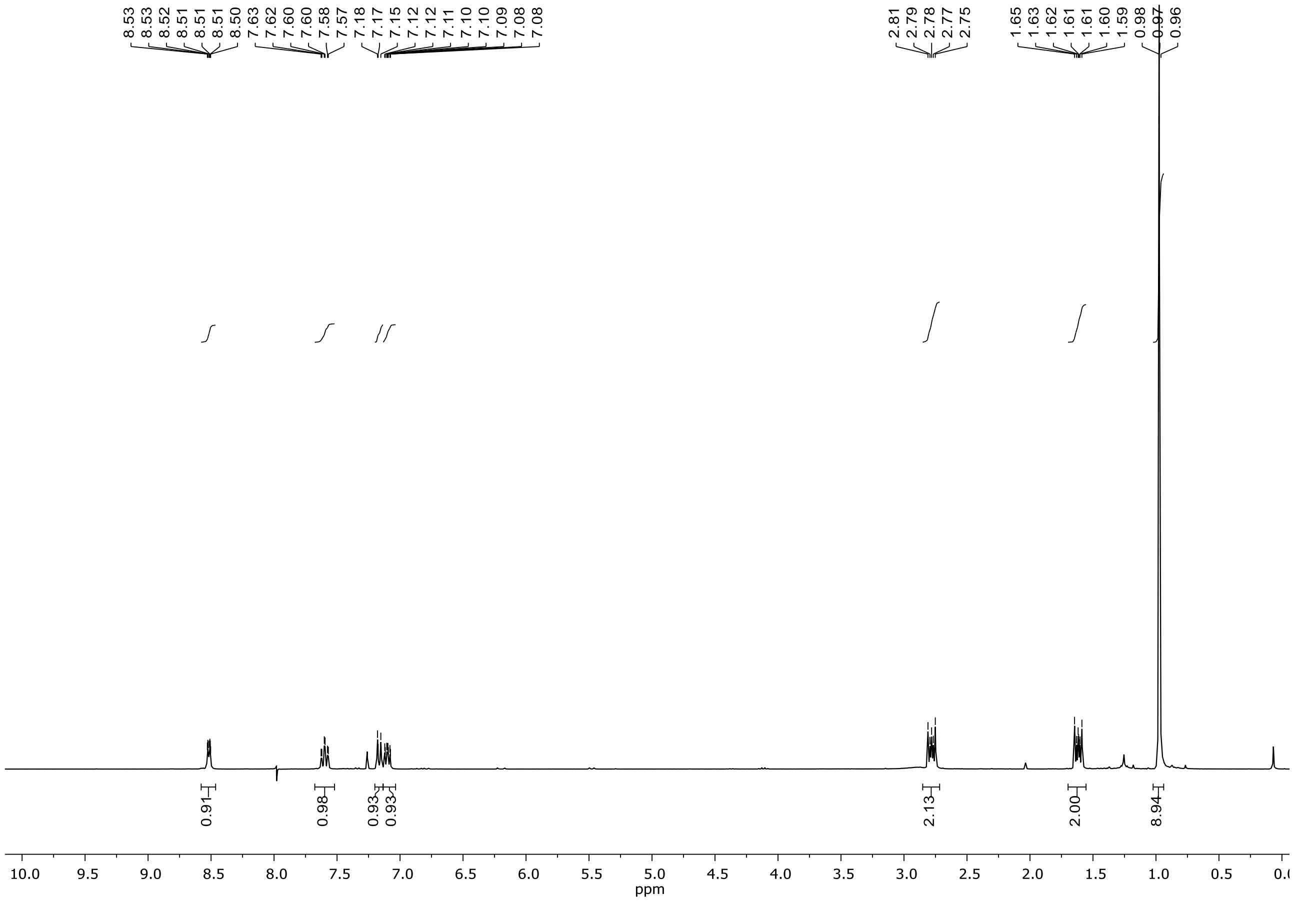

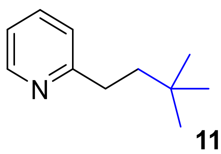

11

^{13}C NMR (75 MHz, chloroform-d_6)

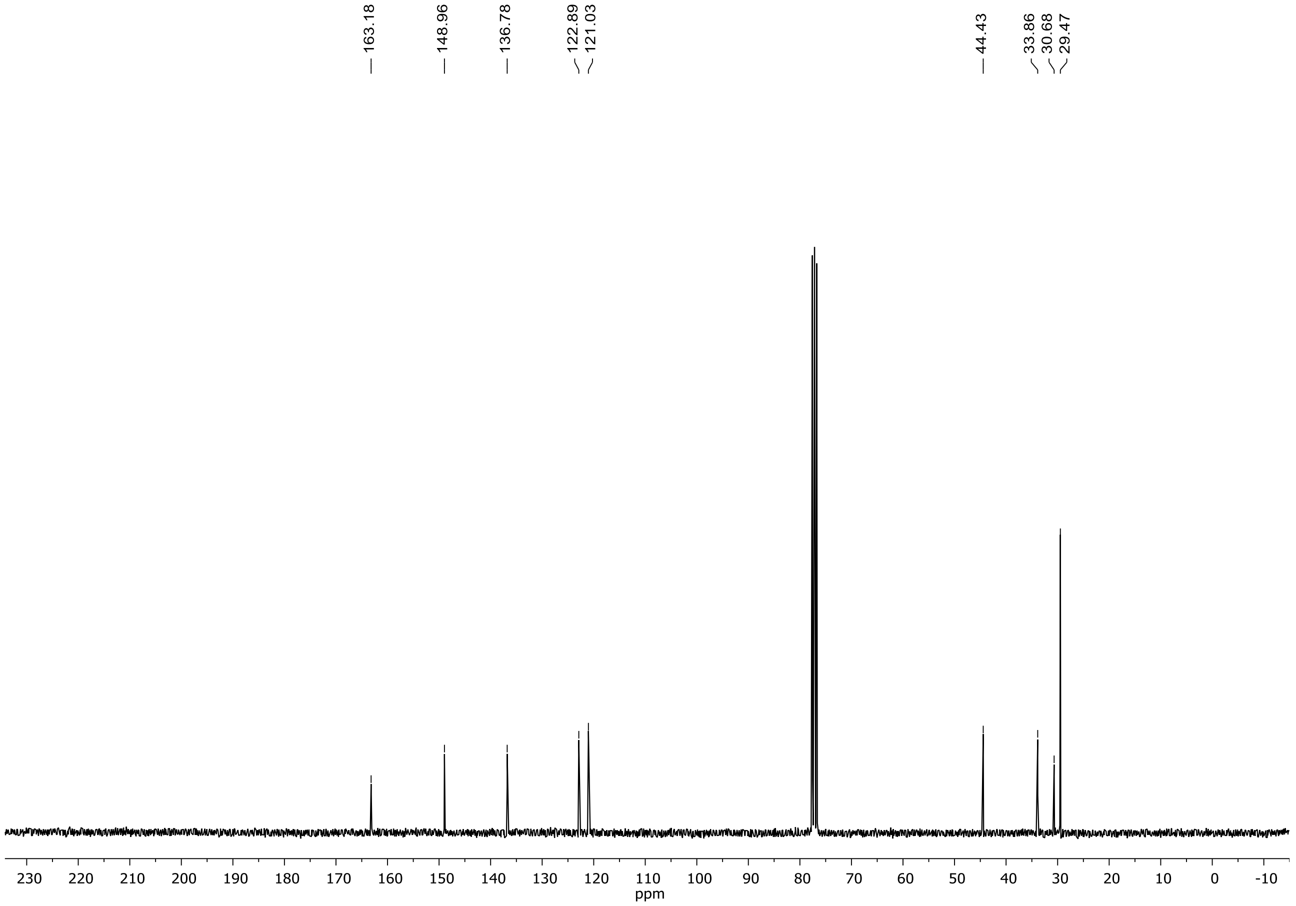

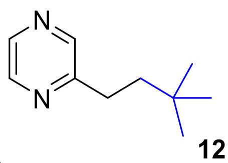

12

^{1}H NMR (300 MHz, chloroform-d)

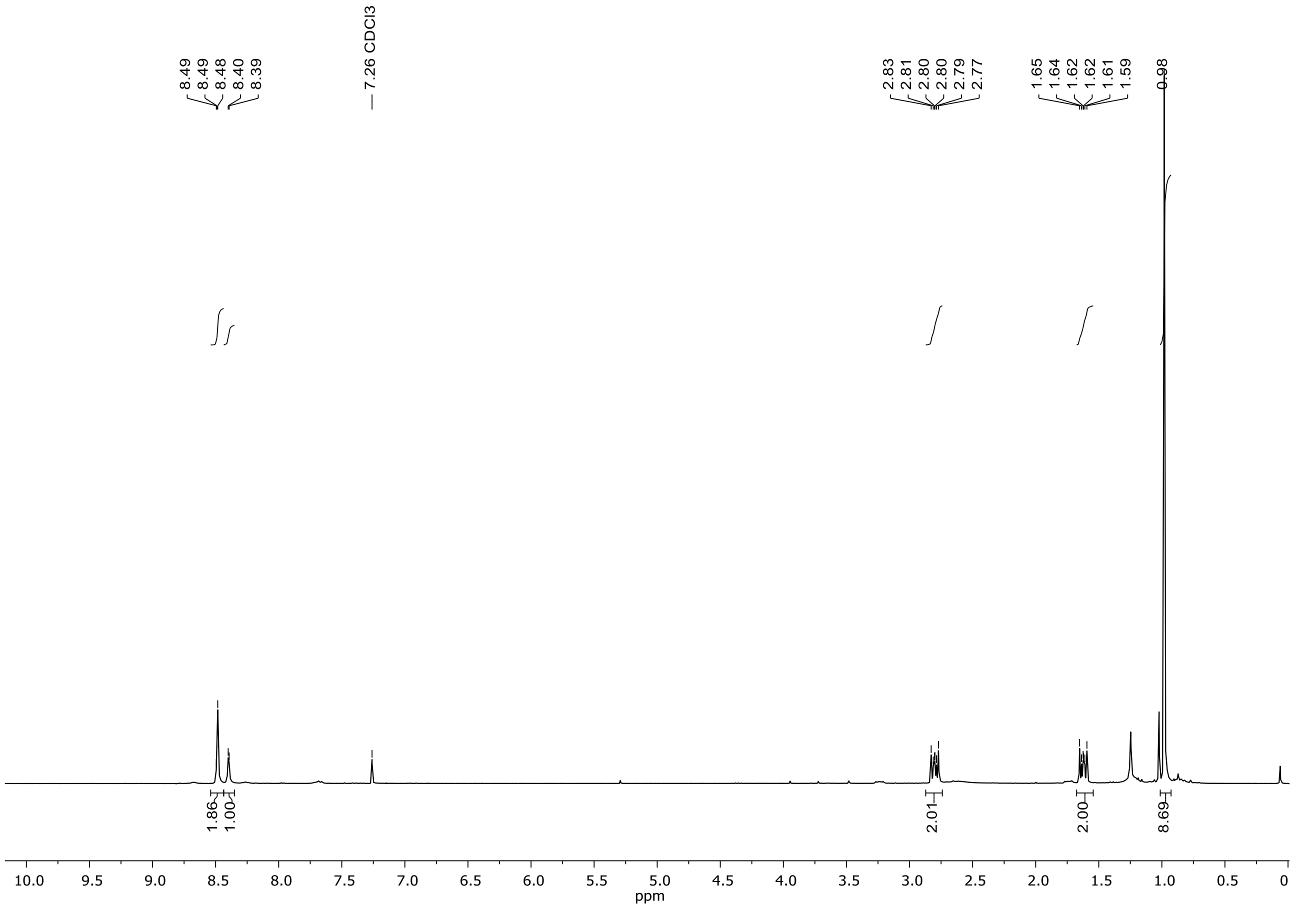

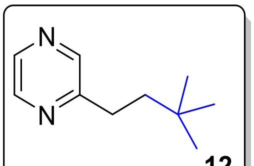

12

^{13}C NMR (75 MHz, chloroform-*d*)

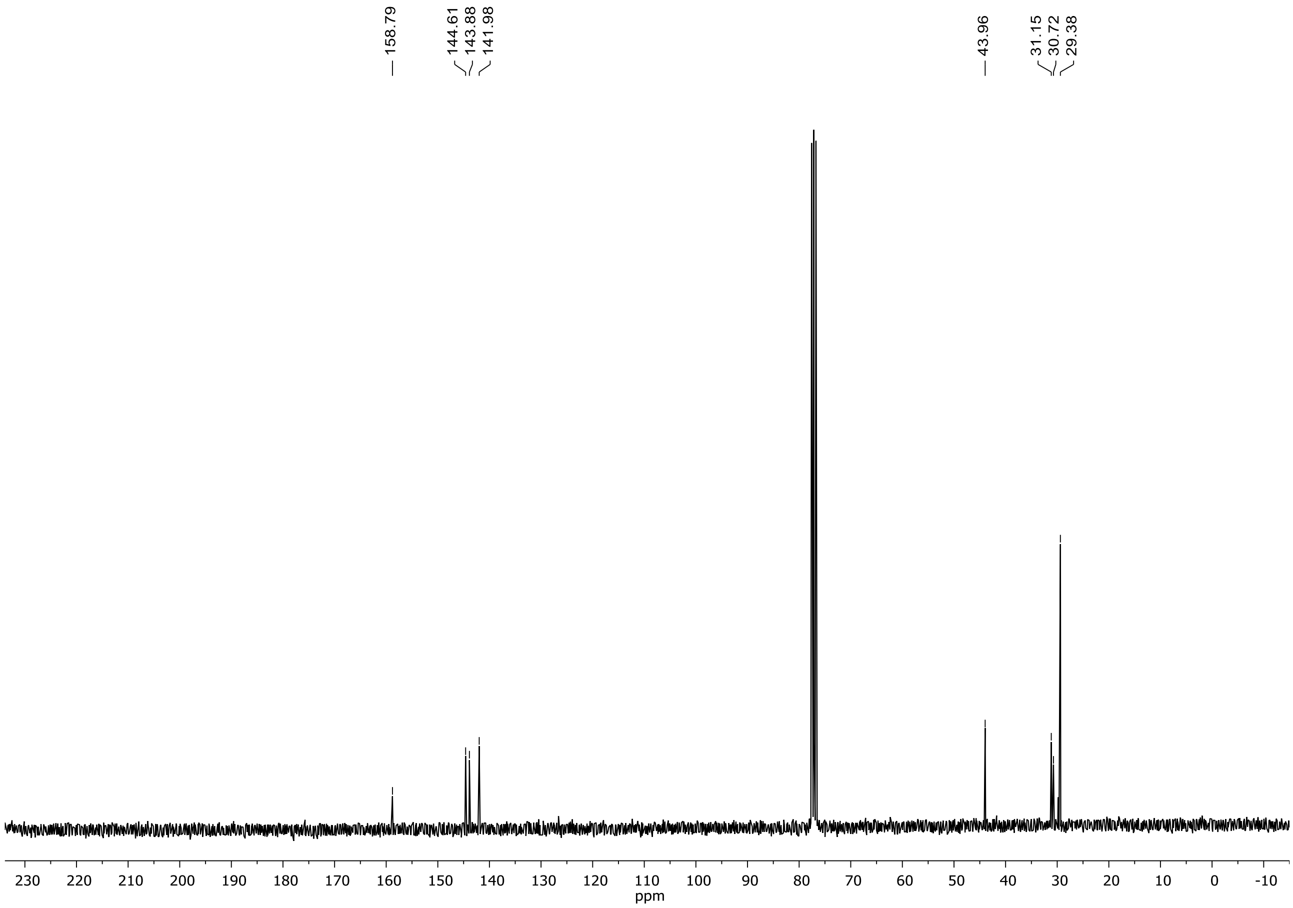

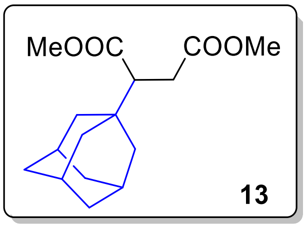

13

^{1}H NMR (300 MHz, chloroform-*d*)

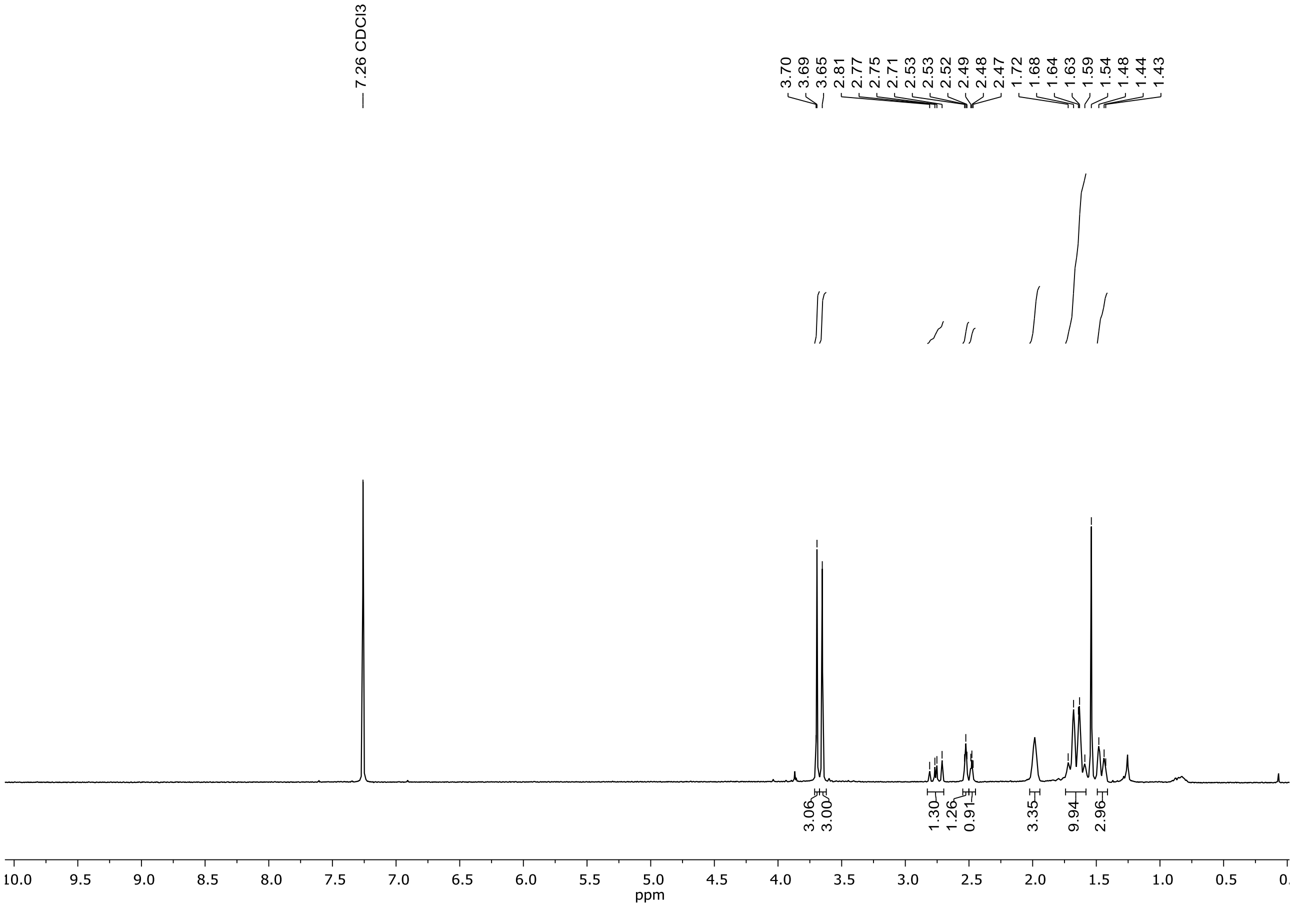

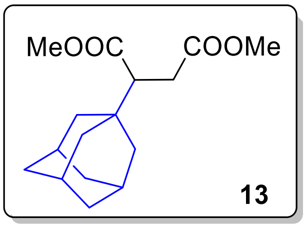

^{13}C NMR (75 MHz, chloroform-*d*)

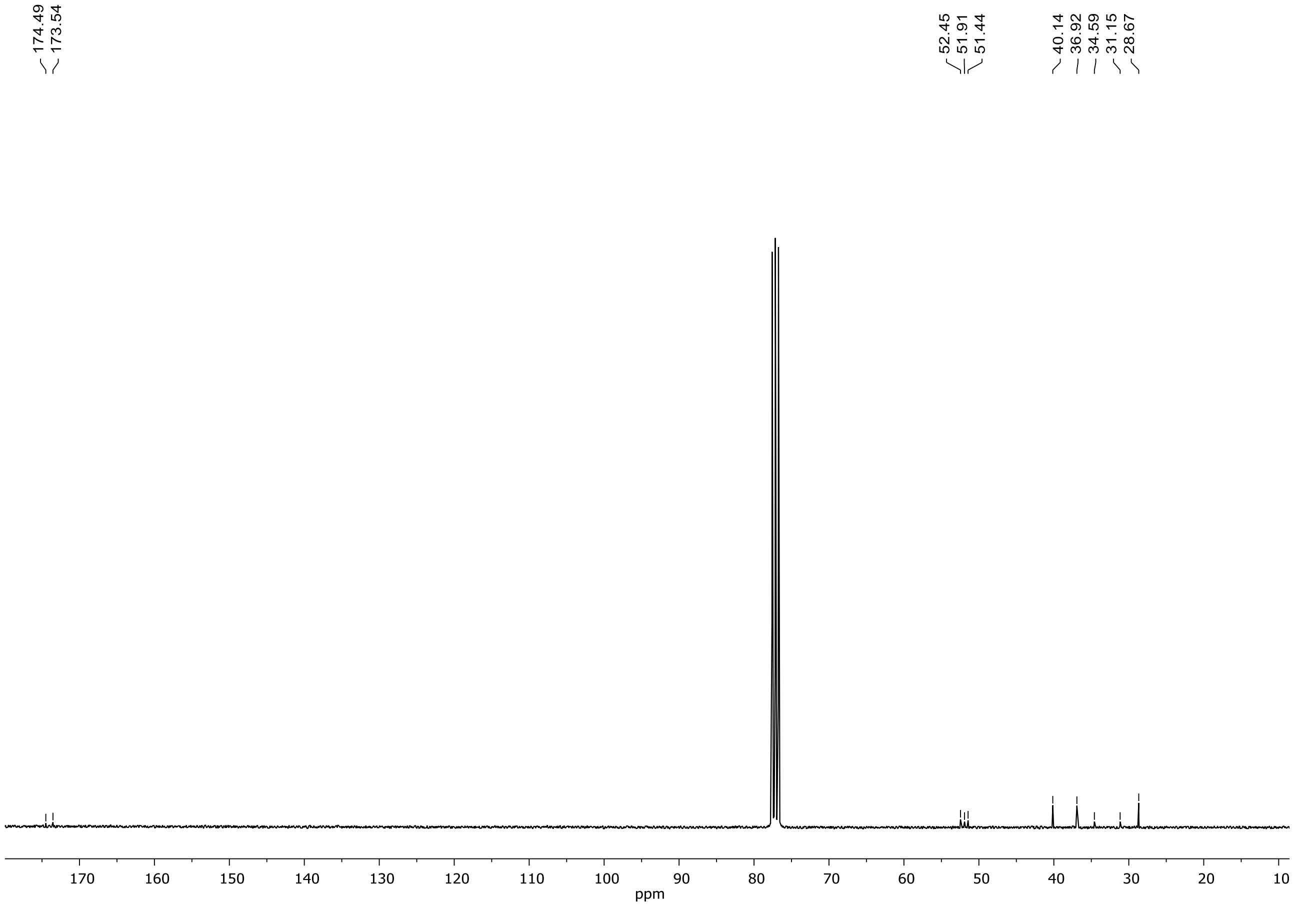

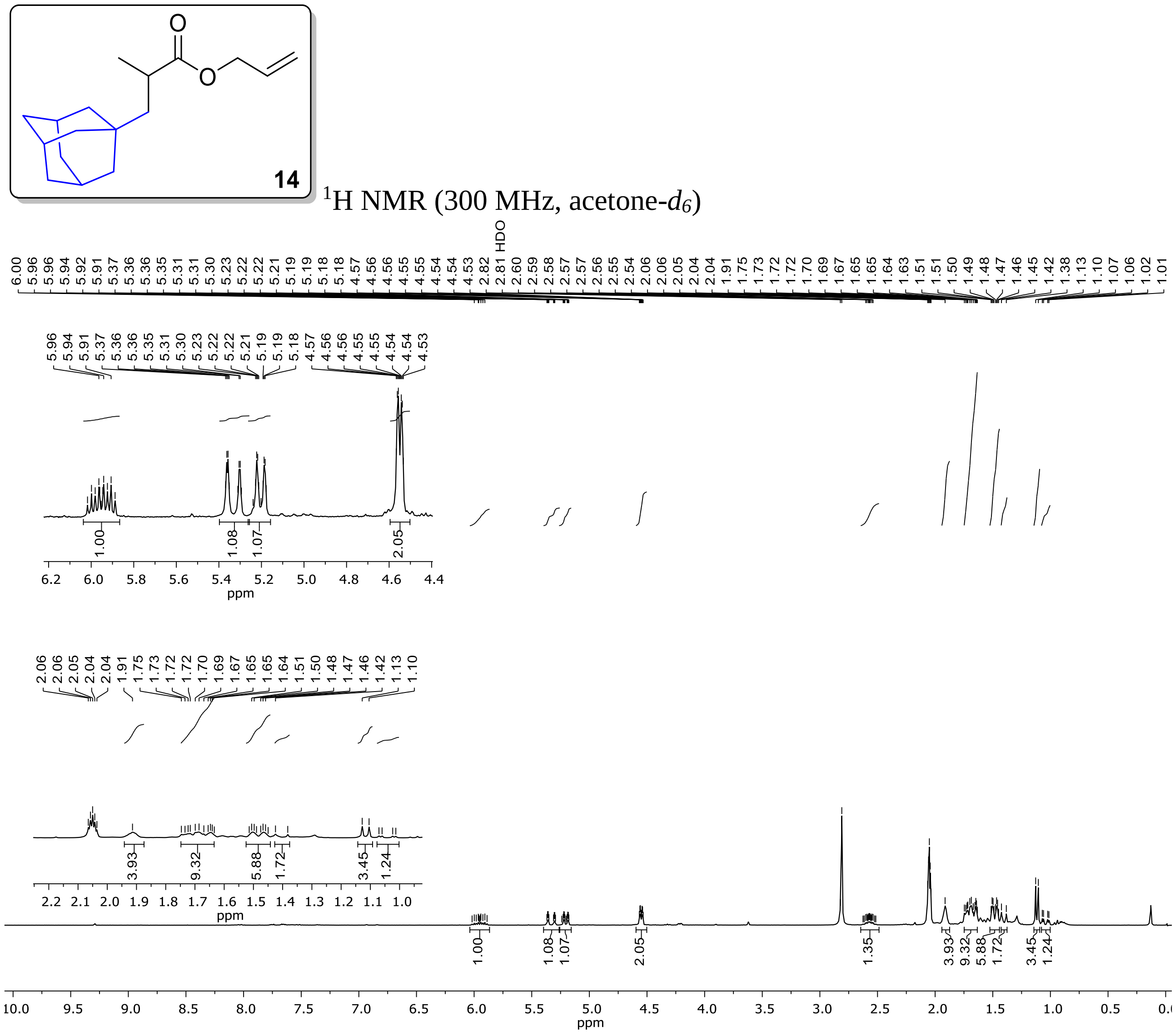

^{1}H NMR (300 MHz, acetone-d_6)

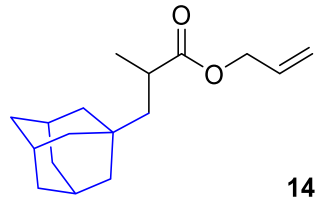

14

^{13}C NMR (75 MHz, acetone-d_6)

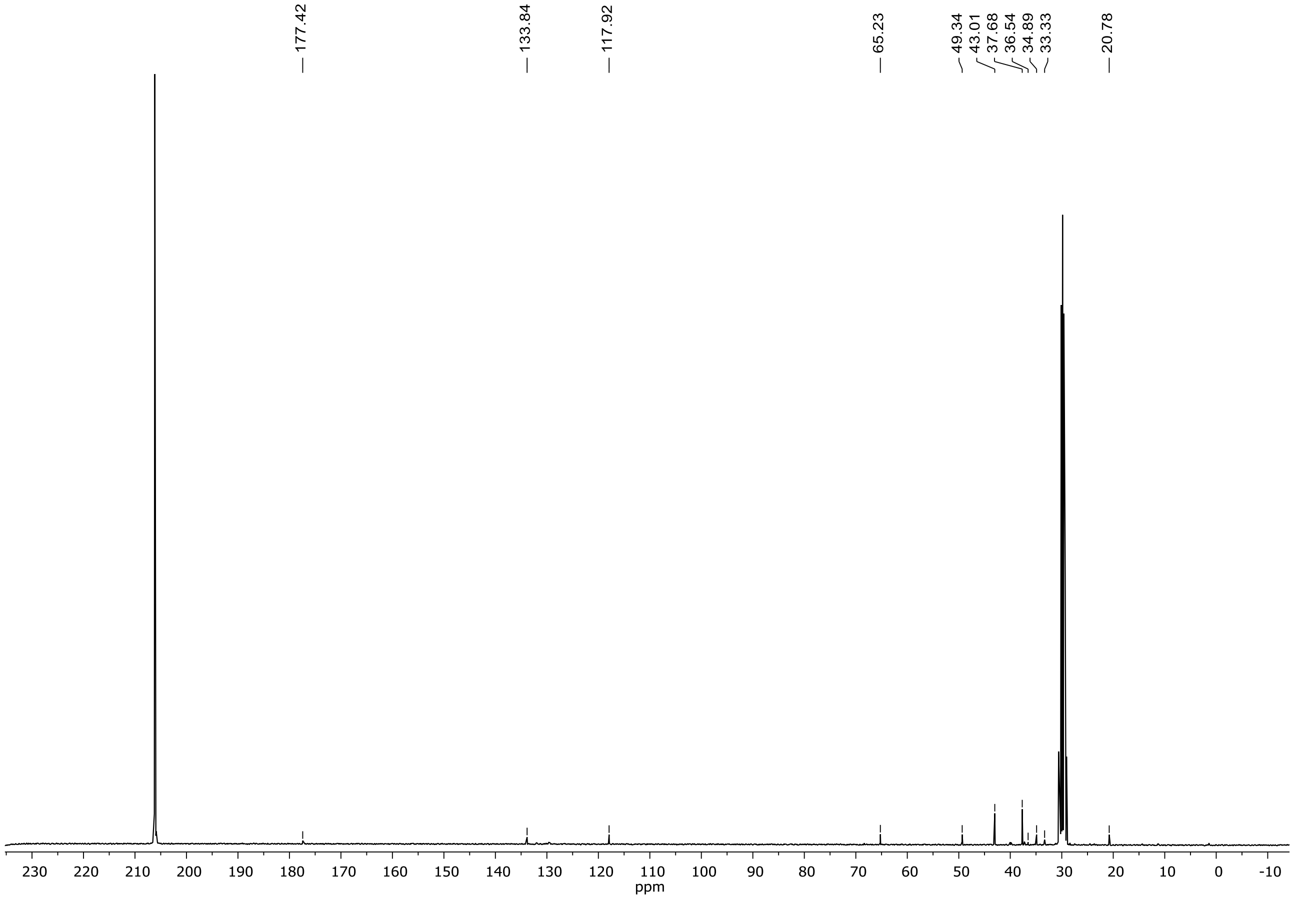

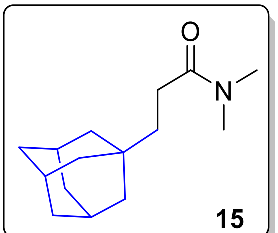

15

^{1}H NMR (300 MHz, chloroform-d)

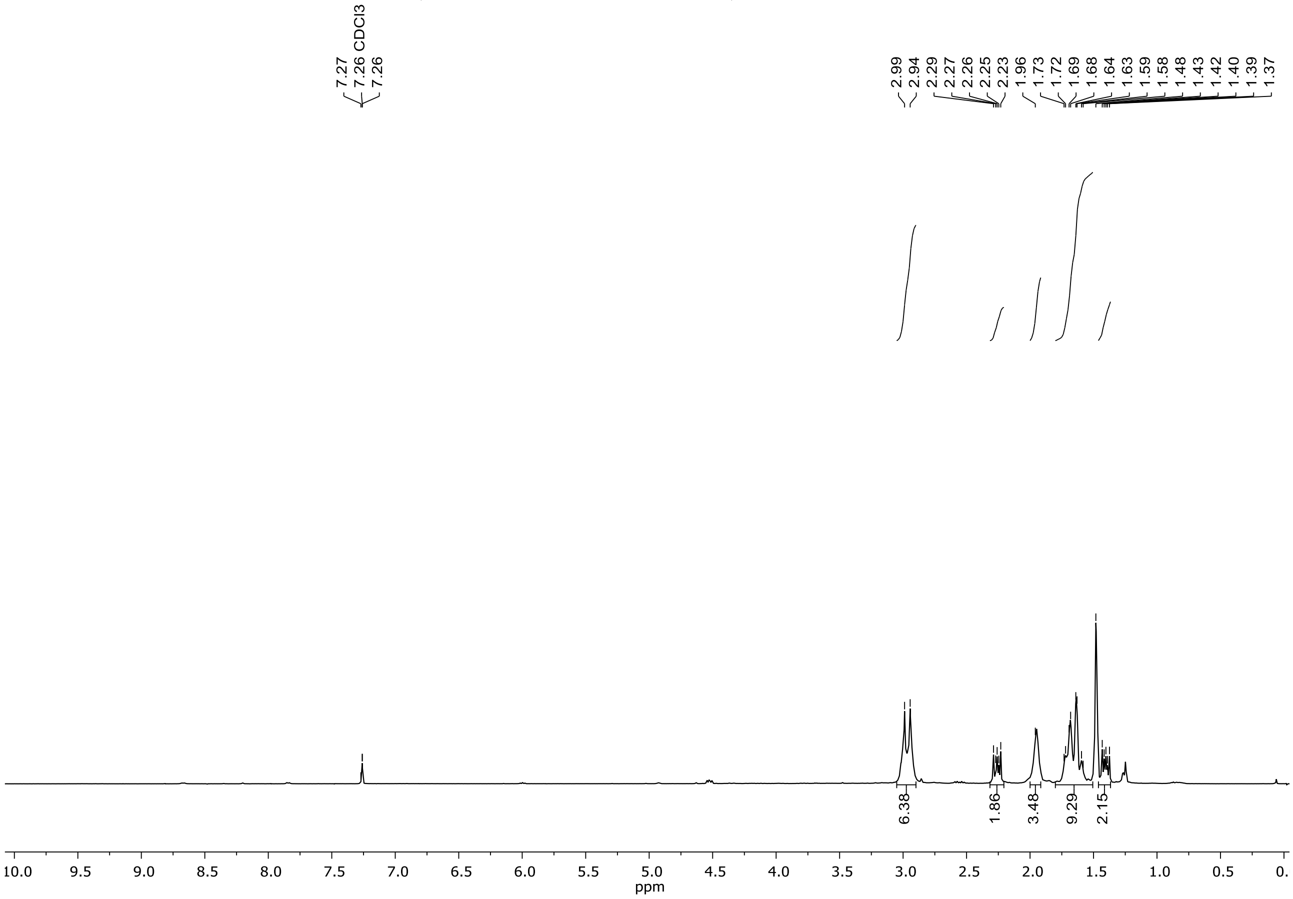

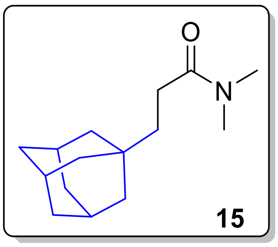

15

^{13}C NMR (75 MHz, chloroform-d)

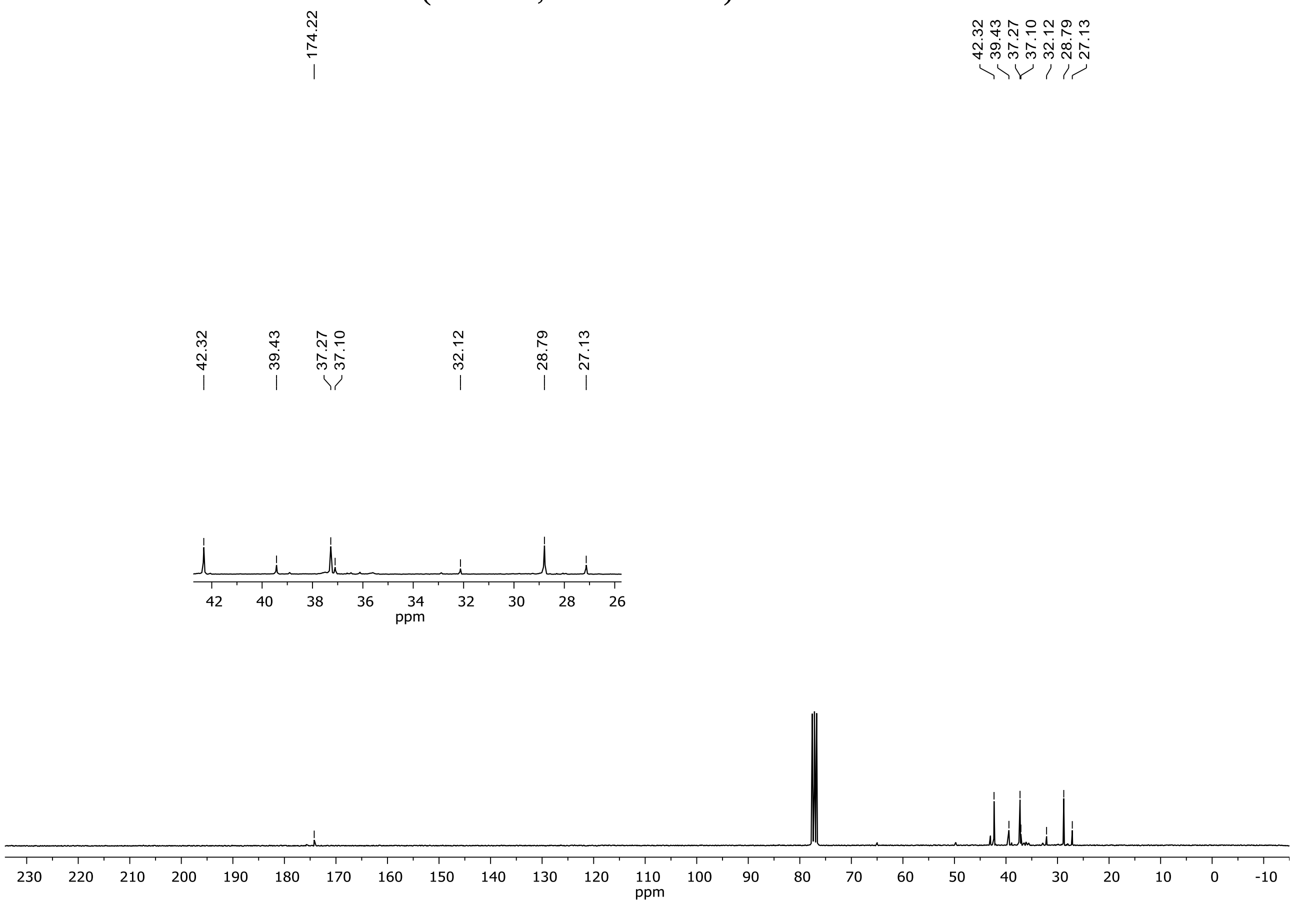

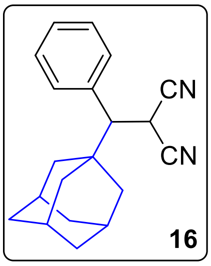

16

^{1}H NMR (300 MHz, chloroform-*d*)

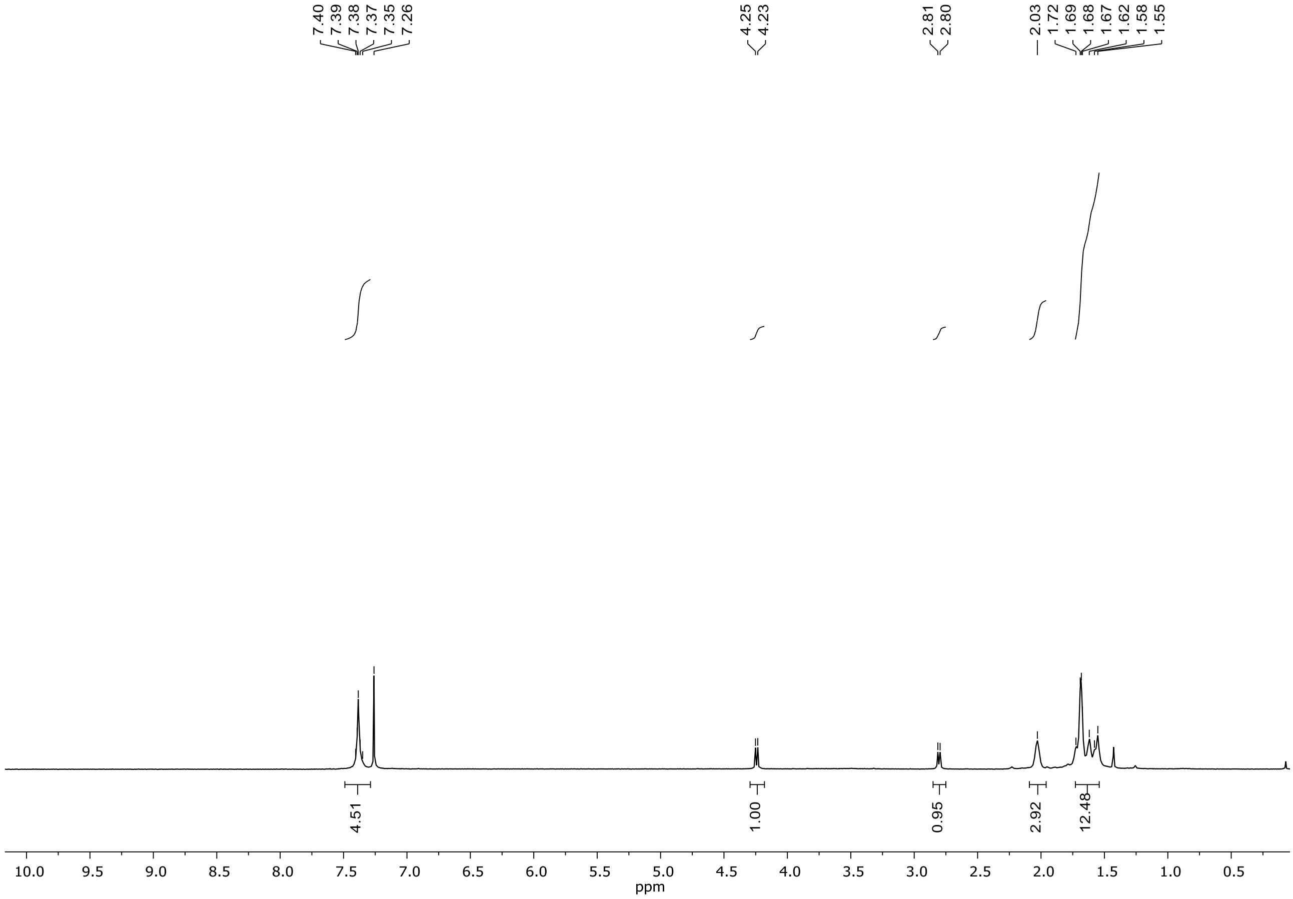

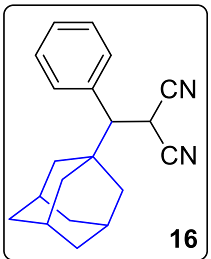

16 ^{13}C NMR (75 MHz, chloroform-*d*)

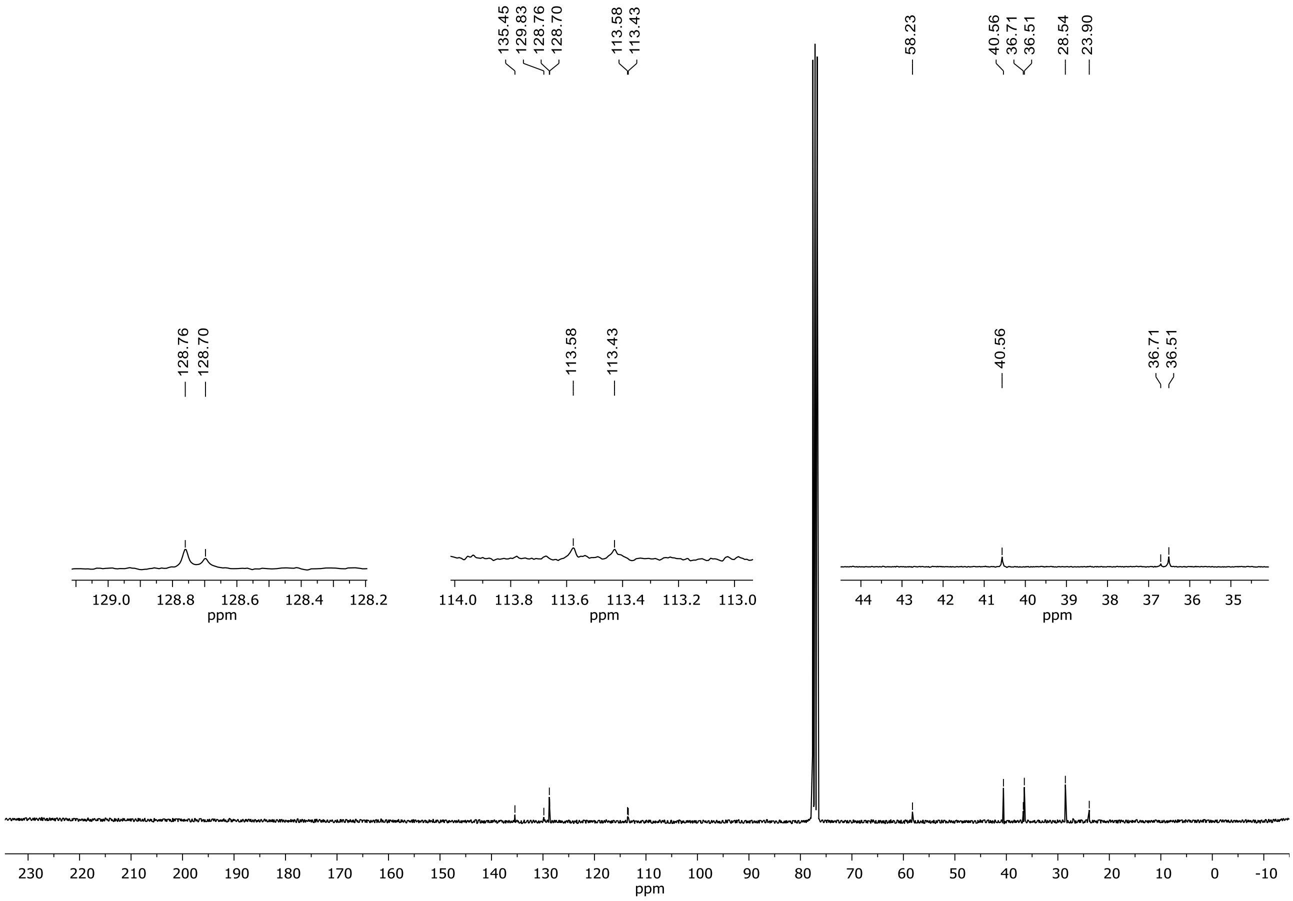

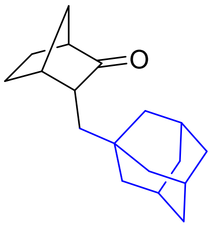

17 ^{1}H NMR (300 MHz, chloroform-*d*)

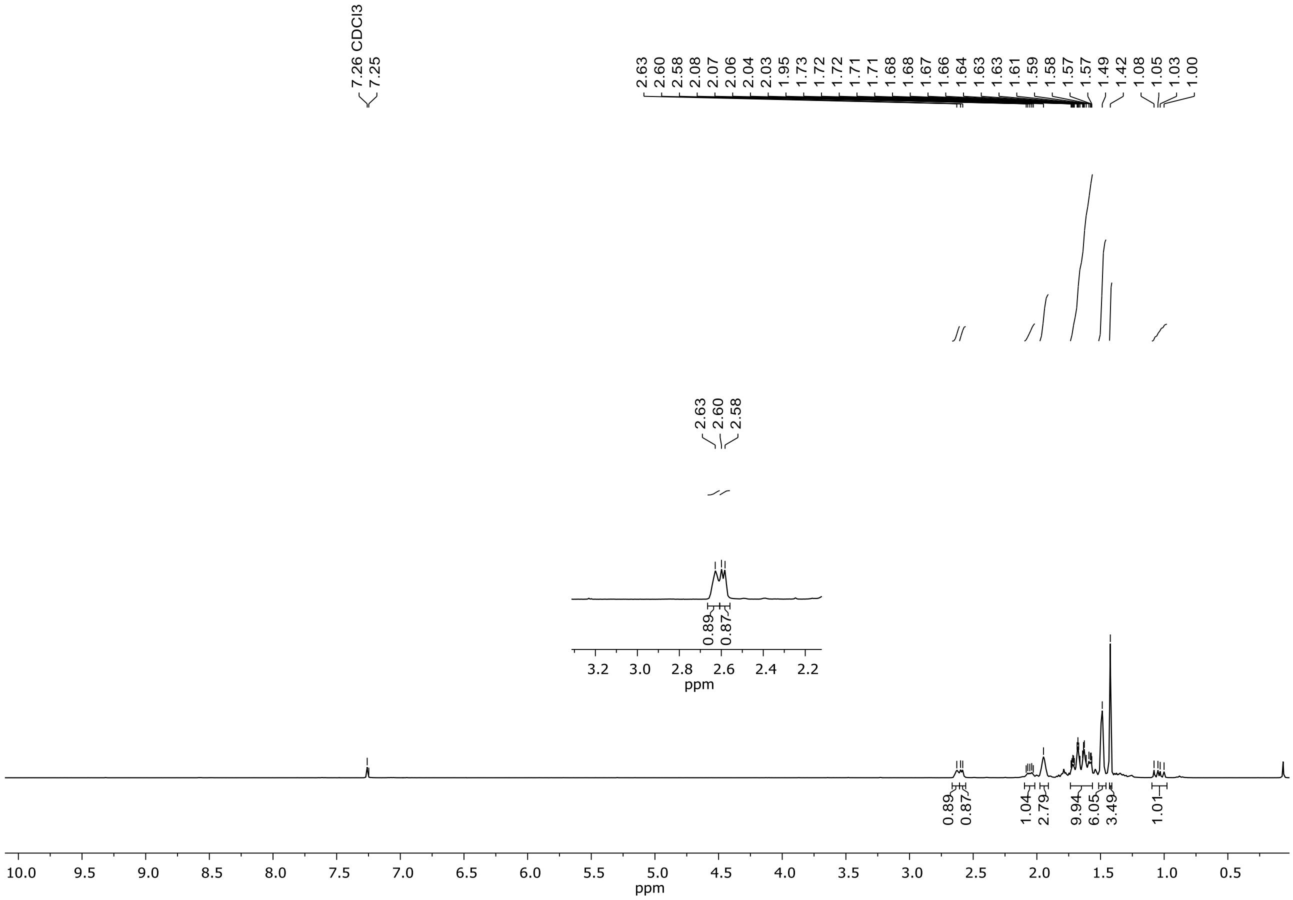

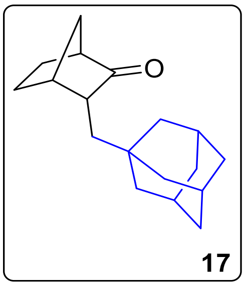

17 ^{13}C NMR (75 MHz, chloroform-*d*)

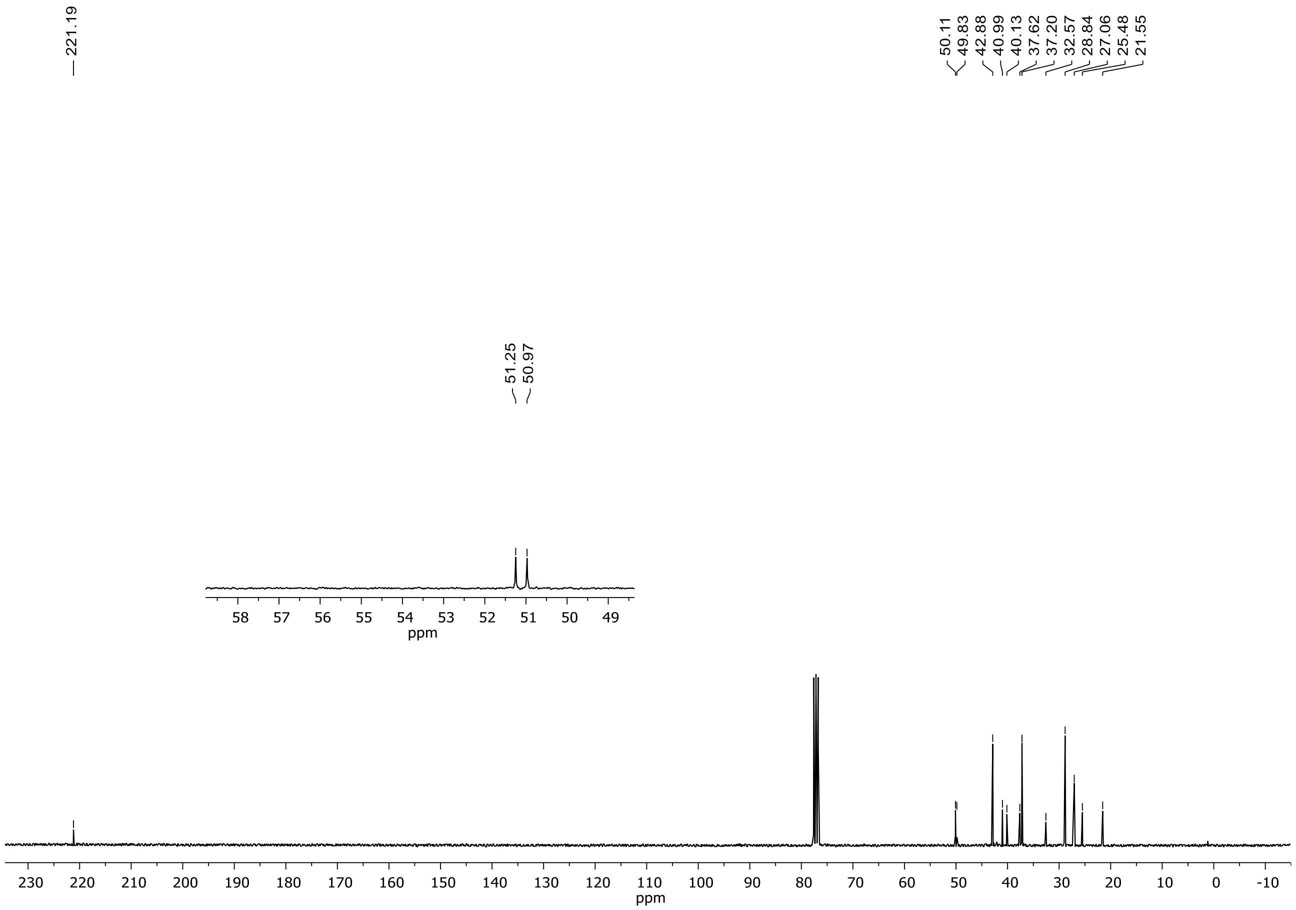

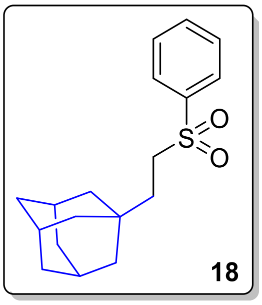

18

^{1}H NMR (300 MHz, chloroform-*d*)

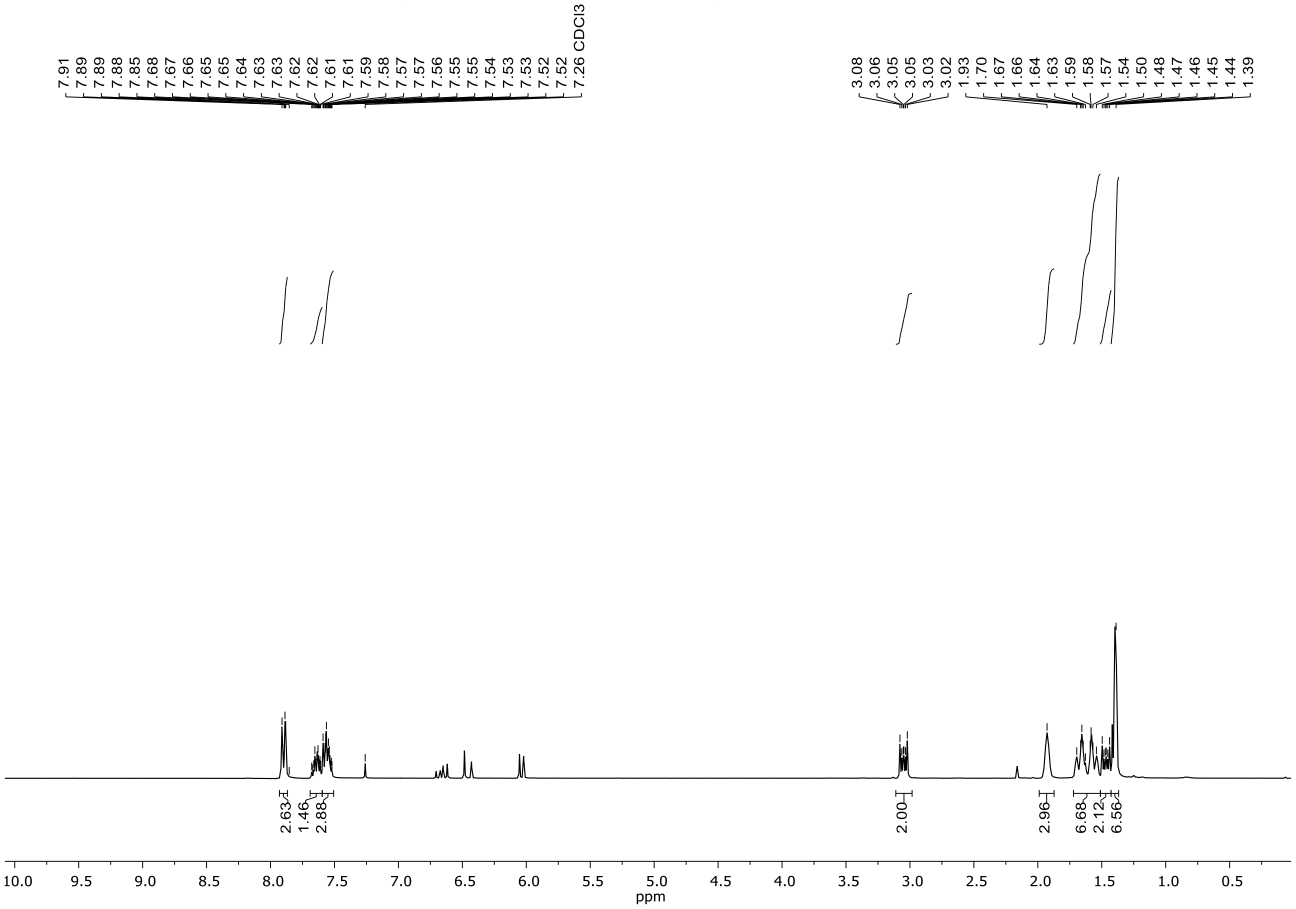

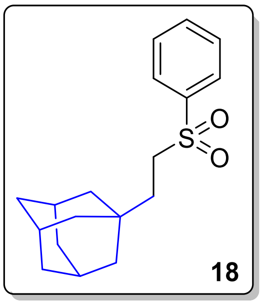

18

^{13}C NMR (75 MHz, chloroform-d)

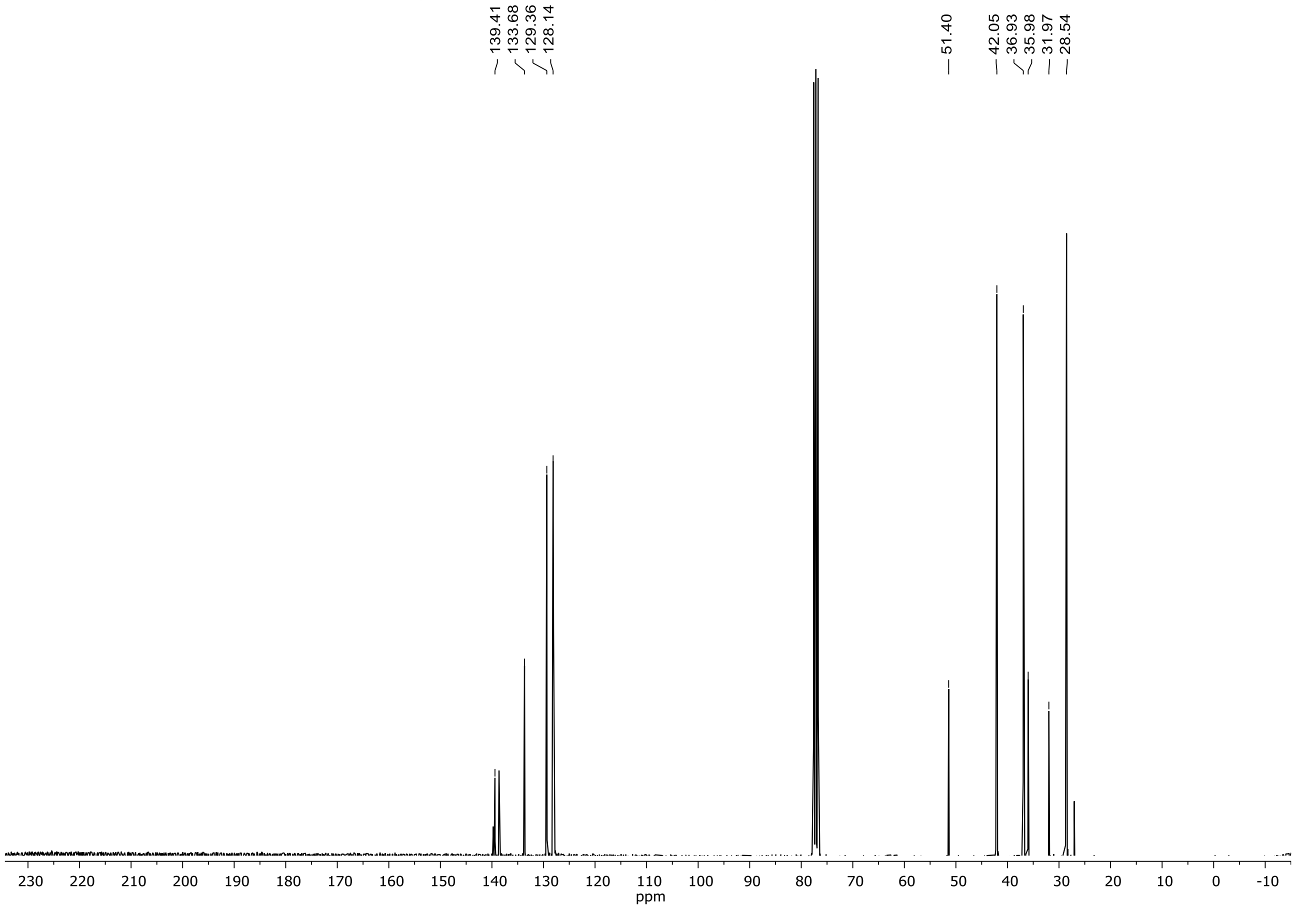

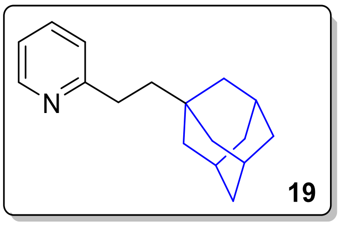

19

^{1}H NMR (300 MHz, chloroform-*d*)

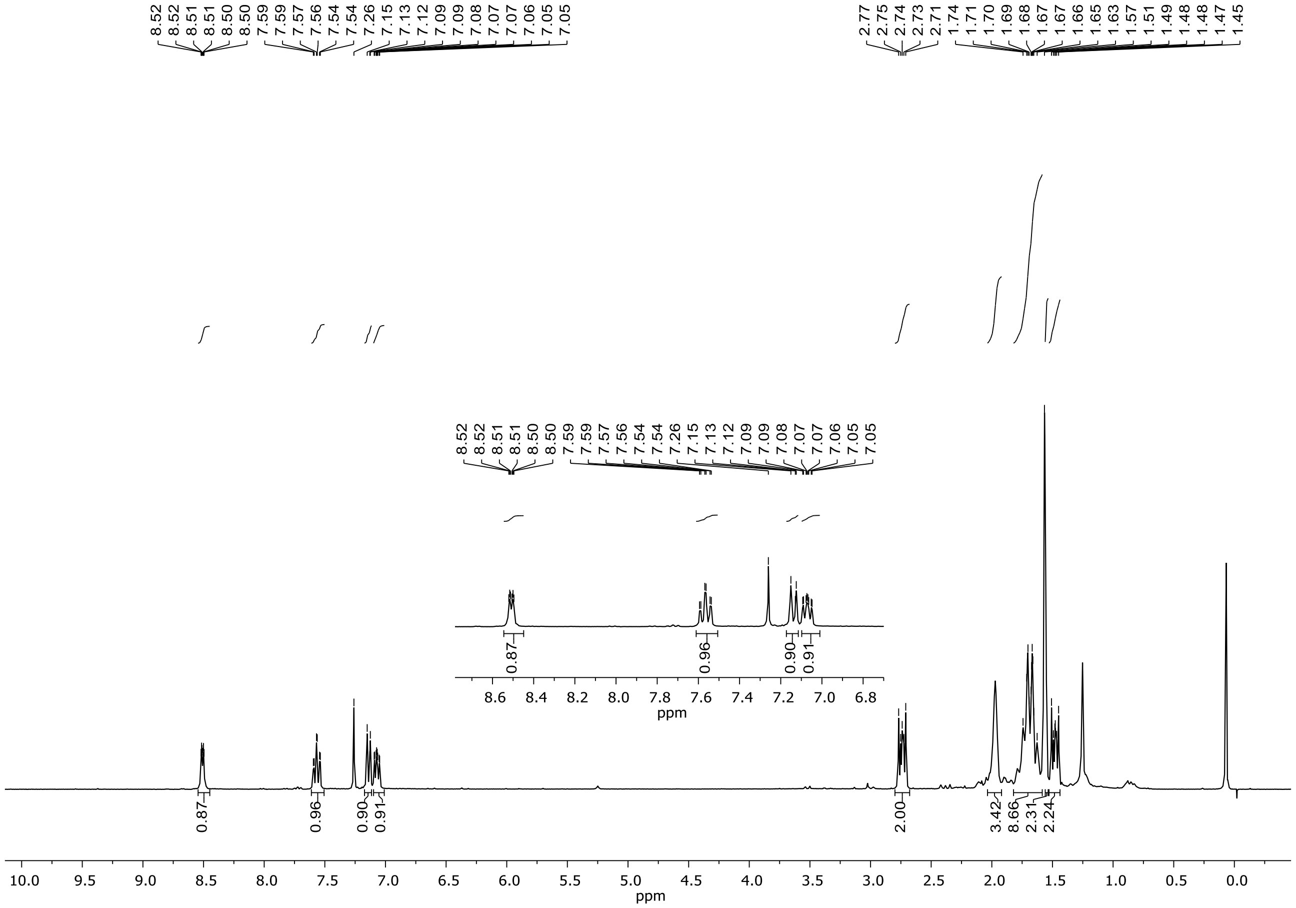

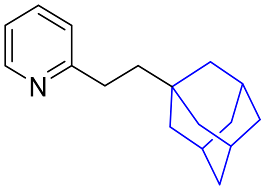

19

^{13}C NMR (75 MHz, chloroform-*d*)

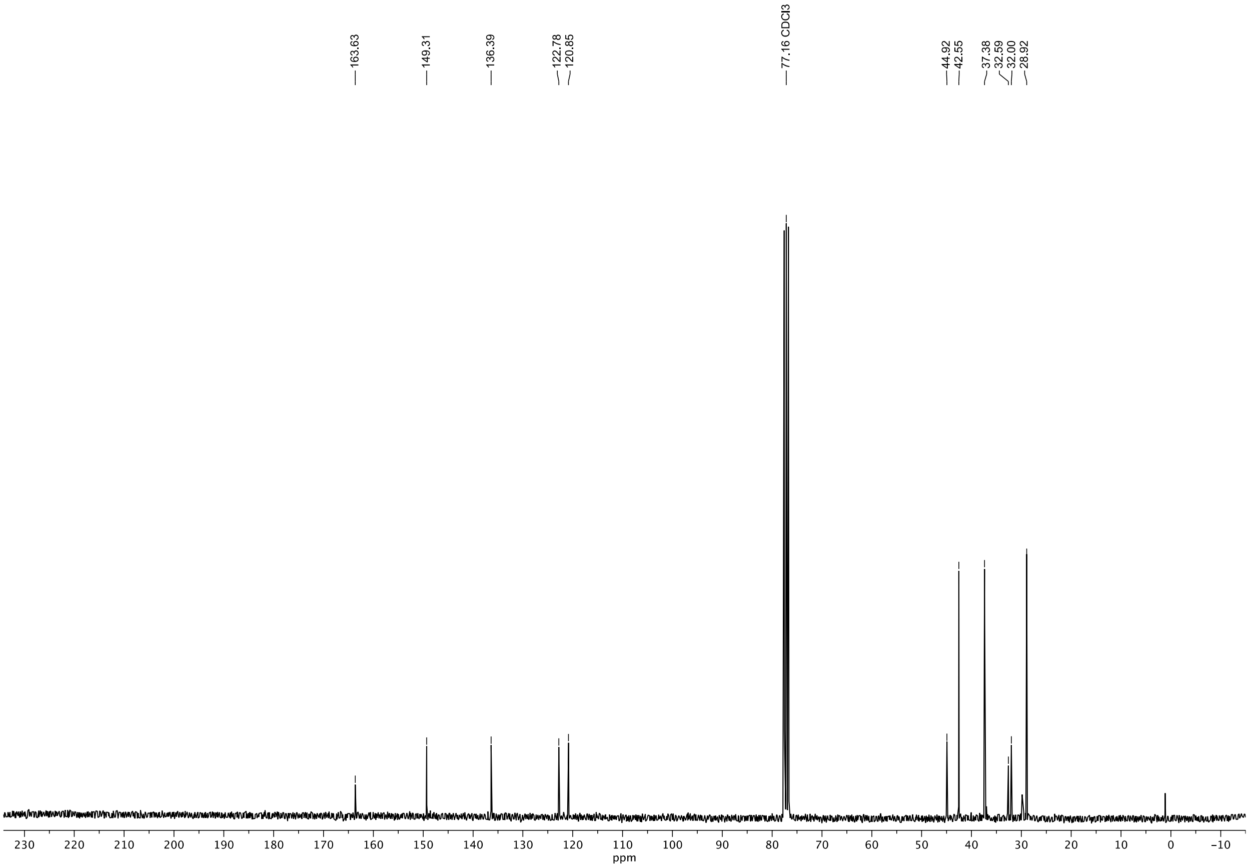

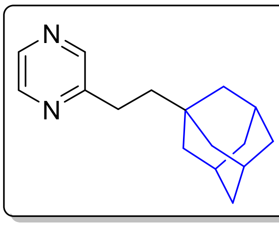

20

^{1}H NMR (300 MHz, chloroform-d)

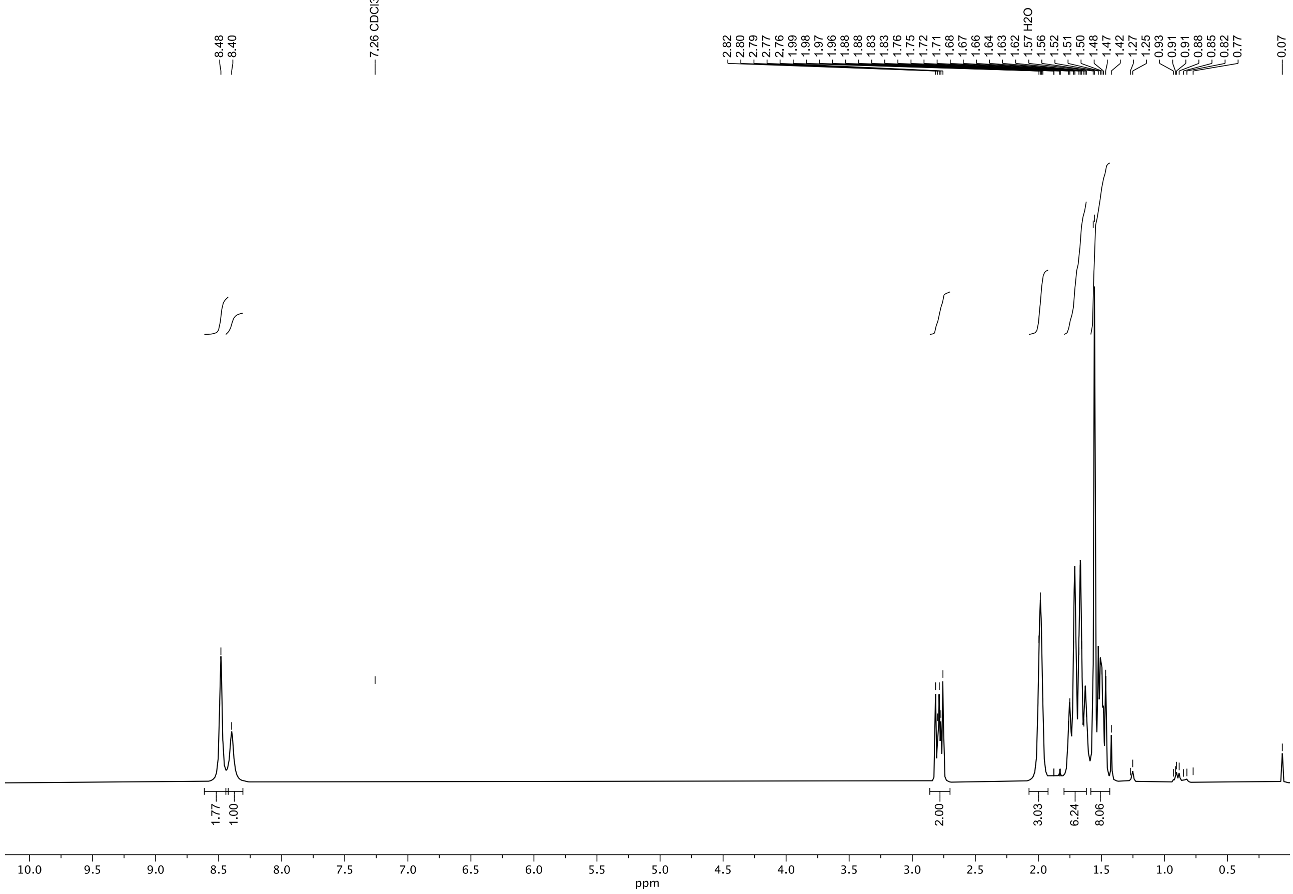

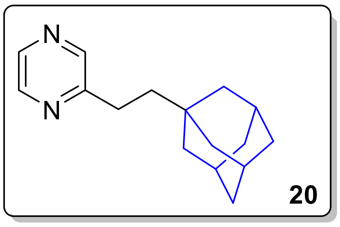

^{13}C NMR (75 MHz, chloroform-*d*)

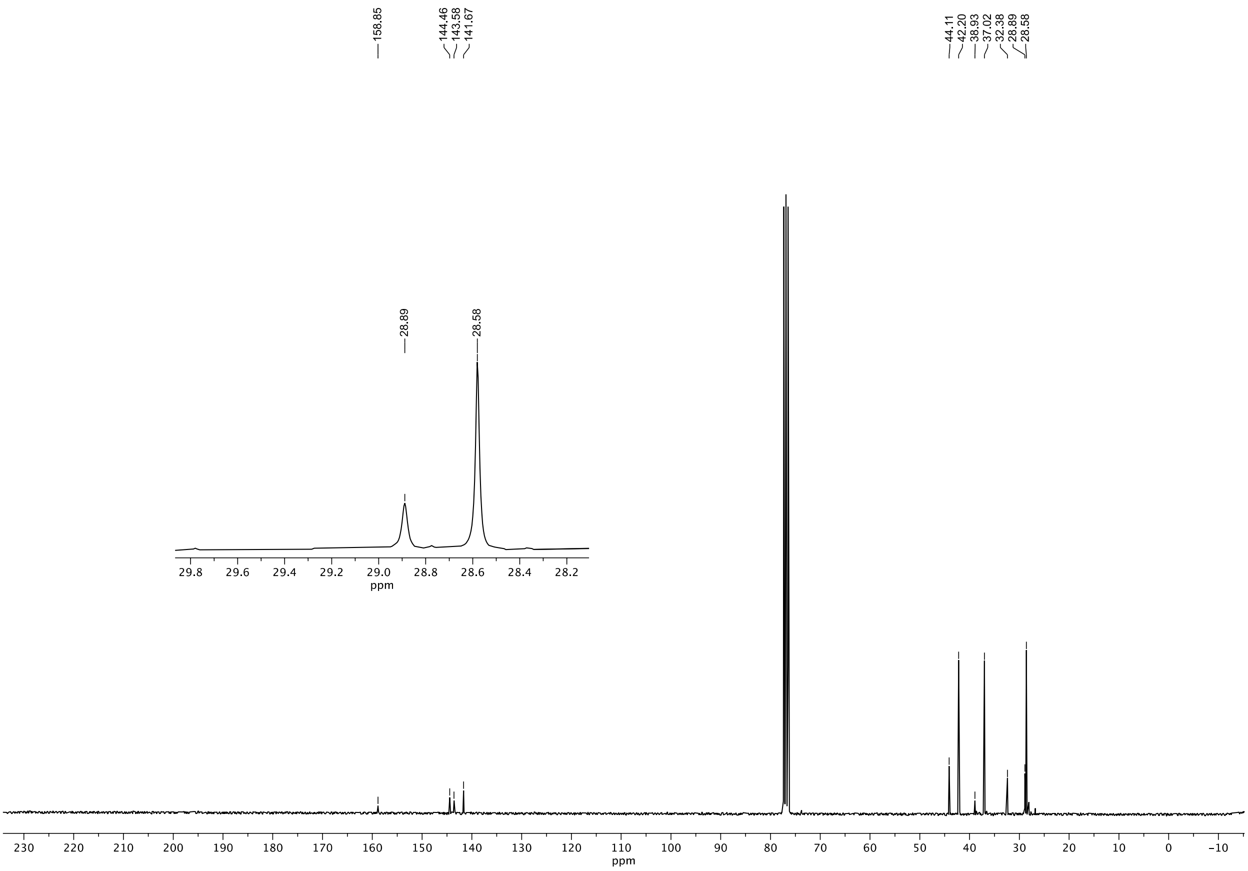

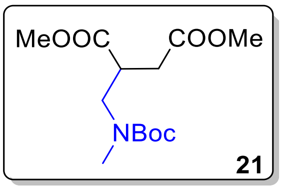

21

^{1}H NMR (300 MHz, chloroform-d)

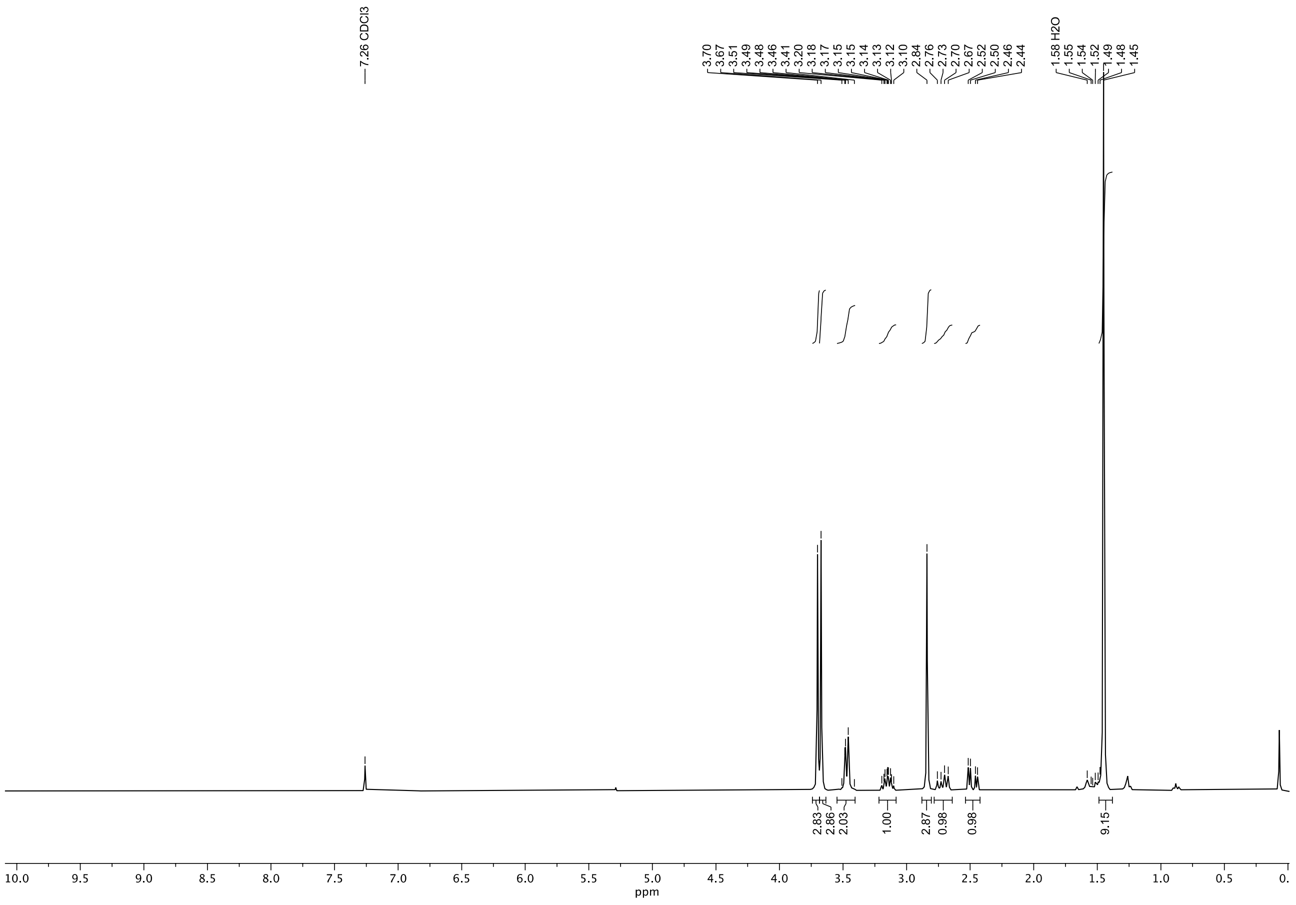

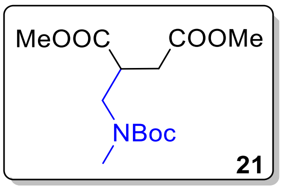

^{13}C NMR (75 MHz, chloroform-*d*)

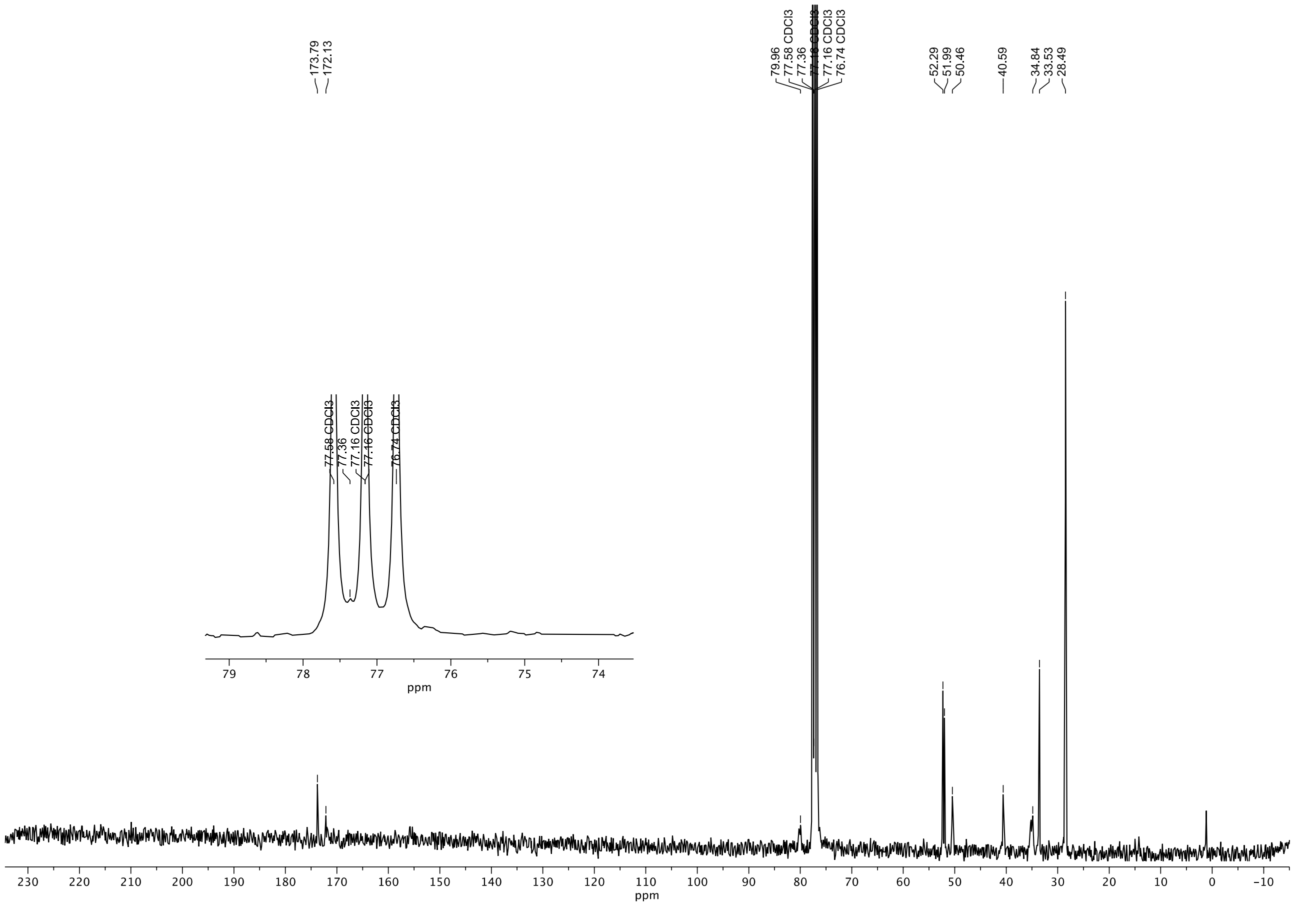

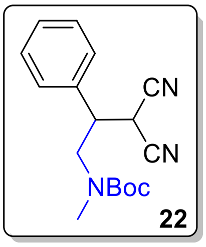

^{1}H NMR (300 MHz, chloroform-*d*)

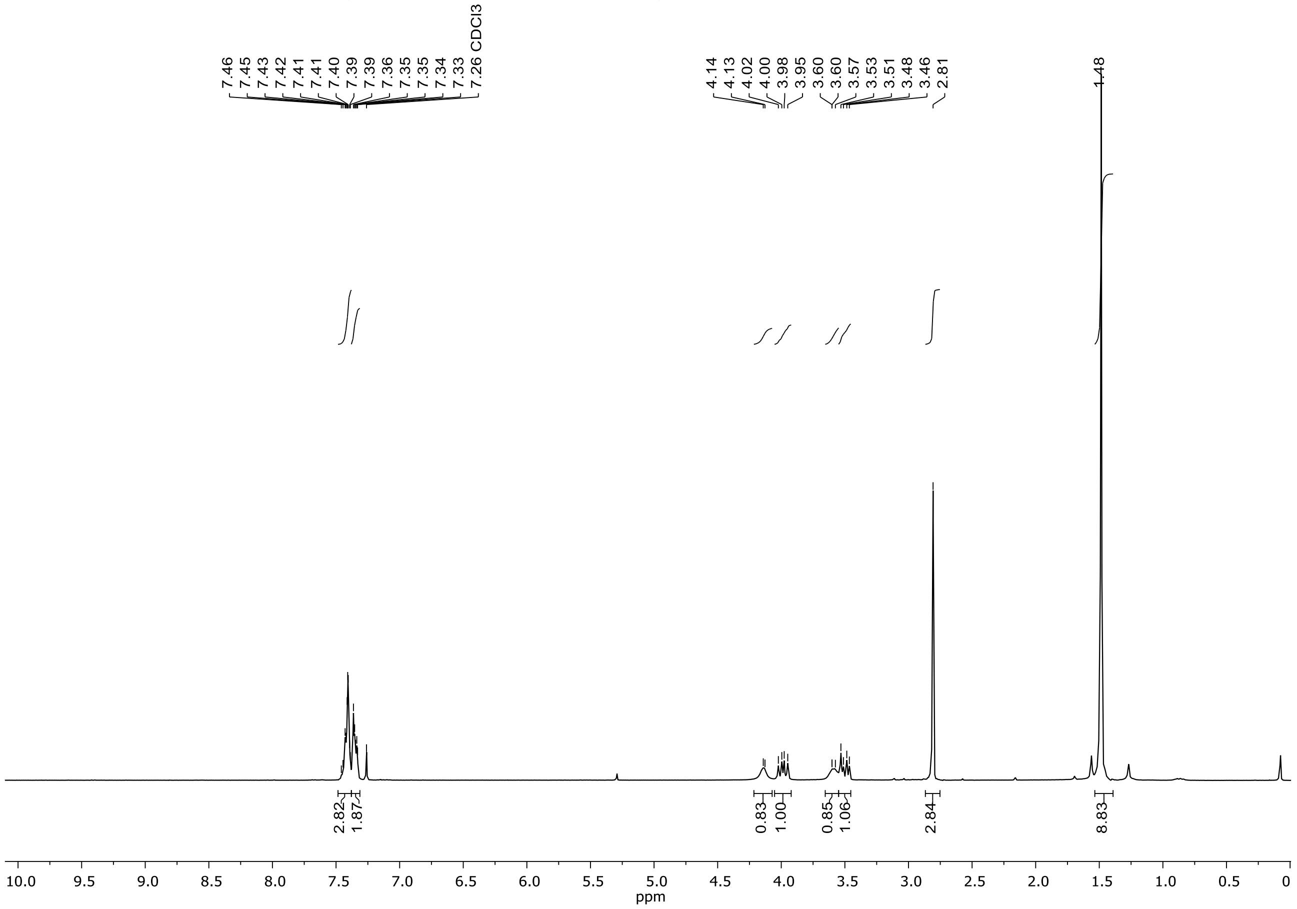

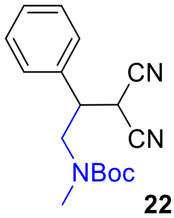

22

^{13}C NMR (75 MHz, chloroform-d)

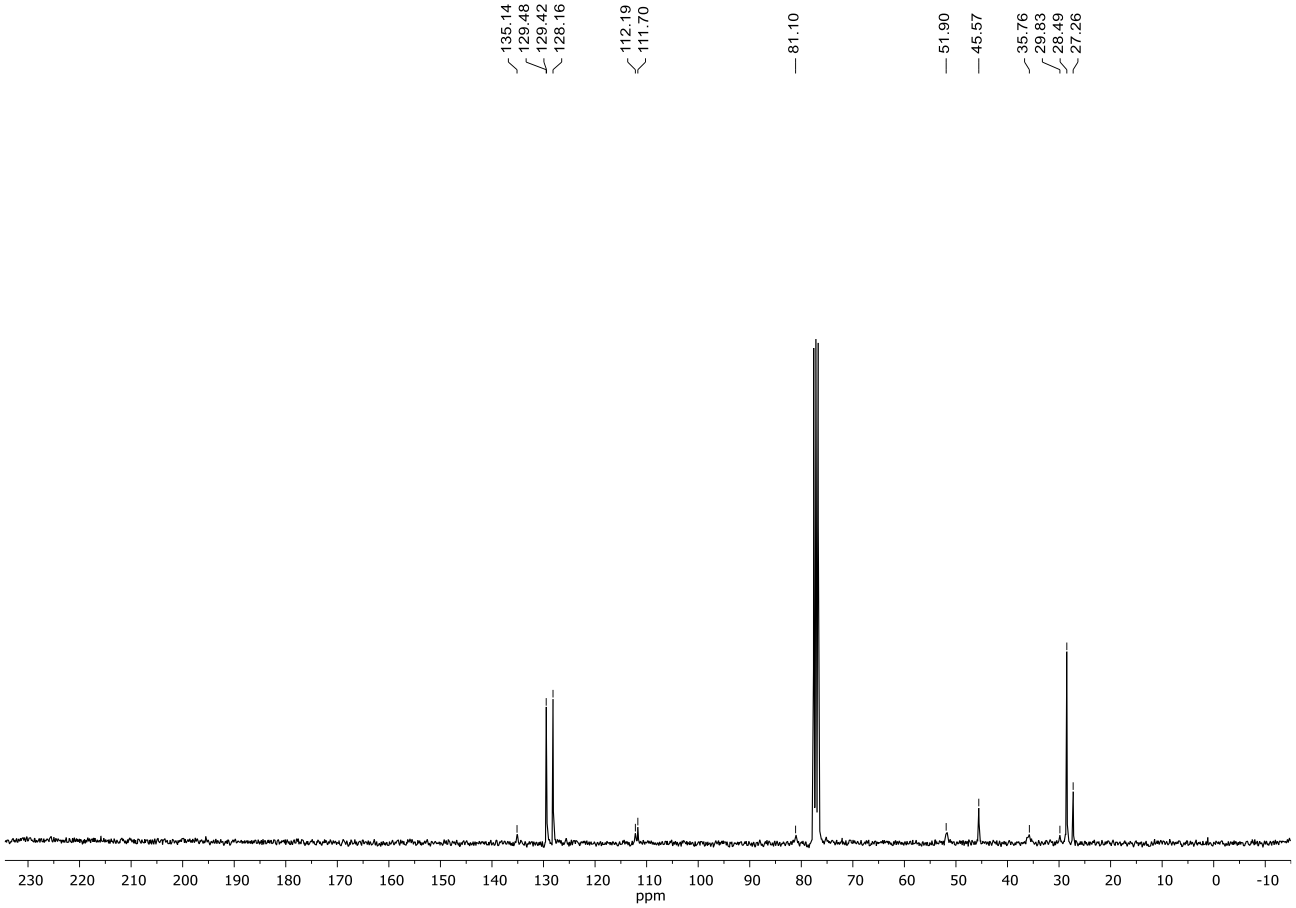

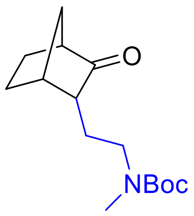

23

^{1}H NMR (300 MHz, chloroform-*d*)

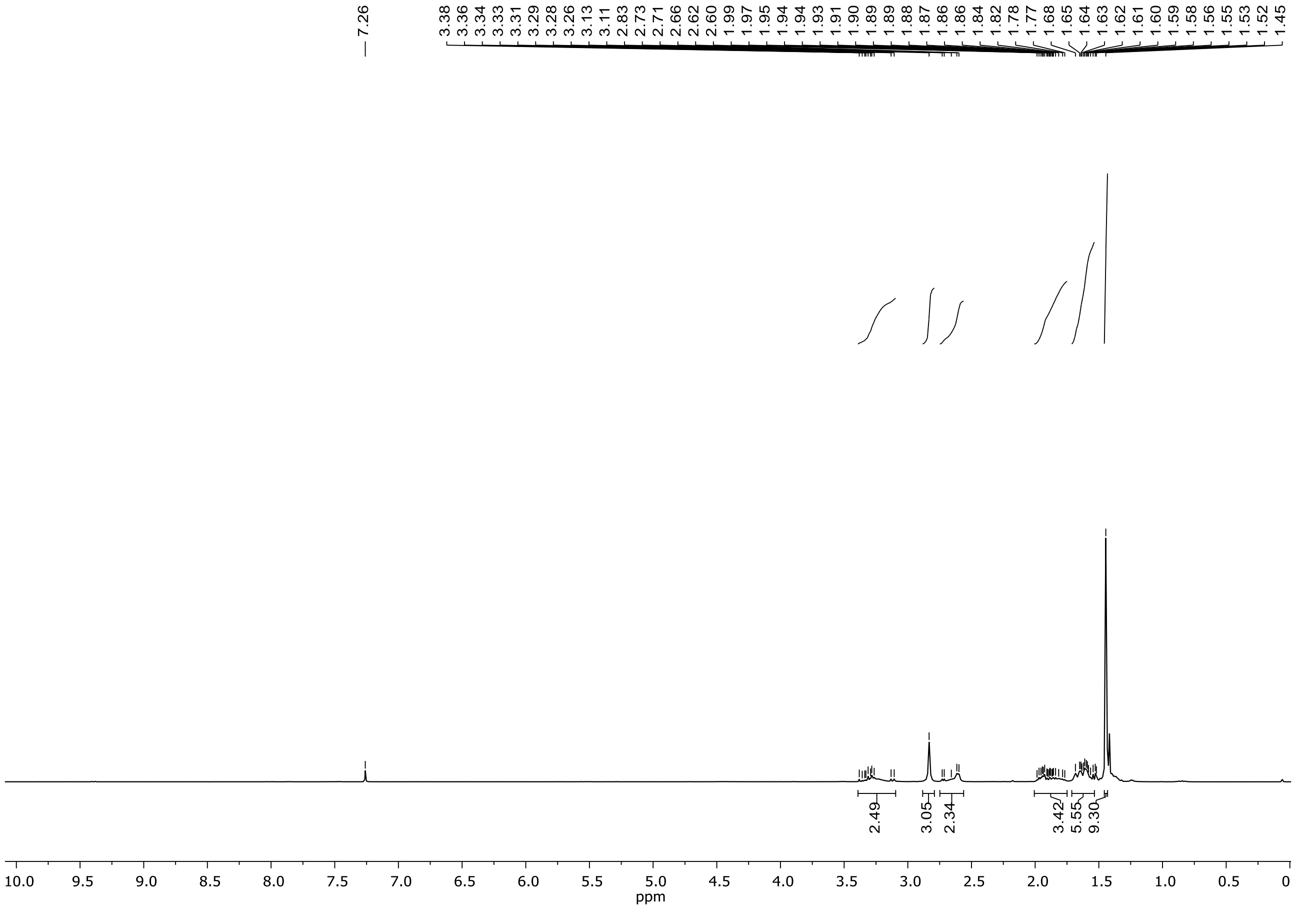

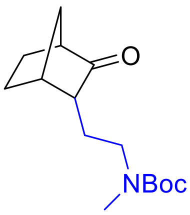

23

^{13}C NMR (75 MHz, chloroform-*d*)

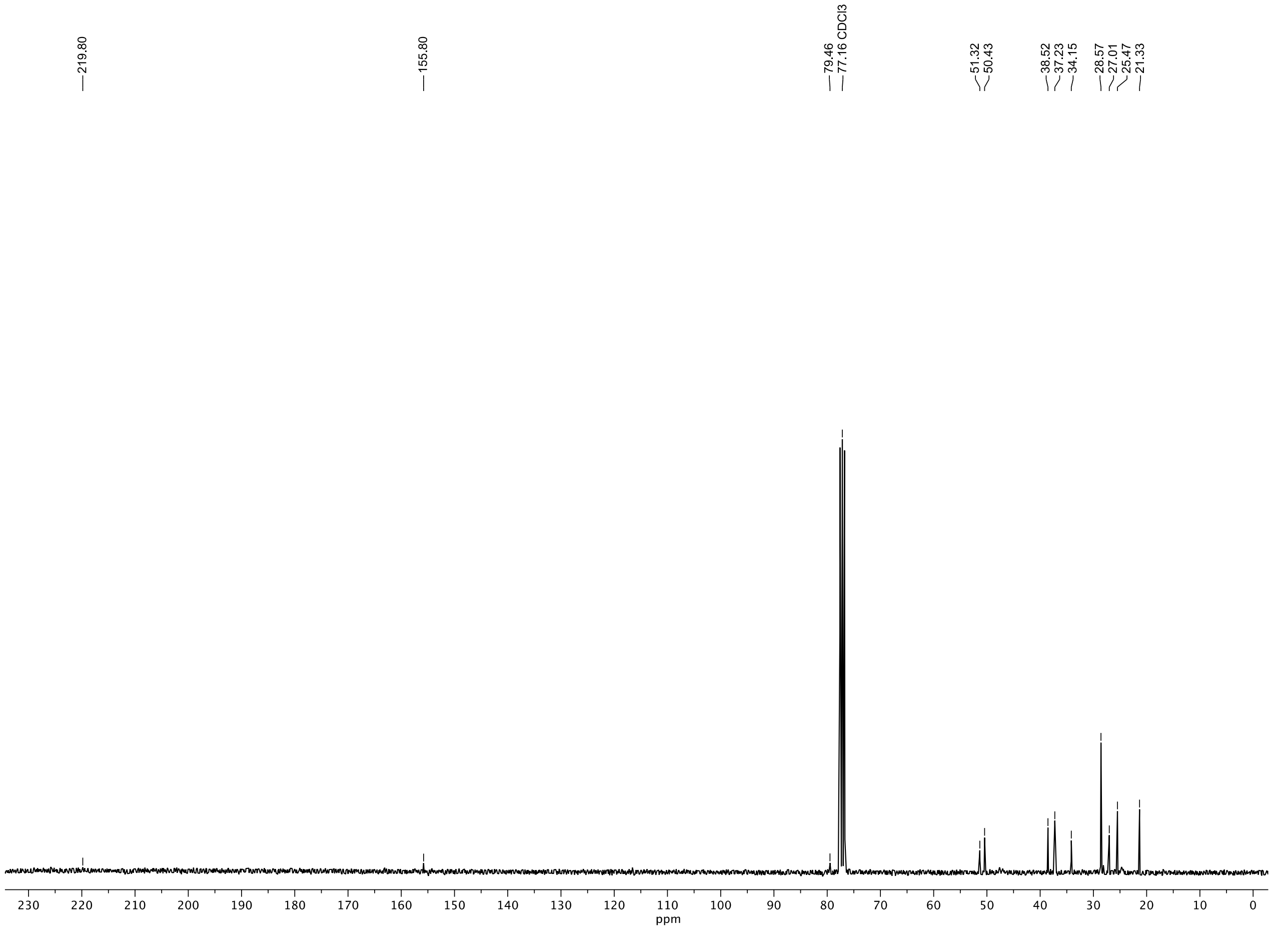

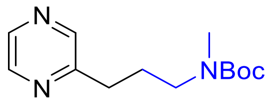

24

^{1}H NMR (300 MHz, chloroform-*d*)

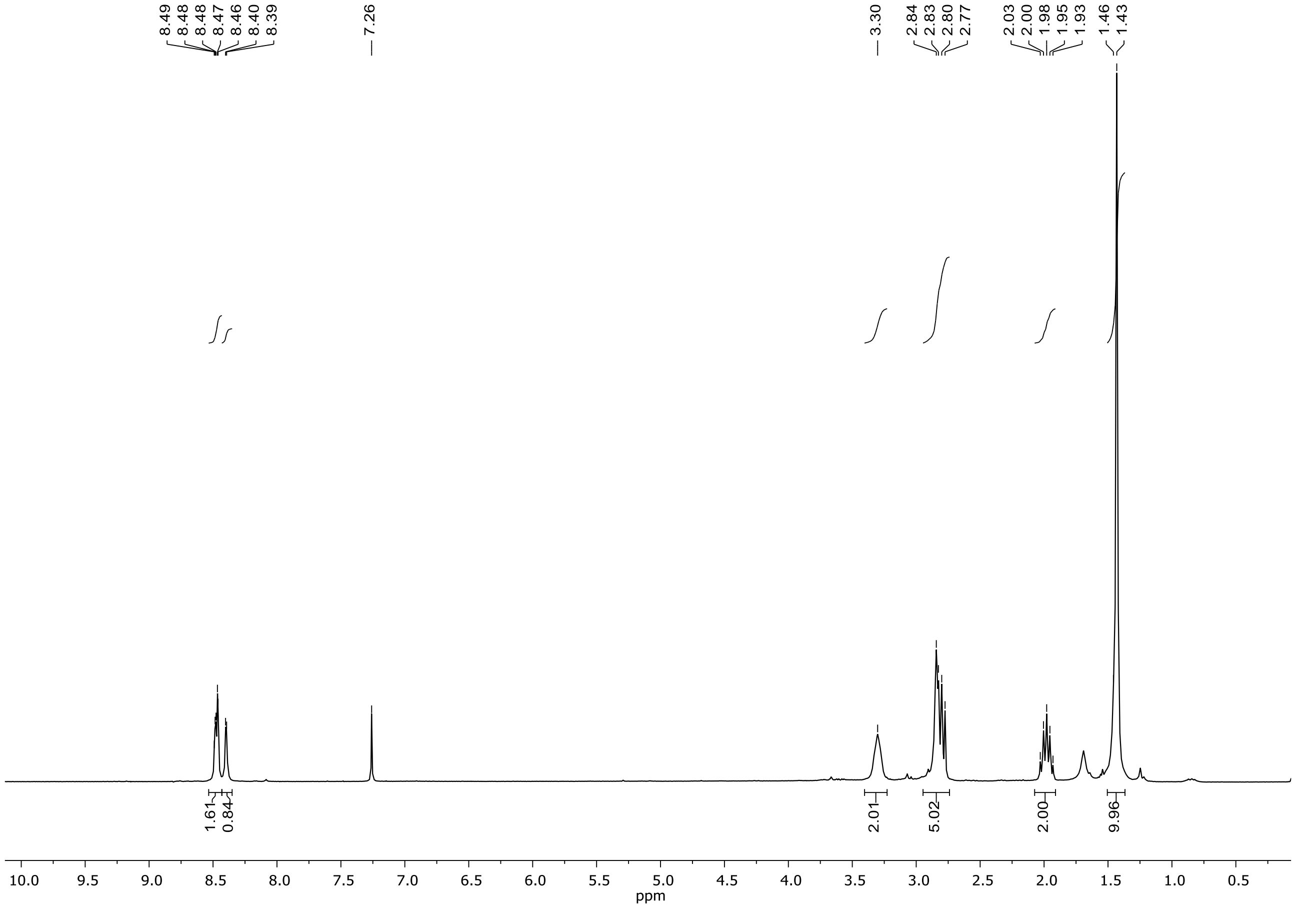

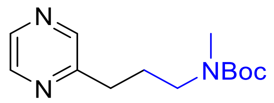

24

^{13}C NMR (75 MHz, chloroform-d)

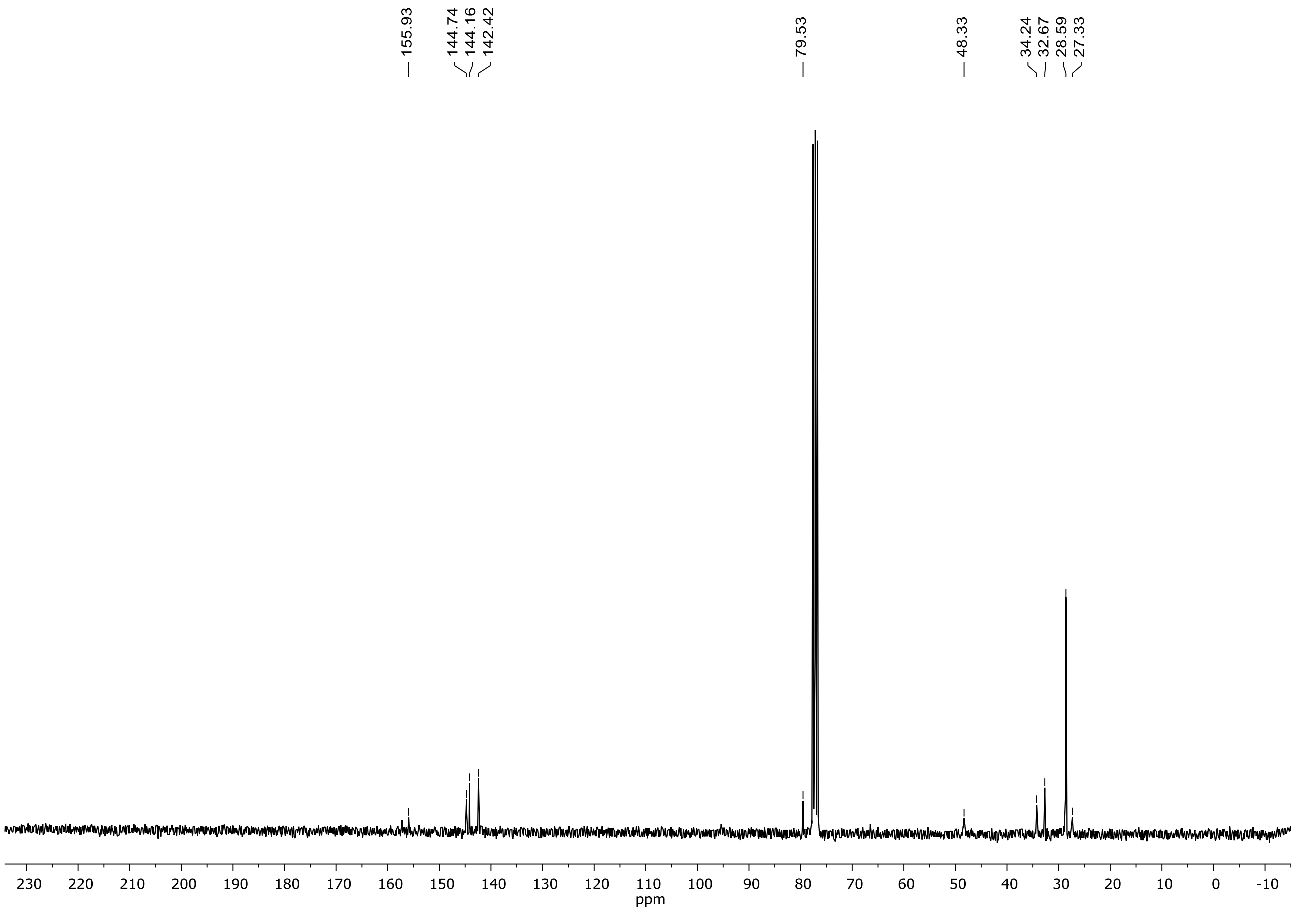

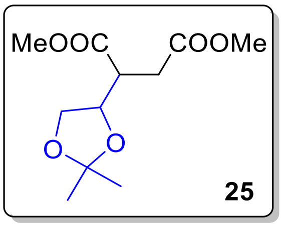

25

^{1}H NMR (300 MHz, chloroform-*d*, mixture of two diasteroisomers)

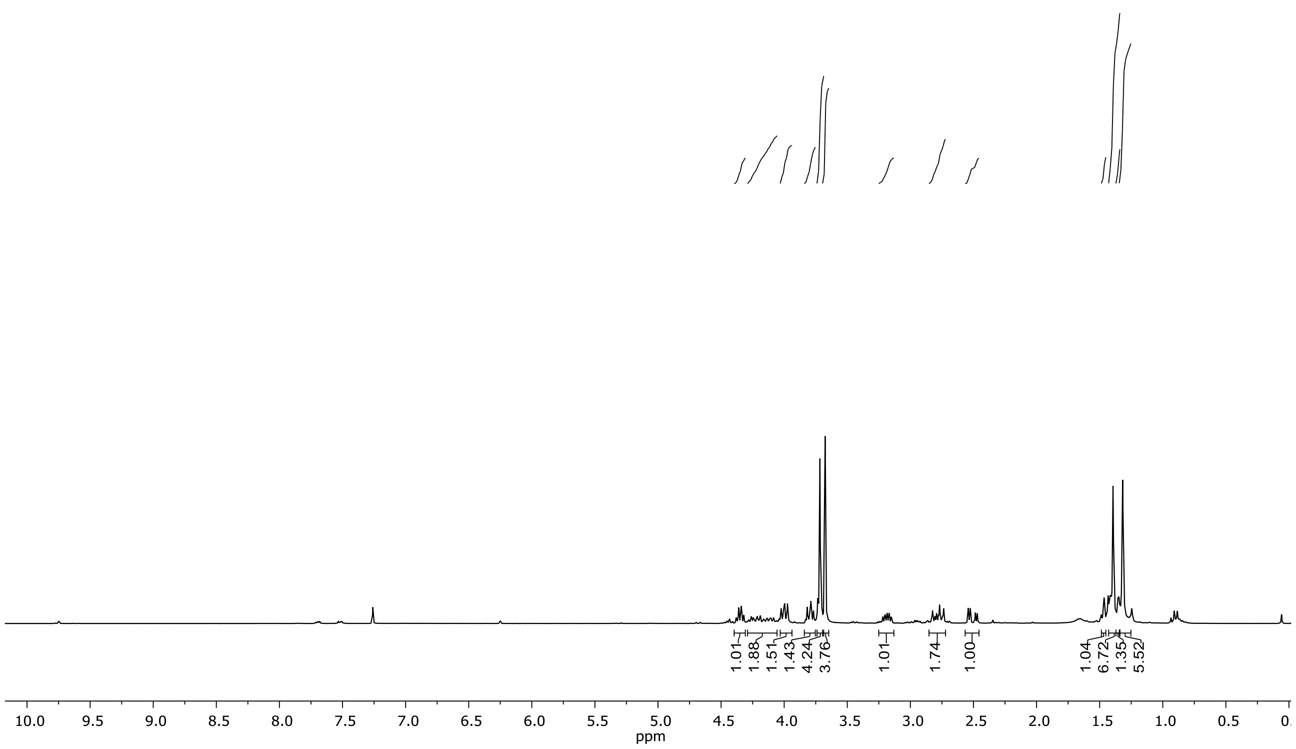

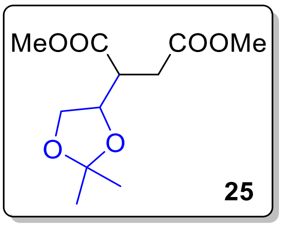

^{13}C NMR (75 MHz, chloroform-*d*)

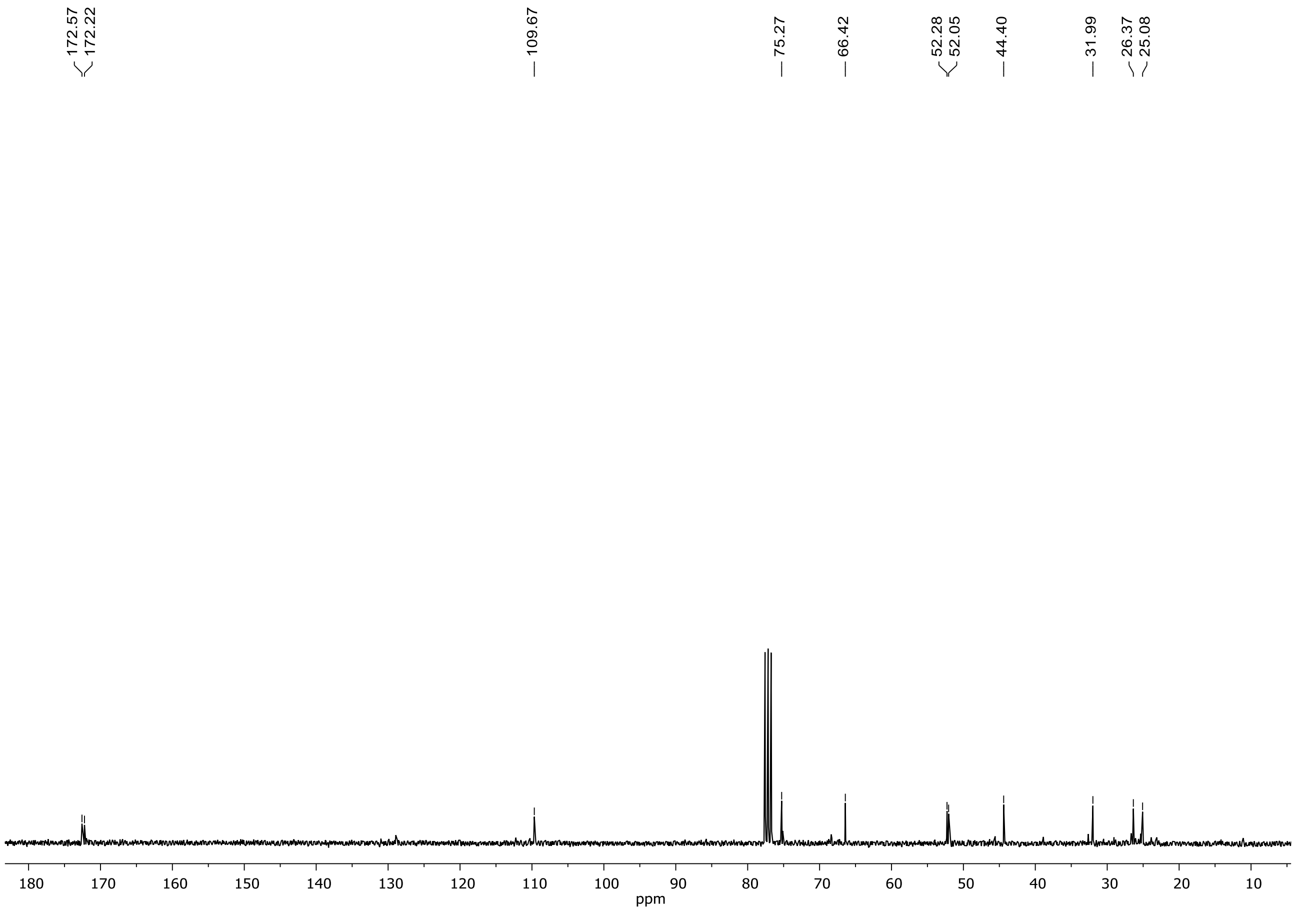

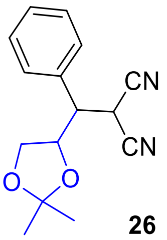

26

^{1}H NMR (300 MHz, Acetone-d_6)

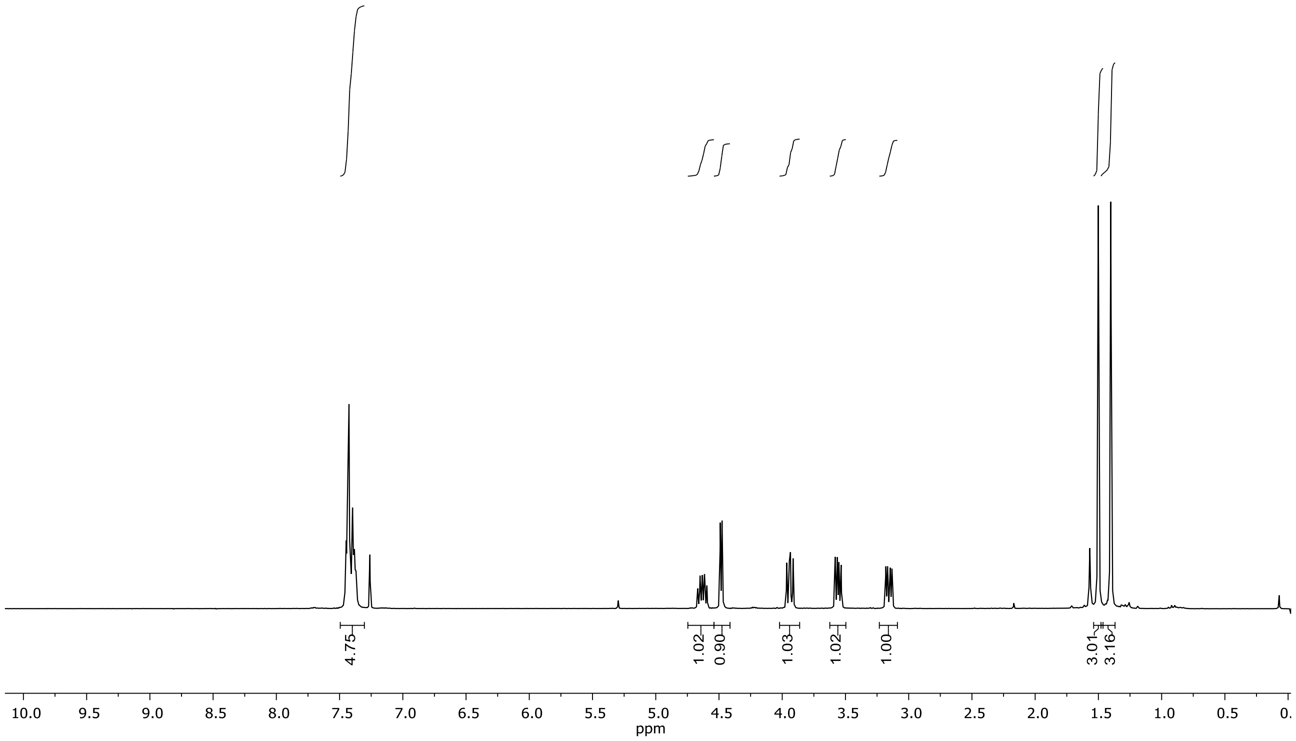

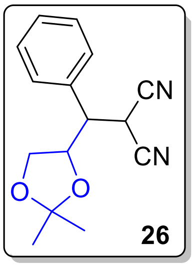

26

^{13}C NMR (75 MHz, Acetone-d_6)

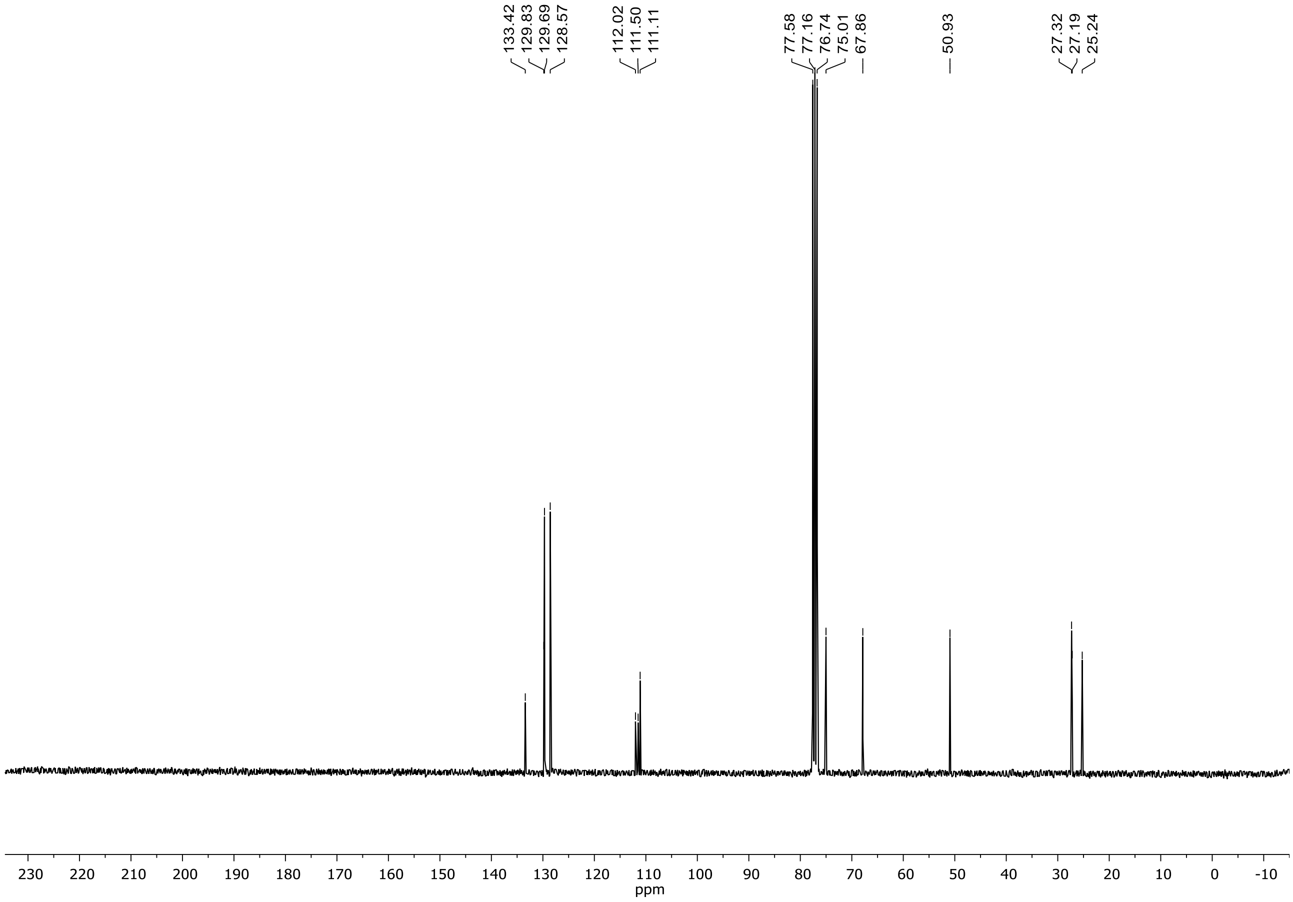

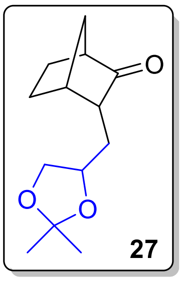

27

^{1}H NMR (300 MHz, chloroform-*d*)

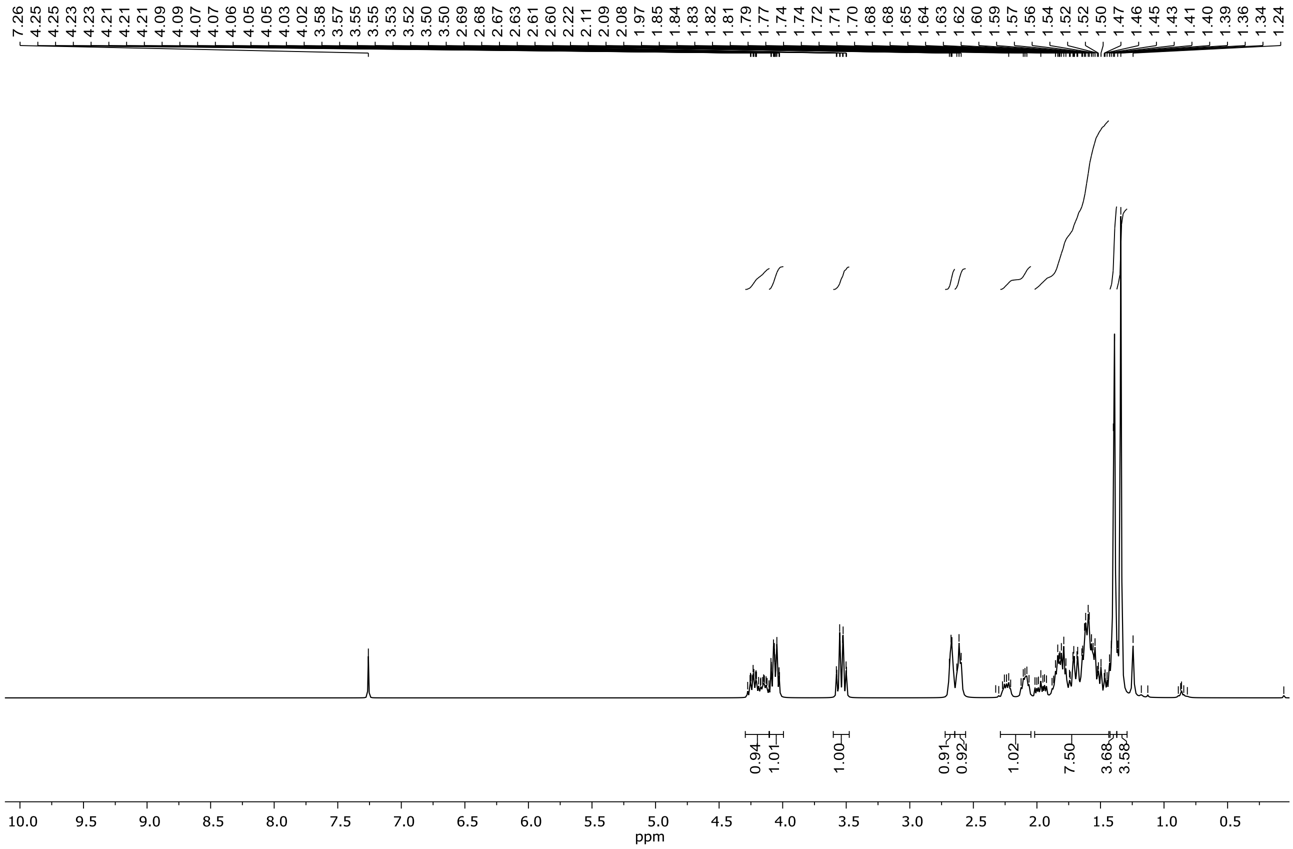

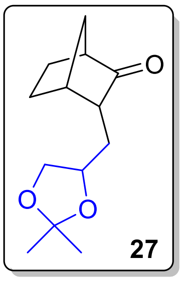

27

^{13}C NMR (75 MHz, chloroform-*d*)

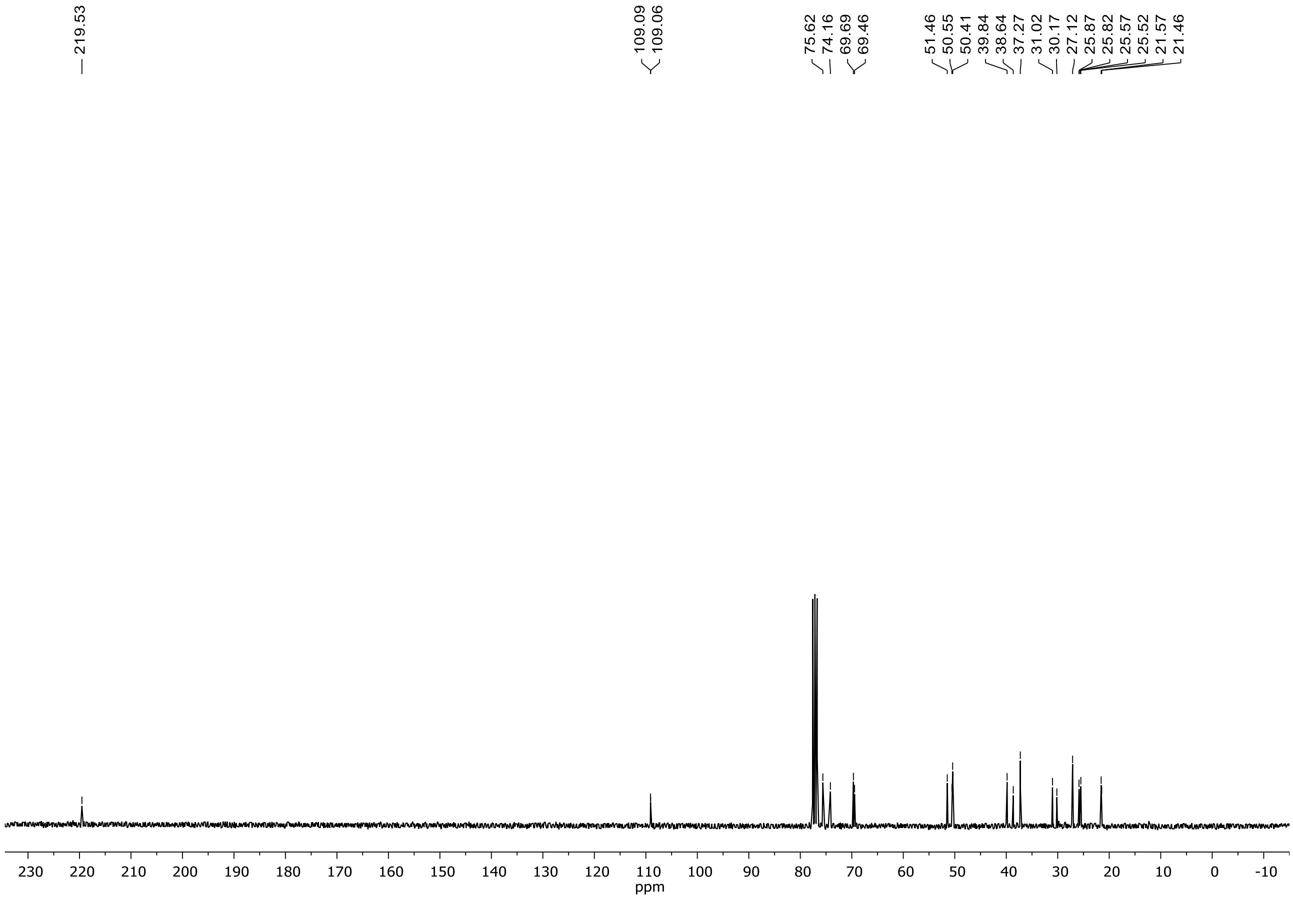

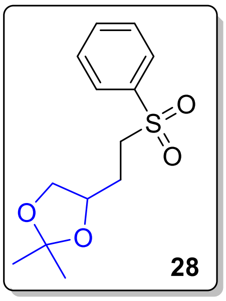

28

^{1}H NMR (300 MHz, chloroform-*d*)

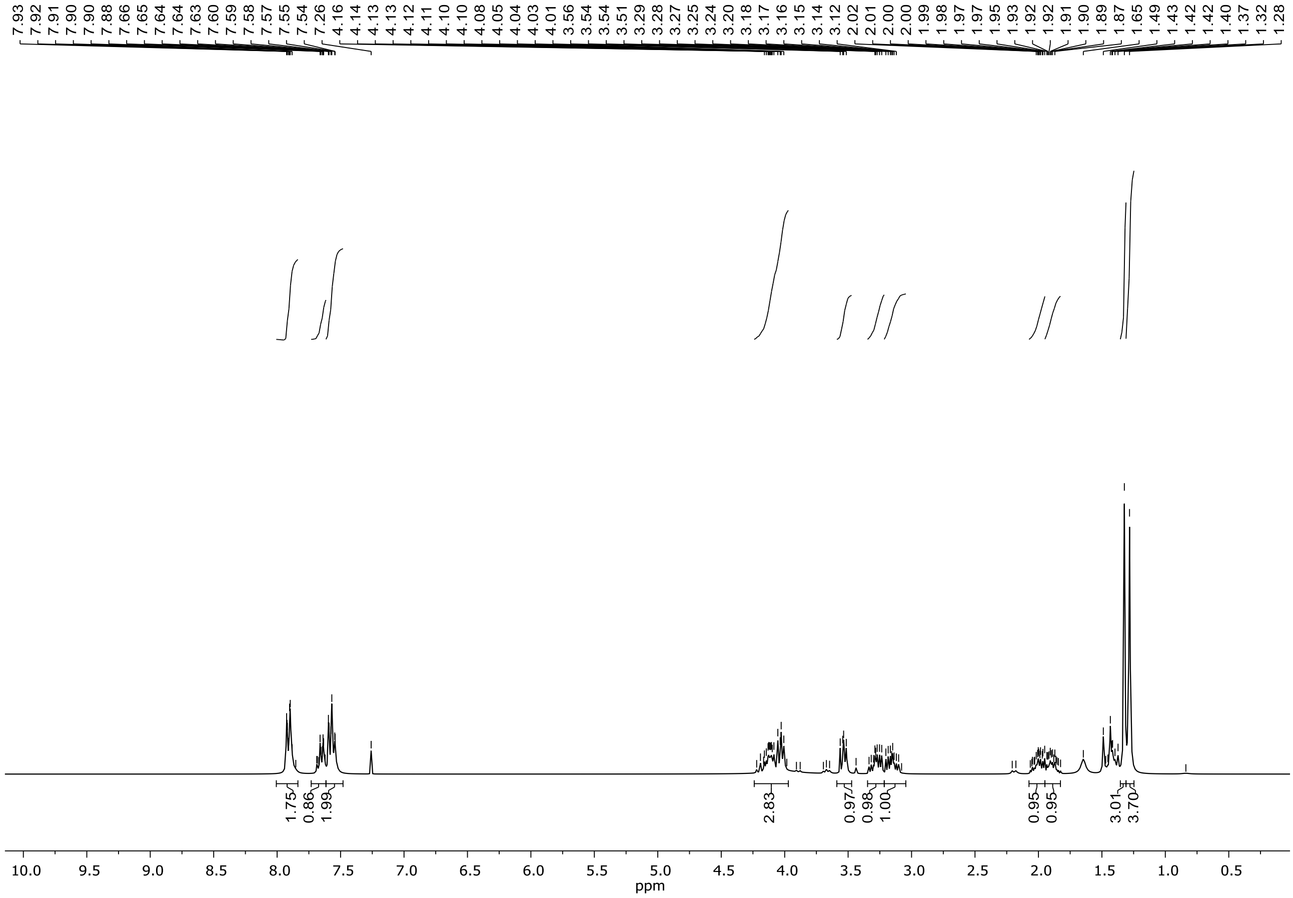

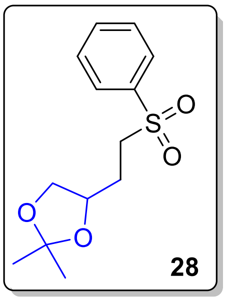

28

^{13}C NMR (75 MHz, chloroform-*d*)

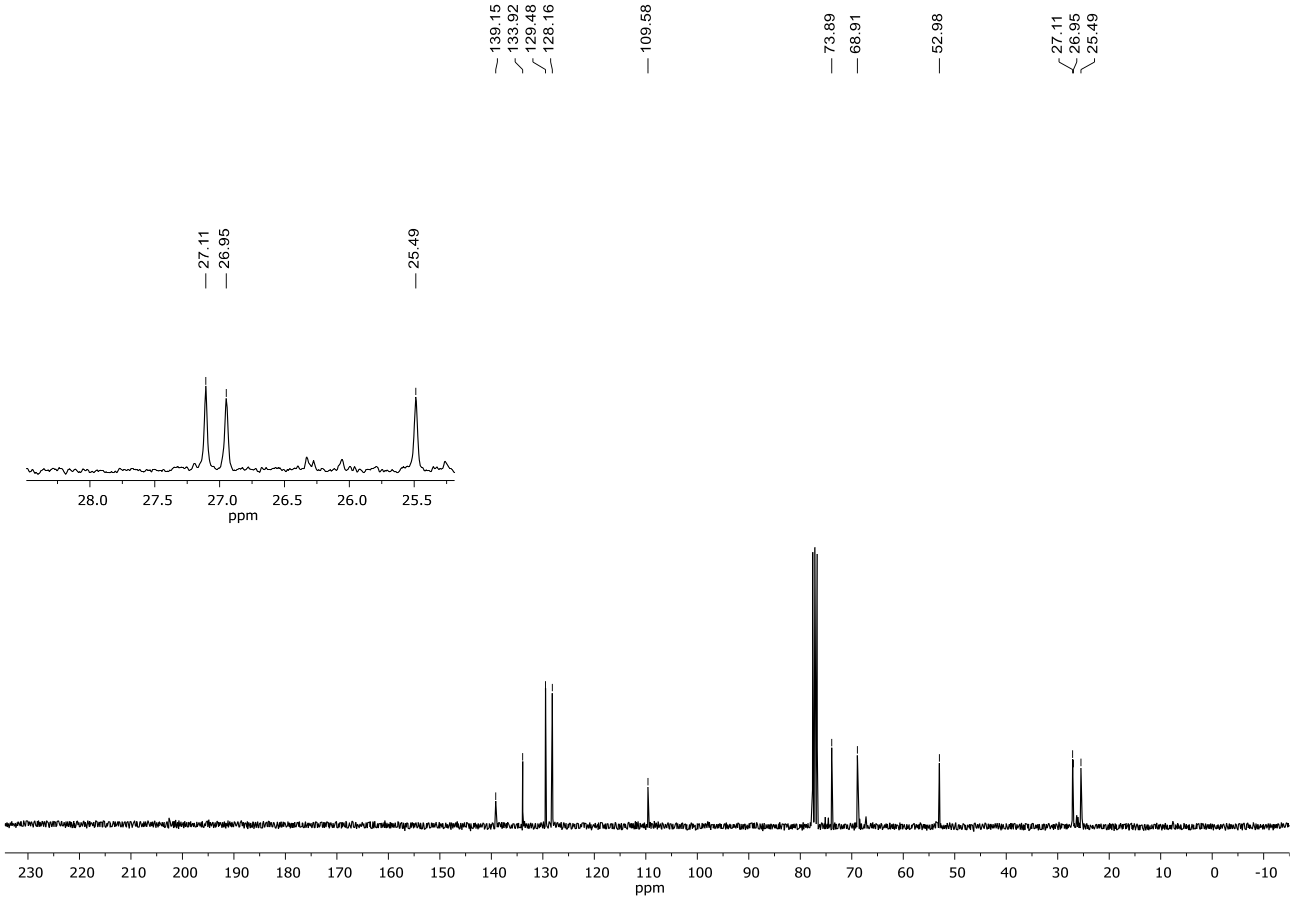

Supplement: Supplementary file 1 — cs2c03768_si_001.pdf [file cs2c03768_si_001.pdf]
